# First-Order Theory of Rewriting for Linear Variable-Separated Rewrite Systems: Automation, Formalization, Certification

**DOI:** 10.1007/s10817-023-09661-7

**Published:** 2023-04-06

**Authors:** Aart Middeldorp, Alexander Lochmann, Fabian Mitterwallner

**Affiliations:** Department of Computer Science, University of Innsbruck, Innsbruck, Austria

**Keywords:** Term rewriting, First-order theory, Tree automata, Formalization

## Abstract

The first-order theory of rewriting is decidable for linear variable-separated rewrite systems. We present a new decision procedure which is the basis of FORT, a decision and synthesis tool for properties expressible in the theory. The decision procedure is based on tree automata techniques and verified in Isabelle. Several extensions make the theory more expressive and FORT more versatile. We present a certificate language that enables the output of FORT to be certified by the certifier FORTify generated from the formalization, and we provide extensive experiments.

## Introduction

Many properties of rewrite systems can be expressed as logical formulas in the first-order theory of rewriting. This theory is decidable for the class of linear variable-separated rewrite systems, which includes all ground rewrite systems. The decision procedure is based on tree automata techniques and goes back to Dauchet and Tison [10]. It is implemented in FORT [46, 48], which takes as input one or more rewrite systems $\mathcal {R}_0$, $\mathcal {R}_1$, $\dots$ and a formula $\varphi$, and determines whether the rewrite systems satisfy the property expressed by $\varphi$, in which case it reports yes or no. FORT may not reach a conclusion due to limited resources.

For properties related to confluence and termination, designated competitions (CoCo [41], termCOMP [23]) of software tools take place regularly. Occasionally, yes/no conflicts appear. Since the participating tools typically couple a plethora of techniques with sophisticated search strategies, human inspection of the output of tools to determine the correct answer is often not feasible. Hence certified categories were created in which tools must output a formal certificate. This certificate is verified by  [53], an automatically generated Haskell program using the code generation feature of Isabelle. This requires not only that the underlying techniques are formalized in Isabelle, but the formalization must be executable for code generation to apply. During the time-consuming formalization process, mistakes in papers are sometimes brought to light. An additional outcome is that formalization efforts may give rise to simpler and more efficient constructions and algorithms.

Since 2017 we are concerned with the question of how to ensure the correctness of the answers produced by FORT. The certifier  supports a great many techniques for establishing concrete properties like termination and confluence, but the formalizations in the underlying Isabelle Formalization of Rewriting ()^1^ are orthogonal to the ones required for supporting the decision procedure underlying FORT. We present a certificate language which is rich enough to express the various automata operations in decision procedures for the first-order theory of rewriting as well as numerous predicate symbols that may appear in formulas in this theory. FORTify, the verified Haskell program obtained from the Isabelle formalization, validates certificates in this language.

The decision procedure implemented in FORT and formalized in Isabelle is based on three different tree automata models. We use standard bottom-up tree automata to represent various sets of ground terms. For (most) binary relations on ground terms, we use *anchored* ground tree transducers. These are a simplification of the ground tree transducers used in the literature [8–10, 12, 18] with better closure properties, reducing the number of constructions needed to represent the first-order theory of rewriting. Some of these closure properties are proved (and formalized) using the simple but equivalent class of *pair automata*. The third model are standard tree automata operating on a different signature in order to represent *n*-ary relations on ground terms, for arbitrary *n* (including $n = 2$). In the next section we present the basic definitions. Section 3 introduces the first-order theory of rewriting. In Sect. 4 we introduce in a systematic way several context closure operations on binary relations that are used to represent the binary predicates in the first-order theory of rewriting. Detailed proofs of the various results concerning the three tree automata models that are required for the decision procedure are presented in Sect. 5. Many of the results and tree automata constructions in this section are well-known, but are included for completeness and because the implementation in FORT and the subsequent formalization are directly based on them. Tree automata operate on ground terms. In Sect. 6 we present the formalized signature extension results that allow to reduce certain properties on arbitrary terms to properties on ground terms. In Sect. 7 the decision and synthesis modes of FORT are described, and a new undecidability proof related to the latter is presented. We also discuss the representation of formulas in certificates and the certificate language, and we explain how certificates are validated by FORTify, the verified Haskell program obtained from the Isabelle formalization. Experimental results are presented in Sect. 8, before we conclude in Sect. 9. In an appendix the input syntax and the interface of the tools is presented.

The formalization is based on Isabelle/HOL. Our contribution is split into three parts, which are published as separate entries in the Archive of Formal Proofs.^2^ The first part [35] contains general results about bottom-up tree automata, ported from , extended with constructions and results about anchored ground tree transducers, pair automata, and regular relation automata. The second part [33] formalizes primitive constructions needed to decide the first-order theory of rewriting. Moreover, it connects the logical semantic entailment of first-order formulas to regular tree languages. This connection gives rise to a natural description of the decision procedure. The specification allows tool authors to generate certificates (which can be viewed as a formal proof claim using appropriate automata construction for the corresponding logical connectives and predicates). We rely on the code generation facility of Isabelle/HOL to obtain the certifier FORTify that is able to verify the integrity of such certificates. The third part [32] is independent, and covers the results in Sect. 6.

The formalization can be accessed via the following links:

- https://www.isa-afp.org/entries/Regular_Tree_Relations.html
- https://www.isa-afp.org/entries/FO_Theory_Rewriting.html
- https://www.isa-afp.org/entries/Rewrite_Properties_Reduction.html

Most definitions, theorems, and lemmata in this paper directly correspond to the formalization. These are indicated by the  symbol, which links to an HTML rendering of our formalization, for those who like to dive right into the actual Isabelle code. In the running text (traditional) proof details are given.

This article combines and extends earlier papers that appeared in conference and informal workshop proceedings. These cover system descriptions of earlier versions of FORT [46, 48], formalization and certification aspects [22, 34, 36, 42], as well as results for dealing with properties on non-ground terms [37, 38, 47]. Many new examples to illustrate the various constructions were added and the presentation is self-contained. The efficiency improvements described in Sect. 7 are new. The same is true for the undecidability result in Sect. 7.5. Also several of the experiments that we present in Sect. 8 have not been described before.

## Preliminaries

In this preliminary section we recall basic definitions and notations of term rewriting [3] and tree automata [8].

### Term Rewriting

We assume a finite signature $\mathcal {F}$ containing at least one constant symbol and a disjoint set of variables $\mathcal {V}$. The set of terms built up from $\mathcal {F}$ and $\mathcal {V}$ is denoted by $\mathcal {T}(\mathcal {F},\mathcal {V})$, while $\mathcal {T}(\mathcal {F})$ denotes the (non-empty) set of ground terms. The set of variables occurring in a term *t* is denoted by $\mathcal {V}\textsf{ar}(t)$. A term is linear if it does not contain multiple occurrences of the same variable. Positions are strings of positive integers which are used to address subterms. The set of positions in a term *t* is denoted by $\mathcal {P}\textsf{os}(t)$ and the root position by $\varepsilon$. The function symbol at position $p \in \mathcal {P}\textsf{os}(t)$ is denoted by *t*(*p*) and $t[u]_p$ denotes the result of replacing the subterm $t|_p$ of *t* at position *p* by the term *u*. The height $\textsf{height}(t)$ of a term *t* is the length of a longest position in $\mathcal {P}\textsf{os}(t)$. A substitution is a mapping $\sigma$ from variables to terms and $t\sigma$ denotes the result of applying $\sigma$ to a term *t*. A context *C* is a term that contains exactly one hole, denoted by the special constant $\square \notin \mathcal {F}$. We write *C*[*t*] for the result of replacing the hole in *C* by the term *t*. A term rewrite system (TRS) $\mathcal {R}$ is a set of rules $\ell \rightarrow r$ between terms $\ell , r \in \mathcal {T}(\mathcal {F},\mathcal {V})$. A TRS $\mathcal {R}$ is linear if its rewrite rules consist of linear terms. We call $\mathcal {R}$ *variable-separated* if $\mathcal {V}\textsf{ar}(\ell ) \cap \mathcal {V}\textsf{ar}(r) = \varnothing$ for every $\ell \rightarrow r \in \mathcal {R}$.

In this paper we are concerned with finite, linear, variable-separated TRSs $\mathcal {R}$ and we (mostly) consider rewriting on ground terms: $t \rightarrow _{\mathcal {R}}u$ for ground terms *t*, *u* if there exist a context *C*, a rewrite rule $\ell \rightarrow r \in \mathcal {R}$, and a substitution $\sigma$ such that $t = C[\ell \sigma ]$ and $u = C[r\sigma ]$. We write $\rightarrow _{\mathcal {R}}^{*}$ for the reflexive and transitive closure of $\rightarrow _{\mathcal {R}}$. Further relations on terms will be introduced in the next section. We drop the subscript $\mathcal {R}$ when it can be inferred from the context. A ground normal form is a ground term *t* such that $t \rightarrow _{\mathcal {R}}u$ for no term *u*. We write $\textsf{NF}(\mathcal {R})$ for the set of ground normal forms of $\mathcal {R}$.

#### Example 1

We use the TRS $\mathcal {R}$ consisting of the rewrite rules

$$\begin{aligned} \textsf{a}&\rightarrow \textsf{b}&\textsf{f}(\textsf{a})&\rightarrow \textsf{b}&\textsf{g}(\textsf{a},x)&\rightarrow \textsf{f}(\textsf{a}) \end{aligned}$$

over the signature $\mathcal {F}= \{\textsf{a},\textsf{b},\textsf{f},\textsf{g}\}$ as leading example in this paper. We have

$$\begin{aligned} \textsf{f}(\textsf{g}(\textsf{a},\textsf{b}))&\,\rightarrow _{\mathcal {R}}\, \textsf{f}(\textsf{f}(\textsf{a})) \,\rightarrow _{\mathcal {R}}\, \textsf{f}(\textsf{b}) \end{aligned}$$

with ground normal form $\textsf{f}(\textsf{b})$.

### Tree Automata

A (finite bottom-up) tree automaton $\mathcal {A}= (\mathcal {F},Q,Q_f,\Delta )$ consists of a finite signature $\mathcal {F}$, a finite set *Q* of states, disjoint from $\mathcal {F}$, a subset $Q_f \subseteq Q$ of final states, and a set of transition rules $\Delta$. Every transition rule has one of the following two shapes:

- $f(p_1,\dotsc ,p_{n}) \rightarrow q$ with $f \in \mathcal {F}$ and $p_1,\dotsc ,p_{n}, q \in Q$, or
- $$p \rightarrow q$$ with $$p, q \in Q$$.

Transition rules of the second shape are called $\varepsilon$-transitions. Transition rules can be viewed as rewrite rules between ground terms in $\mathcal {T}(\mathcal {F}\cup Q,\mathcal {V})$. The induced rewrite relation is denoted by $\rightarrow _{\Delta }$ or $\rightarrow _{\!\mathcal {A}}$. A ground term $t \in \mathcal {T}(\mathcal {F})$ is accepted by $\mathcal {A}$ if $t \rightarrow _{\Delta }^{*} q$ for some $q \in Q_f$. The set of all accepted terms is denoted by $L(\mathcal {A})$ and a set *L* of ground terms is regular if $L = L(\mathcal {A})$ for some tree automaton $\mathcal {A}$. A tree automaton $\mathcal {A}$ is deterministic if there are no $\varepsilon$-transitions and no two transition rules with the same left-hand side. We say that $\mathcal {A}$ is completely defined if it contains a transition rule with left-hand side $f(p_1,\dotsc ,p_{n})$ for every *n*-ary function symbol *f* and every combination $p_1,\dotsc ,p_{n}$ of states. All regular sets are accepted by a completely defined, deterministic tree automaton. The class of regular sets is effectively closed under Boolean operations. Moreover, membership and emptiness are decidable.

For relations on ground terms two different types of automata are used. The first one is restricted to binary relations. A ground tree transducer (GTT for short) is a pair $\mathcal {G}= (\mathcal {A},\mathcal {B})$ of tree automata over the same signature $\mathcal {F}$. Let *s* and *t* be ground terms in $\mathcal {T}(\mathcal {F})$. We say that the pair (*s*, *t*) is accepted by $\mathcal {G}$ if  for some term $u \in \mathcal {T}(\mathcal {F}\cup Q)$. Here *Q* is the combined set of states of $\mathcal {A}$ and $\mathcal {B}$. The set of all such pairs is denoted by $L(\mathcal {G})$. Observe that $L(\mathcal {G})$ is a binary relation on $\mathcal {T}(\mathcal {F})$. A binary relation ${\bowtie }$ on ground terms is a GTT relation if there exists a GTT $\mathcal {G}$ such that ${{\bowtie }} = L(\mathcal {G})$. In FORT we deal with *anchored* GTTs, which are GTTs with a different acceptance condition: A pair (*s*, *t*) of ground terms is accepted by an anchored GTT $\mathcal {G}$ if  for some (common) state *q*. The set of all such pairs is denoted by $L_a(\mathcal {G})$. It can be shown that the resulting language class coincides with binary $\textit{Rec}_\times$ which is defined in [8, Sect. 3.2.1] as the class of finite unions of Cartesian products of regular sets. The more operational view above benefits the developments described in subsequent sections. We obviously have $L_a(\mathcal {G}) \subseteq L(\mathcal {G})$. Anchored GTT relations have the advantage that they can represent the root-step relation $\rightarrow _{\varepsilon }$, which is not possible with GTT relations as the latter are always reflexive. Moreover, they have better closure properties than GTT relations. When we speak of “anchored GTTs”, we always have $L_a(\mathcal {G})$ in mind.

The second method for representing relations on ground terms uses standard tree automata operating on an encoding of the relation as a set of ground terms over a special signature. For a signature $\mathcal {F}$ and $n \geqslant 0$ we let $\mathcal {F}^{(n)} = (\mathcal {F}\cup \{\bot \})^n$. Here, $\bot \notin \mathcal {F}$ is a fresh constant. The arity of a symbol $f_1 \ldots f_n \in \mathcal {F}^{(n)}$ is the maximum of the arities of $f_1,\dotsc ,f_{n}$ and 0 if $n = 0$. Given *n* terms $t_1,\dotsc ,t_{n} \in \mathcal {T}(\mathcal {F})$, the term $\langle t_1,\dotsc ,t_{n}\rangle$ is the unique term $u \in \mathcal {T}(\mathcal {F}^{(n)})$ such that $\mathcal {P}\textsf{os}(u) = \mathcal {P}\textsf{os}(t_1) \cup \cdots \cup \mathcal {P}\textsf{os}(t_n)$ and $u(p) = f_1 \cdots f_n$ where $f_i = t_i(p)$ if $p \in \mathcal {P}\textsf{os}(t_i)$ and $\bot$ otherwise, for all positions $p \in \mathcal {P}\textsf{os}(u)$. If $n = 0$ then $\mathcal {P}\textsf{os}(u) = \{\varepsilon \}$ and $u(\varepsilon )$ is the empty sequence.

#### Example 2

For $\mathcal {F}= \{\textsf{a},\textsf{b},\textsf{f},\textsf{g}\}$ in Example 1 we have

$$\begin{aligned} \langle \textsf{g}(\textsf{a},\textsf{f}(\textsf{b})), \textsf{f}(\textsf{a})\rangle&\,=\, \textsf{gf}(\textsf{aa},\textsf{f}\bot (\textsf{b}\bot )) \in \mathcal {T}(\mathcal {F}^{(2)}) \\ \langle \textsf{a}, \textsf{f}(\textsf{f}(\textsf{b})),\textsf{g}(\textsf{b},\textsf{a})\rangle&\,=\, \textsf{afg}(\bot \textsf{fb}(\bot \textsf{b}\bot ),\bot \bot \textsf{a}) \in \mathcal {T}(\mathcal {F}^{(3)}) \end{aligned}$$

An *n*-ary relation *R* on $\mathcal {T}(\mathcal {F})$ is regular if its encoding $\{\langle t_1,\dotsc ,t_{n}\rangle \mid (t_1,\dotsc ,t_{n}) \in R\}$ is regular. The class of all *n*-ary regular relations is denoted by $\textsf{RR}_{n}$. Every (anchored) GTT relation belongs to $\textsf{RR}_{2}$. The well-known construction (presented later in the proof of Theorem 10) is used to decide membership for anchored GTT relations.

## First-Order Theory of Rewriting

We consider first-order logic over a language $\mathcal {L}$ without function symbols. The language contains the following binary predicate symbols:Further predicate symbols will be added to $\mathcal {L}$ later in this paper. As models we consider finite linear variable-separated TRSs $(\mathcal {F},\mathcal {R})$ such that the set of ground terms $\mathcal {T}(\mathcal {F})$ is non-empty, which is equivalent to the requirement that the signature $\mathcal {F}$ contains at least one constant symbol. The set of ground terms serves as domain for the variables in formulas over $\mathcal {L}$. The interpretation of the predicate symbol $\rightarrow$ in $(\mathcal {F},\mathcal {R})$ is the one-step rewrite relation $\rightarrow _{\mathcal {R}}$ over $\mathcal {T}(\mathcal {F})$, $\rightarrow ^{*}$ denotes its transitive-reflexive closure, and $=$ is interpreted as equality on ground terms.

Variable-separated TRSs appear naturally when approximating TRSs that satisfy the usual variable restriction ($\mathcal {V}\textsf{ar}(r) \subseteq \mathcal {V}\textsf{ar}(\ell )$ for every rewrite rule $\ell \rightarrow r$), to achieve regularity of the set of reachable terms starting from a regular set of ground terms. The support for linear variable-separated TRSs opens up the possibility of using FORT to compute dependency graphs based on the non-variable approximation for termination analysis [40], check infeasibility of conditional critical pairs for confluence analysis of conditional TRSs [51], and compute needed redexes based on the strong and non-variable approximations for the analysis of optimal normalizing strategies [18].

The following example gives an idea of the decision procedure for the first-order theory of rewriting. It shows how (closure) operations on tree automata and GTTs are used to obtain tree automata, each of which represent tuples of ground terms satisfying subformulas of the formula of interest. These operations are presented in Sect. 5 together with correctness proofs that have been formalized.

### Example 3

Consider the formula

$$\begin{aligned} \varphi = \forall \,s\,\exists \,t\,(s \rightarrow ^{*} t \,\wedge \, \lnot \,\exists \,u\,(t \rightarrow u)) \end{aligned}$$

which expresses the normalization property of TRSs. To determine whether a given linear variable-separated TRS $\mathcal {R}$ over a signature $\mathcal {F}$ satisfies $\varphi$, we construct automata for the subterms of the formula in a bottom-up fashion. We start with an $\textsf{RR}_1$ automaton $\mathcal {A}_1$ that accepts the ground normal forms in $\mathcal {T}(\mathcal {F})$, using an algorithm first described in [6] and covered in Sect. 5.4: 
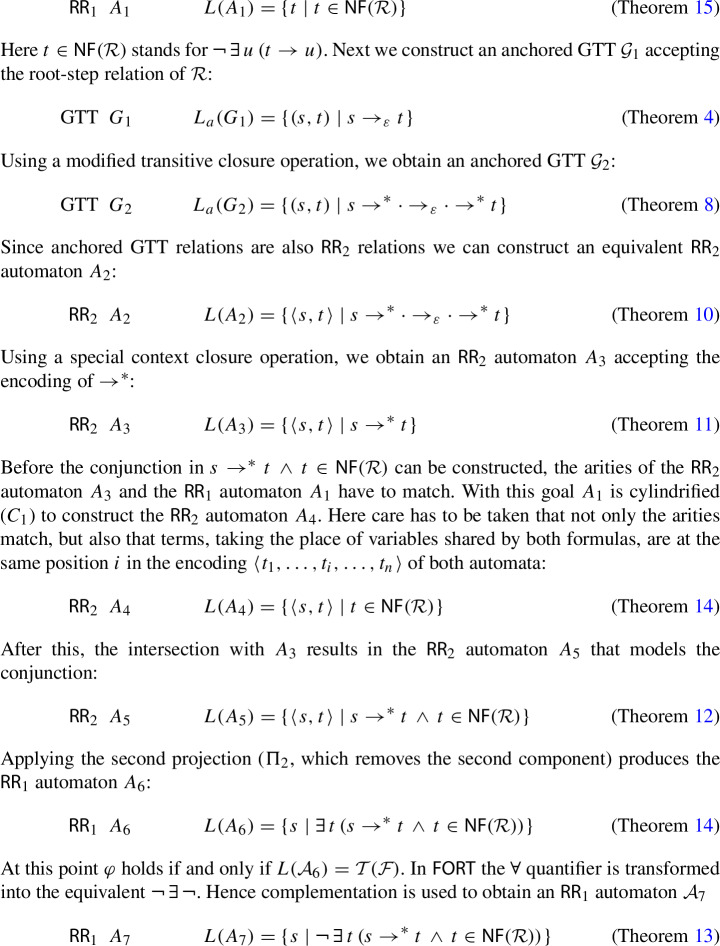


and the existential quantifier is implemented using projection. This gives an $\textsf{RR}_0$ automaton $\mathcal {A}_8$ which either accepts the empty relation $\varnothing$ or the singleton set $\{()\}$ consisting of the nullary tuple (). The outermost negation gives rise to another complementation step. The final $\textsf{RR}_0$ automaton $\mathcal {A}_9$ is tested for emptiness: $L(\mathcal {A}_9) = \varnothing$ if and only the TRS $\mathcal {R}$ does not satisfy $\varphi$.

In order to express termination in the first-order theory of rewriting, we extend $\mathcal {L}$ with the binary predicate symbol $\rightarrow ^+$ (which denotes the transitive closure of $\rightarrow$) and the unary predicate defined below (which goes back to a technical report by Dauchet and Tison [11]).

### Definition 1

Let ${\bowtie }$ be an arbitrary binary relation on $\mathcal {T}(\mathcal {F})$. We write $\textsf{INF}_{\bowtie }$ for the set $\{t \in \mathcal {T}(\mathcal {F})\mid t \mathrel {{\bowtie }} u \text { for infinitely many terms }u \in \mathcal {T}(\mathcal {F})\}$.

If we instantiate $\textsf{INF}_{\bowtie }$ by taking ${\bowtie }= {\rightarrow ^{*}}$, we obtain the predicate $\textsf{INF}_{\rightarrow ^{*}}$ that is satisfied by ground terms that have infinitely many reducts. By forbidding cycles, we obtain the formula

$$\begin{aligned} \lnot \,\exists \,t~(\textsf{INF}_{\rightarrow ^{*}}(t) \,\vee \, t \rightarrow ^{+} t) \end{aligned}$$

that expresses termination of finite variable-separated TRSs.

The grammar in Fig.  lists the formalized (closure) operations for the *predicates* in the first-order theory of rewriting. Here *A* are anchored GTT relations, *R* are $\textsf{RR}_{2}$ relations, and *T* are regular sets of ground terms. Some of the operations will be introduced in subsequent sections.

**Fig. 1:**
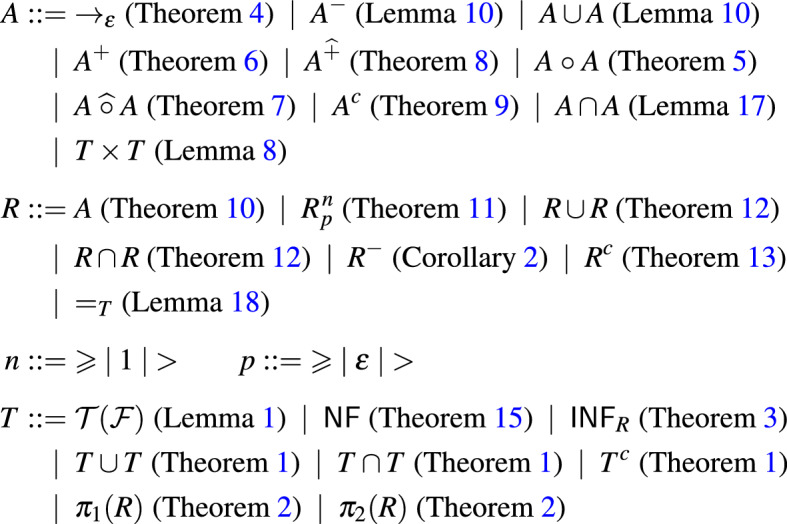
Automata operations for the predicates in the first-order theory of rewriting

The TRS $\mathcal {R}$ enters the picture in three places. First of all, $\rightarrow _{\varepsilon }$ is the root-step relation of $\mathcal {R}$. Secondly, $\textsf{NF}$ denotes the set of ground normal forms of $\mathcal {R}$. Finally, $\mathcal {T}(\mathcal {F})$ denotes the set of ground terms, which depends on the signature $\mathcal {F}$ of $\mathcal {R}$.

Every atomic subformula (predicate) will be represented as an $\textsf{RR}_{1}$ or $\textsf{RR}_{2}$ relation. The logical structure of *formulas* in the first-order theory of rewriting is taken care of by additional closure operations on $\textsf{RR}_{n}$ relations.

## Context Operations

In the next section we describe formalized automata constructions to decide the first-order theory of rewriting. To save considerable formalization efforts, we introduce a few primitives that operate on binary relations that are accepted by various kinds of tree automata. These primitives are sufficient to generate all binary rewrite relations supported by FORT. For defining the semantics of the primitives, we introduce some context operations on binary relations in this section.

### Definition 2

Let $\mathcal {F}$ be a signature. A *multi-hole context* is an element of $\mathcal {T}(\mathcal {F}\uplus \{\square \})$ where $\square$ is a fresh constant symbol, called *hole*. If *C* is a multi-hole context with $n \geqslant 0$ holes and $t_1,\dotsc ,t_{n}$ are terms in $\mathcal {T}(\mathcal {F})$ then $C[t_1,\dotsc ,t_{n}]$ denotes the term in $\mathcal {T}(\mathcal {F})$ obtained from *C* by replacing the holes from left to right with $t_1,\dotsc ,t_{n}$. We write $\mathcal {C}$ for the set of all multi-hole contexts. Given a binary relation ${\bowtie }$ on ground terms in $\mathcal {T}(\mathcal {F})$ and a set of multi-hole contexts $\mathcal {D}\subseteq \mathcal {C}$, we write $\mathcal {D}({\bowtie })$ for the relation $\{(C[t_1,\dotsc ,t_{n}],C[u_1,\dotsc ,u_{n}]) \mid C \in \mathcal {D}\text { has }n \text { holes and }t_i \mathrel {{\bowtie }} u_i \text { for all }1 \leqslant i \leqslant n\}$.

We consider two ways to restrict multi-hole contexts: restricting the number of holes and restricting the position of the holes.

- We denote the set of multi-hole contexts with exactly one hole by $\mathcal {C}^1$. The set of multi-hole contexts with at least one hole is denoted by $\mathcal {C}^>$. Moreover $\mathcal {C}^\geqslant$ simply denotes $\mathcal {C}$.
- We denote the set of multi-hole contexts with the property that every hole occurs below the root position by $\mathcal {C}_>$. This includes the set $\mathcal {T}(\mathcal {F})$ of ground terms (which are multi-hole contexts without holes). Similarly, $\mathcal {C}_\varepsilon$ denotes the set of multi-hole contexts with the property that every hole occurs at the root position. So $\mathcal {C}_\varepsilon = \{\square \} \cup \mathcal {T}(\mathcal {F})$. Moreover, $\mathcal {C}_\geqslant$ simply denotes $\mathcal {C}$.

By combining both types of restrictions, we obtain nine ways for defining new binary relations.

### Definition 3

Let ${\bowtie }$ be a binary relation on $\mathcal {T}(\mathcal {F})$. Given a number constraint $n \in \{{\geqslant }, 1, {>}\}$ and a position constraint $p \in \{{\geqslant }, \varepsilon , {>}\}$, we define the binary relation ${\bowtie }^{n}_{p}$ on $\mathcal {T}(\mathcal {F})$ as $(\mathcal {C}^n \cap \mathcal {C}_p)({\bowtie })$.

Note that ${\bowtie }_\varepsilon ^\geqslant = {\bowtie }^=$ and ${\bowtie }_\varepsilon ^1 = {\bowtie }_\varepsilon ^> = {\bowtie }$, for any ${\bowtie }$. Here ${\bowtie }^= = {\bowtie }\cup \{=\}$ denotes the reflexive closure of ${\bowtie }$.

### Example 4

Recall the TRS $\mathcal {R}$ from our leading example and consider the multi-hole contexts

$$\begin{aligned} C_1&= \square&C_2&= \textsf{f}(\square )&C_3&= \textsf{g}(\square ,\textsf{a})&C_4&= \textsf{g}(\square ,\square )&C_5&= \textsf{f}(\textsf{a}) \end{aligned}$$

We have $C_1, C_2, C_3 \in \mathcal {C}^1$, $C_1, C_2, C_3, C_4 \in \mathcal {C}^>$, $C_1, C_5 \in \mathcal {C}_\varepsilon$, and $C_2, C_3, C_4, C_5 \in \mathcal {C}_>$. Moreover, $(C_2[\textsf{a}],C_2[\textsf{b}]) \in (\rightarrow _{\mathcal {R}})_>^1$ and $(C_4[\textsf{a},\textsf{a}],C_4[\textsf{b},\textsf{b}]) \notin (\rightarrow _{\mathcal {R}})_>^1$.

Because $\mathcal {C}_\geqslant = \mathcal {C}^\geqslant = \mathcal {C}$, the relation ${\bowtie }_\geqslant ^\geqslant$ is the multi-hole context closure of ${\bowtie }$ . Using the root-step relation $\rightarrow _{\varepsilon }$ induced by a linear, variable-separated TRS $\mathcal {R}$ as ${\bowtie }$, we obtain eight different relations for $(\rightarrow _{\varepsilon })_p^n$:Here  denotes a parallel step (which is the multi-hole context closure of $\rightarrow$),  a non-empty parallel step,  a parallel step where only redexes below the root are contracted, and  a non-empty parallel step where only redexes below the root are contracted.

### Example 5

Consider the term pairs $\pi _1 = (\textsf{g}(\textsf{a},\textsf{a}),\textsf{g}(\textsf{b},\textsf{b}))$, $\pi _2 = (\textsf{g}(\textsf{a},\textsf{a}),\textsf{f}(\textsf{a}))$, and $\pi _3 = (\textsf{g}(\textsf{a},\textsf{a}),\textsf{g}(\textsf{a},\textsf{a}))$. We have , , , and .

## Formalized Tree Automata Constructions

In this section we present constructions on tree automata and (anchored) GTTs that are required for the decision procedure. Most of the results are known [8]. We give explicit proofs, providing detailed constructions that form the basis of the implementation of the decision procedure in FORT as well as the formalization in Isabelle.

Let $\mathcal {A}= (\mathcal {F},Q,Q_f,\Delta )$ be a tree automaton. A state $q \in Q$ is *reachable* if $t \rightarrow _{\Delta }^{*} q$ for some term $t \in \mathcal {T}(\mathcal {F})$. We say that *q* is *productive* if $C[q] \rightarrow _{\Delta }^{*} q_f$ for some ground context *C* and final state $q_f \in Q_f$. The automaton $\mathcal {A}$ is *trim* if all states are both reachable and productive. Any tree automaton can be transformed into an equivalent trim automaton. This result has been formalized in  by Felgenhauer and Thiemann [21]. The construction preserves determinism. The following results are well-known.

### Lemma 1

$(T~\mathsf {::=}~\mathcal {T}(\mathcal {F}))$  The set of ground terms over a finite signature $\mathcal {F}$ is regular. 

### Theorem 1

$(T~\mathsf {::=}~T \cup T \mid T \cap T \mid T^c)$ The class of regular sets is effectively closed under union, intersection, and complement. 

Before we turn to the infinity predicate ($T~\mathsf {::=}~\textsf{INF}_R$), we present an important closure operation on regular relations. Other closure operations will be presented in Sect. 5.3.

### Definition 4

Let *R* be an *n*-ary relation over $\mathcal {T}(\mathcal {F})$. If $n \geqslant 1$ and $1 \leqslant i \leqslant n$ then the *i*-th projection of *R* is the relation $\Pi _i(R) = \{(t_1,\dots ,t_{i-1},t_{i+1},\dots ,t_n) \mid (t_1,\dotsc ,t_{n}) \in R\}$.

Note that $\Pi _1$ removes the first component of an $\textsf{RR}_{n}$ relation. So for a binary regular relation *R*, $\Pi _1(R)$ coincides with $\pi _2(R)$ in the grammar in Fig. 1.

### Theorem 2

($T~\mathsf {::=}~\pi _1(R) \mid \pi _2(R)$)  The class of regular relations is effectively closed under projection. 

### Proof

(construction) Let $\mathcal {A}= (\mathcal {F}^{(n)},Q,Q_f,\Delta )$ be a tree automaton that accepts $\langle R\rangle$. Assume $n \geqslant 1$ and let $1 \leqslant i \leqslant n$. We construct a tree automaton that accept $\langle \Pi _i(R)\rangle$. We assume that all states of $\mathcal {A}$ are reachable and define $\mathcal {A}_{\Pi _i} = (\mathcal {F}^{(n-1)},Q,Q_f,\Delta _{\Pi _i})$ where $\Delta _{\Pi _i}$ is obtained from $\Delta$ by replacing every transition rule of the form

$$\begin{aligned} f_1 \cdots f_{i-1}f_if_{i+1} \cdots f_n(p_1,\dotsc ,p_{m})&\rightarrow q \end{aligned}$$

with

$$\begin{aligned} f_1 \cdots f_{i-1}f_{i+1} \cdots f_n(p_1,\dotsc ,p_{k})&\rightarrow q \end{aligned}$$

provided $n = 1$ or $f_1 \cdots f_{i-1}f_{i+1} \cdots f_n \ne \bot ^{n-1}$ for $n > 1$. Here $k \leqslant m$ is the arity of $f_1 \cdots f_{i-1}f_{i+1} \cdots f_n$. Epsilon transitions in $\Delta$ are not affected. Note that for $n = 1$ this results in an automaton over the signature containing only a single constant () (the nullary tuple). The proof that $L(\mathcal {A}_{\Pi _i}) = \langle \Pi _i(R)\rangle$ is given at the end of Sect. 5.3. $\square$

### Example 6

Consider the tree automaton $\mathcal {A}= (\mathcal {F}^{(2)},\{0,\dotsc ,6\},\{6\},\Delta )$ with $\mathcal {F}= \{\textsf{a},\textsf{b},\textsf{f},\textsf{g}\}$ and $\Delta$ consisting of the transition rulesThis automaton accepts the encoding of $\rightarrow _{\mathcal {R}}$ on $\mathcal {T}(\mathcal {F})$ induced by the TRS $\mathcal {R}$ consisting of the rewrite rulesFor the first projection we obtain the automaton $\Pi _1(\mathcal {A})$ consisting of the transition rulesNote that the third row of transitions in $\Delta$ disappeared completely. The rule $\textsf{f}\textsf{g}(1,3) \rightarrow 6$ is transformed into $\textsf{g}(1) \rightarrow 6$, so state 3 is dropped. The second projection results in the automaton $\Pi _2(\mathcal {A})$ that accepts the reducible ground terms of $\mathcal {R}$:

We now present a formalized proof of a version of the *pumping lemma* that we need for the infinity predicate $\textsf{INF}_R$ (in the proof of Theorem 3 below).

### Lemma 2

Let $\mathcal {A}= (\mathcal {F},Q,Q_f,\Delta )$ be a tree automaton and $t \rightarrow _{\Delta }^{*} q$ with $t \in \mathcal {T}(\mathcal {F})$ and $q \in Q$. If $\textsf{height}(t) > |Q|$ then there exist contexts $C_1$ and $C_2 \ne \square$, a term *u*, and a state *p* such that $t = C_1[C_2[u]]$, $u \rightarrow _{\Delta }^{*} p$, $C_2[p] \rightarrow _{\Delta }^{*} p$, and $C_1[p] \rightarrow _{\Delta }^{*} q$. 

### Proof

From the assumptions $t \rightarrow _{\Delta }^{*} q$ and $\textsf{height}(t) > |Q|$ we obtain a sequence

$$\begin{aligned} (t_1,\dotsc ,t_{n+1},q_1,\dotsc ,q_{n+1},D_1,\dotsc ,D_{n}) \end{aligned}$$

consisting of ground terms, states, and non-empty contexts with $n > |Q|$ such that

- $$t_i \rightarrow _{\Delta }^{*} q_i$$ for all $$i \leqslant n + 1$$,
- $D_i[t_i] = t_{i+1}$ and $D_i[q_i] \rightarrow _{\Delta }^{*} q_{i+1}$ for all $i \leqslant n$, and
- $$q_{n+1} = q$$ and $$t_{n+1} = t$$

by a straightforward induction proof on *t*. Because $n > |Q|$ there exist indices $1 \leqslant i < j \leqslant n$ such that $q_i = q_j$. We construct the contexts $C_1 = D_n[\dots [D_j]\dots ]$ and $C_2 = D_{j-1}[\dots [D_i]\dots ]$. Note that $C_2 \ne \Box$ as $i < j$. We obtain $C_2[q_i] \rightarrow _{\Delta }^{*} q_j$ and $C_1[q_j] \rightarrow _{\Delta }^{*} q_{n+1}$ by induction on the difference $j - i$. By letting $p = q_i = q_j$ and $u = t_i$ we obtain the desired result. $\square$

### Infinity Predicate

Below we show that $\textsf{INF}_R$ is regular for every $\textsf{RR}_{2}$ relation *R*. The following definition originates from [11] and plays an important role in the proof.

#### Definition 5

Given a tree automaton $\mathcal {A}= (\mathcal {F}^{(2)},Q,Q_f,\Delta )$, the set $Q_\infty \subseteq Q$ consists of all states $q \in Q$ such that $\langle \bot ,t\rangle \rightarrow _{\Delta }^{*} q$ for infinitely many terms $t \in \mathcal {T}(\mathcal {F})$.

#### Example 7

Consider the binary relation

$$\begin{aligned} R = \{(\textsf{f}(\textsf{a},\textsf{g}^n(\textsf{b})),\textsf{g}^m(\textsf{f}(\textsf{a},\textsf{b}))) \mid n = 2 \text { and }m \geqslant 1 \text { or }n \geqslant 3 \text { and }m = 1\} \end{aligned}$$

over $\mathcal {T}(\mathcal {F})$ with $\mathcal {F}= \{\textsf{a},\textsf{b},\textsf{f},\textsf{g}\}$. Its encoding $\langle R\rangle$ is accepted by the automaton $\mathcal {A}= (\mathcal {F}^{(2)},Q,Q_f,\Delta )$ with $Q = \{0,\dotsc ,11\}$, $Q_f = \{0\}$, and $\Delta$ consisting of the following transition rules:For instance,

$$\begin{aligned} \langle \textsf{f}(\textsf{a},&\textsf{g}(\textsf{g}(\textsf{b}))),\textsf{g}(\textsf{f}(\textsf{a},\textsf{b})) \rangle \,=\, \textsf{fg}(\textsf{af}(\bot \textsf{a},\bot \textsf{b}), \textsf{g}\bot (\textsf{g}\bot (\textsf{b}\bot ))) \\&\,\rightarrow _{\Delta }^{*}\, \textsf{fg}(\textsf{af}(3,4),\textsf{g}\bot (\textsf{g}\bot (7))) \,\rightarrow _{\Delta }^{*}\, \textsf{fg}(1,\textsf{g}\bot (6)) \,\rightarrow _{\Delta }\, \textsf{fg}(1,2) \,\rightarrow _{\Delta }\, 0 \end{aligned}$$

but $\langle \textsf{f}(\textsf{a},\textsf{g}(\textsf{b}),\textsf{f}(\textsf{a},\textsf{b}))\rangle = \textsf{ff}(\textsf{aa},\textsf{gb}(\textsf{b}\bot ))$ is not accepted. We have $Q_\infty = \{5\}$. State 5 is reached by $\langle \bot ,\textsf{g}^n(\textsf{f}(\textsf{a},\textsf{b}))\rangle$ for all $n \geqslant 0$.

#### Definition 6

Given $\mathcal {A}= (\mathcal {F}^{(2)},Q,Q_f,\Delta )$, we define the tree automaton

$$\begin{aligned} \mathcal {A}_\infty = (\mathcal {F}^{(2)},Q \cup \bar{Q},\bar{Q}_f,\Delta \cup \bar{\Delta }) \end{aligned}$$

Here $\bar{Q}$ is a copy of *Q* where every state is dashed: $\bar{q} \in \bar{Q}$ if and only if $q \in Q$. For every transition rule $fg(q_1,\dotsc ,q_{n}) \rightarrow q \in \Delta$ we have the following transition rules in $\bar{\Delta }$:12Moreover, for every $\varepsilon$-transition $p \rightarrow q \in \Delta$ we add3to $\bar{\Delta }$. We write $\Delta '$ for $\Delta \cup \bar{\Delta }$.

Dashed states are created by rules of shape (1) and propagated by rules of shapes (2) and (3). The above construction differs from the one in [11]; instead of (1) the latter contains $fg(q_1,\dotsc ,q_{n}) \rightarrow \bar{q}$ if $q_i \in Q_\infty$ for some $i > \textsf{arity}(f)$. In an implementation, rather than adding all dashed states and all transition rules of shape (2), the necessary rules would be computed by propagating the dashes created by (1) in order to avoid the appearance of unreachable dashed states. When $\mathcal {A}_\infty$ is used in isolation, a single bit suffices to record that a dashed state occurred during a computation.

#### Example 8

For the tree automaton $\mathcal {A}$ from Example 7 we obtain $\mathcal {A}_\infty$ by adding the following transition rules (the missing rules of shape (2) involve unreachable states):The unique final state of $\mathcal {A}_\infty$ is $\bar{0}$. We have $\langle \textsf{f}(\textsf{a},\textsf{g}(\textsf{g}(\textsf{b}))),\textsf{g}(\textsf{f}(\textsf{a},\textsf{b}))\rangle \in L(\mathcal {A}_\infty )$ but there is no term *u* such that $\langle \textsf{f}(\textsf{a}(\textsf{g}(\textsf{b})),u\rangle \in L(\mathcal {A}_\infty )$.

The following preliminary lemma is used in the proof of the theorem below and provides a characterization of the ground terms that reduce to a dashed state.

#### Lemma 3

Let *t* be a term in $\mathcal {T}(\mathcal {F}^{(2)})$. If $t \rightarrow _{\!\mathcal {A}_\infty }^{*} \bar{p}$ then there exist a state $q \in Q_\infty$, a context *C*, and a term *s* such that $t = C[s]$, $\textsf{root}(s) = \bot f$ with $f \in \mathcal {F}$, $s \rightarrow _{\!\mathcal {A}_\infty }^{*} \bar{q}$, and $C[\bar{q}] \rightarrow _{\!\mathcal {A}_\infty }^{*} \bar{p}$. 

#### Proof

Write $t = gf(t_1,\dotsc ,t_{n})$. We distinguish two cases, depending on when the dash is introduced in $t \rightarrow _{\!\mathcal {A}_\infty }^{*} \bar{p}$. In the first case the dash is created by a root step:

$$\begin{aligned} t \rightarrow _{\Delta }^{*} gf(q_1,\dotsc ,q_{n}) \rightarrow _{\Delta '} \bar{q} \rightarrow _{\Delta '}^{*} \bar{p} \end{aligned}$$

We have $g = \bot$ and $q \in Q_\infty$ by (1). Hence we can take $s = t$ and $C = \square$. Note that $\textsf{root}(s) = gf = \bot f$. In the second case the dash is created during the evaluation of an argument $t_i$ of *t*, and hence the given sequence $t \rightarrow _{\!\mathcal {A}_\infty }^{*} \bar{p}$ can be rearranged as

$$\begin{aligned} t \rightarrow _{\!\mathcal {A}_\infty }^{*} gf(t_1,\dots ,\bar{r},\dots ,t_n) \rightarrow _{\!\mathcal {A}_\infty }^{*} \bar{p} \end{aligned}$$

The induction hypothesis yields a state $q \in Q_\infty$, a context $C'$, and a term *s* such that $t_i = C'[s]$, $\textsf{root}(s) = \bot f'$ with $f' \in \mathcal {F}$, $s \rightarrow _{\!\mathcal {A}_\infty }^{*} \bar{q}$, and $C'[\bar{q}] \rightarrow _{\!\mathcal {A}_\infty }^{*} \bar{r}$. In this case we simply take $C = t[C']_i = gf(t_1,\dots ,C',\dots ,t_n)$. We have $t = t[t_i]_i = t[C'[s']]_i = C[s]$ and $C[\bar{q}] = gf(t_1,\dots ,C'[\bar{q}],\dots ,t_n) \rightarrow _{\!\mathcal {A}_\infty }^{*} gf(t_1,\dots ,\bar{r},\dots ,t_n) \rightarrow _{\!\mathcal {A}_\infty }^{*} \bar{p}$. $\square$

The following result goes back to a technical report by Dauchet and Tison [11].

#### Theorem 3

($T~\mathsf {::=}~\textsf{INF}_R$)  The set $\textsf{INF}_{R}$ is regular for every $\textsf{RR}_{2}$ relation *R*. 

#### Proof

Let $\mathcal {A}= (\mathcal {F}^{(2)},Q,Q_f,\Delta )$ be a tree automaton that accepts $\langle R\rangle$. We show that $\textsf{INF}_{R} = \Pi _2(L(\mathcal {A}_\infty ))$. The regularity of $\textsf{INF}_{R}$ then follows from Theorem 2.

First suppose $t \in \textsf{INF}_{R}$. So $\langle t,u\rangle \in L(\mathcal {A})$ for infinitely many terms $u \in \mathcal {T}(\mathcal {F})$. Since the signature $\mathcal {F}$ is finite, there are only finitely many ground terms of any given height. Moreover, $\textsf{height}(\langle t,u\rangle ) = \max \,(\textsf{height}(t),\textsf{height}(u))$. Hence there must exist a term $u \in \mathcal {T}(\mathcal {F})$ with $\langle t,u\rangle \in L(\mathcal {A})$ such that $\textsf{height}(t) + |Q| + 1 < \textsf{height}(u)$. This is only possible if there are positions *p* and *q* such that $p \notin \mathcal {P}\textsf{os}(t)$, $pq \in \mathcal {P}\textsf{os}(u)$, and $|Q| < |q|$. From $\mathcal {P}\textsf{os}(\langle t,u\rangle ) = \mathcal {P}\textsf{os}(t) \cup \mathcal {P}\textsf{os}(u)$ we obtain $\langle t,u\rangle |_p = \langle \bot ,u|_p\rangle$. Since $\langle t,u\rangle \in L(\mathcal {A})$ there exist states $r \in Q$ and $q_f \in Q_f$ such that

$$\begin{aligned} \langle t,u\rangle = \langle t,u\rangle [\langle \bot ,u|_p\rangle ]_p \,\rightarrow _{\!\mathcal {A}}^{*}\, \langle t,u\rangle [r]_p \,\rightarrow _{\!\mathcal {A}}^{*}\, q_f \end{aligned}$$

where we assume without loss of generality that the final step in the subsequence $\langle \bot ,u|_p\rangle \rightarrow _{\!\mathcal {A}}^{*} r$ uses a non-$\varepsilon$-transition rule. From $|Q| < |q|$ and $pq \in \mathcal {P}\textsf{os}(u)$ we infer $|Q| < \textsf{height}(\langle \bot ,u|_p\rangle )$. Hence we can use the pumping lemma (Lemma 2) to conclude the existence of infinitely many terms $v \in \mathcal {T}(\mathcal {F})$ such that $\langle \bot ,v\rangle \rightarrow _{\!\mathcal {A}}^{*} r$. Hence $r \in Q_\infty$ by Definition 5. Since the final step in $\langle \bot ,u|_p\rangle \rightarrow _{\!\mathcal {A}}^{*} r$ uses a non-$\varepsilon$-transition rule, we obtain $\langle \bot ,u|_p\rangle \rightarrow _{\!\mathcal {A}_\infty }^{*} \bar{r}$ from the construction of $\mathcal {A}_\infty$ with a final application of a rule of shape (1). We obtain $\langle t,u\rangle [\bar{r}]_p \rightarrow _{\!\mathcal {A}_\infty }^{*} \bar{q}_f$ from $\langle t,u\rangle [r]_p \rightarrow _{\!\mathcal {A}}^{*} q_f$. Hence $\langle t,u\rangle \rightarrow _{\!\mathcal {A}_\infty }^{*} \bar{q}_f$ and since $\bar{q}_f \in \bar{Q}_f$, $\langle t,u\rangle \in L(\mathcal {A}_\infty )$ and thus $t \in \Pi _2(L(\mathcal {A}_\infty ))$.

Next suppose $t \in \Pi _2(L(\mathcal {A}_\infty ))$. So $\langle t,u\rangle \in L(\mathcal {A}_\infty )$ for some ground terms *u*. There exists a final state $\bar{q}_f \in \bar{Q}$ with $\langle t,u\rangle \rightarrow _{\!\mathcal {A}_\infty }^{*} \bar{q}_f$. Using Lemma 3, we obtain a context *C*, a term *s* with $\textsf{root}(s) = \bot f$ for some $f \in \mathcal {F}$, and a state $q \in Q_\infty$ such that $C[s] = \langle t,u\rangle$, $s \rightarrow _{\!\mathcal {A}_\infty }^{*} \bar{q}$, and $C[\bar{q}] \rightarrow _{\!\mathcal {A}_\infty }^{*} \bar{q}_f$. Let *p* be the position of the hole in *C*. From $C[s] = \langle t,u\rangle$ and $\textsf{root}(s) = \bot f$, we infer $p \in \mathcal {P}\textsf{os}(u) \setminus \mathcal {P}\textsf{os}(t)$. Since $q \in Q_\infty$ the set $\{v \in \mathcal {T}(\mathcal {F})\mid \langle \bot ,v\rangle \rightarrow _{\!\mathcal {A}}^{*} q\}$ is infinite. Hence the set $S = \{u[v]_p \in \mathcal {T}(\mathcal {F})\mid \langle \bot ,v\rangle \rightarrow _{\!\mathcal {A}}^{*} q\}$ is infinite, too. Let $u[w]_p \in S$. So $\langle \bot ,w\rangle \rightarrow _{\!\mathcal {A}}^{*} q$. We obtain $C[q] \rightarrow _{\!\mathcal {A}}^{*} q_f$ from $C[\bar{q}] \rightarrow _{\!\mathcal {A}_\infty }^{*} \bar{q}_f$ by erasing all dashes. We have $C[w] = \langle t,u[w]_p\rangle$ as $p \in \mathcal {P}\textsf{os}(u) {\setminus } \mathcal {P}\textsf{os}(t)$. It follows that $\langle t,u[w]_p\rangle \in L(\mathcal {A})$ and thus there are infinitely many terms $u'$ such that $\langle t,u'\rangle \in L(\mathcal {A})$. Since $\langle R\rangle = L(\mathcal {A})$ we conclude $t \in \textsf{INF}_{R}$ as desired. $\square$

Due to the definition of $Q_\infty$, the automaton $\mathcal {A}_\infty$ defined in Definition 6 is not executable. We present an equivalent but executable definition, which we name $Q^e_\infty$:

$$\begin{aligned} Q^e_\infty = \{q \mid p \leadsto p \text { and }p \leadsto q \text { for some state }p \in Q\} \end{aligned}$$

Here the relation $\leadsto$ is defined using the inference rules in Fig. . Intuitively, the first rule initializes the relation. Finding a cycle $p \leadsto ^+ p$ ensures the existence of infinitely many terms $\langle \bot , s\rangle$ that reduce to *p*. The other two rules are used to collapse cycles (and other non-empty sequences of $\varepsilon$-transitions) into single steps.

**Fig. 2:**
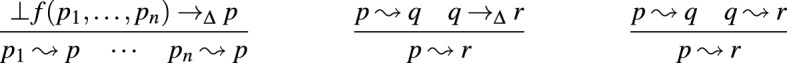
Inference rules for computing $Q^e_\infty$

Before proving that the two definitions are equivalent, we illustrate the definition of $Q^e_\infty$ by revisiting Example 7.

#### Example 9

We obtain $3 \leadsto 5$ and $4 \leadsto 5$ by applying the first inference rule to the transition rule $\bot \textsf{f}(3,4) \rightarrow 5$. Similarly, $\bot \textsf{g}(5) \rightarrow 5$ gives rise to $5 \leadsto 5$. Since $\mathcal {A}$ has no $\varepsilon$-transitions, no further inferences can be made. It follows that $Q^e_\infty = \{5\}$.

We call a term in $\mathcal {T}(\{\bot \} \times \mathcal {F})$ *right-only*. A term in $\mathcal {T}((\{\bot \} \times \mathcal {F}) \cup \{\square \})$ with exactly one occurrence of the hole $\square$ is a right-only context.

#### Definition 7

We denote the composition of $\rightarrow _{\Delta _{\lnot \varepsilon }}^{}$ and $\rightarrow _{\Delta _\varepsilon }^{*}$ by $\twoheadrightarrow _\Delta ^{}$.

The proof of the next lemma is straightforward. Note that the relations $\rightarrow _{\Delta }^{*}$ and $\twoheadrightarrow _\Delta ^{*}$ do not coincide on *mixed* terms, involving function symbols and states.

#### Lemma 4

Let *C* be a ground context. We have $C[p] \rightarrow _{\Delta }^{*} q$ if and only if $p \rightarrow _{\Delta }^{*} p'$ and $C[p'] \twoheadrightarrow _\Delta ^{*} q$ for some state $p'$. 

#### Proof

First we show $t \twoheadrightarrow _\Delta ^{*} q$ if $t \rightarrow _{\Delta }^{*} q$, for all ground terms *t* and states *q*. We use induction on $t = f(t_1,\dotsc ,t_{n})$. The given derivation $t \rightarrow _{\Delta }^{*} q$ may be written as $t \rightarrow _{\Delta }^{*} f(q_1,\dotsc ,q_{n}) \rightarrow _{\Delta _{\lnot \varepsilon }}^{} q' \rightarrow _{\Delta }^{*} q$. We obtain $t_i \twoheadrightarrow _\Delta ^{*} q_i$ for $1 \leqslant i \leqslant n$ from the induction hypothesis. Clearly, $f(q_1,\dotsc ,q_{n}) \twoheadrightarrow _\Delta ^{} q$ and hence $t \twoheadrightarrow _\Delta ^{*} q$ as desired.

Next we prove the statement of the lemma. The if direction is trivial. For the only-if direction we use induction on the ground context *C*. Let $C[p] \rightarrow _{\Delta }^{*} q$. If $C = \square$ then we take $p' = q$. Suppose $C = f(t_1,\dots ,C',\dots ,t_n)$. We may write the derivation $C[p] \rightarrow _{\Delta }^{*} q$ as $t \rightarrow _{\Delta }^{*} f(q_1,\dotsc ,q_{n}) \rightarrow _{\Delta _{\lnot \varepsilon }}^{} q' \rightarrow _{\Delta }^{*} q$. The induction hypothesis yields a state $p'$ such that $p \rightarrow _{\Delta }^{*} p'$ and $C'[p'] \twoheadrightarrow _\Delta ^{*} q_i$ and we obtain $t_j \twoheadrightarrow _\Delta ^{*} q_j$ for $j \ne i$ from the first part of the proof. We have $f(q_1,\dotsc ,q_{n}) \twoheadrightarrow _\Delta ^{} q$ and hence $C[p'] = f(t_1,\dots ,C'[p'],\dots ,t_n) \twoheadrightarrow _\Delta ^{*} q$. $\square$

#### Lemma 5

$$Q_\infty \subseteq Q^e_\infty $$ 

#### Proof

We start by proving the following claim:

4 $$\begin{aligned} \text {if }C[p] \twoheadrightarrow _\Delta ^{*} q \text { and }C \text { is a non-empty right-only context then }p \leadsto q \end{aligned}$$

We use induction on the structure of *C*. If $C = \square$ there is nothing to show. Suppose $C = \bot f(t_1,\dots ,C',\dots ,t_n)$ where $C'$ is the *i*-th subterm of *C*. The sequence $C[p] \twoheadrightarrow _\Delta ^{*} q$ can be rearranged as $C[p] = \bot f(t_1,\dots ,C'[p],\dots ,t_n) \twoheadrightarrow _\Delta ^{*} \bot f(q_1,\dotsc ,q_{n}) \rightarrow _{\Delta }^{} q' \rightarrow _{\Delta }^{*} q$. We obtain $q_i \leadsto q'$ and subsequently $q_i \leadsto q$ by using the inference rules in Fig. 2. If $C' = \Box$ then $p = q_i$ and if $C' \ne \Box$ then the induction hypothesis yields $p \leadsto q_i$ and thus $p \leadsto q$ by transitivity. This concludes the proof of (4).

Assume $q \in Q_\infty$, so there exist infinitely many terms *t* such that $\langle \bot , t\rangle \rightarrow _{\Delta }^{*} q$. Since the signature is finite, there exist terms of arbitrary height. Thus there exists an arbitrary but fixed term *t* such that the height of *t* is greater than the number of states of *Q*. Write $t = f(t_1,\dotsc ,t_{n})$. Since the height of *t* is greater than the number of the states in *Q*, there exist a subterm *s* of *t*, a state *p*, and contexts $C_1$ and $C_2 \ne \Box$ such that

1. $$\langle \bot , t\rangle = C_1[C_2[\langle \bot , s\rangle ]]$$,
2. $$\langle \bot , s\rangle \rightarrow _{\Delta }^{*} p$$,
3. $$C_2[p] \rightarrow _{\Delta }^{*} p$$, and
4. $$C_1[p] \rightarrow _{\Delta }^{*} q$$.

From Lemma 4 we obtain a state $q'$ such that $p \rightarrow _{\Delta }^{*} q'$ and $C_2[q'] \twoheadrightarrow _\Delta ^{*} p$. Hence $q' \leadsto p$ by (4). We obtain $q' \leadsto q'$ from $q' \leadsto p$ in connection with the inference rule for $\varepsilon$-transitions. We perform a case analysis of the context $C_1$.

- If $C_1 = \Box$ then $p \rightarrow _{\Delta }^{*} q$ and thus $q' \leadsto q$ follows from $q' \leadsto p$ in connection with the inference rule for $\varepsilon$-transitions. Hence $q \in Q^e_\infty$.
- If $C_1 \ne \Box$ then Lemma 4 yields a state $q''$ such that $p' \rightarrow _{\Delta }^{*} q''$ and $C_1[q''] \twoheadrightarrow _\Delta ^{*} q$. Hence $q'' \leadsto q$ by (4). We also have $C_2[q'] \twoheadrightarrow _\Delta ^{*} q''$ and thus $q' \leadsto q''$ by (4). We obtain $q' \leadsto q$ from the transitivity rule. Hence also in this case we obtain $q \in Q^e_\infty$. 

$$\square $$

For the following lemma, we need the fact that $\mathcal {A}$ can be assumed to be trim, so every state is productive and reachable. We may do so because Theorem 3 talks about regular relations, and any automaton that accepts the same language as $\mathcal {A}$ will witness the fact that the given relation *R* is regular.

#### Lemma 6

$Q^e_\infty \subseteq Q_\infty$, provided that $\mathcal {A}$ is trim. 

#### Proof

In connection with the fact that $\mathcal {A}$ accepts $R \subseteq \mathcal {T}(\mathcal {F})\times \mathcal {T}(\mathcal {F})$, trimness of $\mathcal {A}$ entails that any run $t \rightarrow _{\Delta }^{*} q$ is embedded into an accepting run $C[t] \rightarrow _{\Delta }^{*} C[q] \rightarrow _{\Delta }^{*} q_f \in Q_f$. So $C[t] = \langle u, v\rangle$ for some $(u, v) \in R$, and hence *t* must be a well-formed term. Moreover, if $\textsf{root}(t) = {\bot f}$ for some $f \in \mathcal {F}$ then $t = \langle \bot , u\rangle$ for some term $u \in \mathcal {T}(\mathcal {F})$. We now show the converse of claim (4) in the proof of Lemma 5 for the relation $\rightarrow _{\Delta }^{*}$:

5 $$\begin{aligned} \text {if }p \leadsto q \text { then }C[p] \rightarrow _{\Delta }^{*} q \text { for some ground right-only context }C \ne \Box \end{aligned}$$

We prove the claim by induction on the derivation of $p \leadsto q$. First suppose $p \leadsto q$ is derived from the transition rule $\bot f(p_1,\dots ,p_i,\dots ,p_n) \rightarrow q$ in $\Delta$ with $p_i = p$. Because all states are reachable by well-formed terms, there exist terms $t_1,\dotsc ,t_{n} \in \mathcal {T}(\mathcal {F})$ such that $\langle \bot ,t\rangle \rightarrow _{\Delta }^{*} p_i$ for all $1 \leqslant i \leqslant n$. Let $C_1 = \bot f(\langle \bot ,t_1\rangle , \dots ,\Box ,\dots ,\langle \bot ,t_n\rangle )$ where the hole is the *i*-th argument. We have $C_1[p] \rightarrow _{\Delta }^{*} \bot f(p_1,\dots ,p_i,\dots ,p_n) \rightarrow _{\Delta }^{} q$. Next suppose $p \leadsto q$ is derived from $p \leadsto q'$ and $q' \rightarrow _{\Delta }q$. The induction hypothesis yields a ground right-only context $C \ne \Box$ such that $C[p] \rightarrow _{\Delta }^{*} q'$. Hence also $C[p] \rightarrow _{\Delta }^{*} q$. Finally, suppose $p \leadsto q$ is derived from $p \leadsto r$ and $r \leadsto q$. The induction hypothesis yields non-empty ground right-only contexts $C_1$ and $C_2$ such that $C_1[p] \rightarrow _{\Delta }^{*} r$ and $C_2[r] \rightarrow _{\Delta }^{*} q$. Hence $C[p] \rightarrow _{\Delta }^{*} q$ for the context $C = C_2[C_1]$. This concludes the proof of (5).

Now let $q \in Q^e_\infty$. So there exists a state *p* such that $p \leadsto p$ and $p \leadsto q$. Using (5), we obtain non-empty ground right-only contexts $C_1$ and $C_2$ such that $C_1[p] \rightarrow _{\Delta }^{*} p$ and $C_2[p] \rightarrow _{\Delta }^{*} q$. Since all states are reachable, there exists a ground term $t \in \mathcal {T}(\mathcal {F}^{(2)})$ such that $t \rightarrow _{\Delta }^{*} p$. Hence $C_2[t] \rightarrow _{\Delta }^{*} q$ and, by the observation made at the beginning of the proof, $C_2[t]$ is a well-formed term. Since $C_2$ is right-only, it follows that $t = \langle \bot , u\rangle$ for some term $u \in \mathcal {T}(\mathcal {F})$. Now consider the infinitely many terms $t_n = C_2[C_1^n[t]]$ for $n \geqslant 0$. We have $t_n \rightarrow _{\Delta }^{*} q$ and $t_n$ is right-only by construction. Hence $q \in Q_\infty$. $\square$

#### Corollary 1

If $\mathcal {A}$ is trim then $Q^e_\infty = Q_\infty$. $\square$

### Anchored GTT Relations

Next we turn our attention to formalized constructions on (anchored) GTTs. Many of the results and automata constructions in this subsection are known. In the formalization we also employ an equivalent but more flexible definition of anchored GTT.

#### Definition 8

A *pair automaton* is a triple $\mathcal {P}= (Q,\mathcal {A},\mathcal {B})$ where $\mathcal {A}$, $\mathcal {B}$ are tree automata and $Q \subseteq Q_A \times Q_B$. We define $L(\mathcal {P}) = \{(s,t) \mid s \rightarrow _{\!\mathcal {A}}^{*} p \text { and }t \rightarrow _{\mathcal {B}}^{*} q \text { with }(p,q) \in Q\}$.

#### Lemma 7

Anchored GTTs and pair automata are equivalent. 

#### Proof

If $\mathcal {G}= (\mathcal {A},\mathcal {B})$ is a GTT then $L_a(\mathcal {G}) = L(\mathcal {P})$ for the pair automaton $\mathcal {P}= (Q,\mathcal {A},\mathcal {B})$ with $Q = \{(p,p) \mid p \in Q_A \cap Q_B\}$. Conversely, given a pair automaton $\mathcal {P}= (Q,\mathcal {A},\mathcal {B})$, we first rename the states of $\mathcal {B}$ to obtain an equivalent tree automaton $\mathcal {B}'$ such that $\mathcal {A}$ and $\mathcal {B}'$ do not share states. We add an $\varepsilon$-transition $p \rightarrow q'$ to $\mathcal {A}$ for every $(p,q) \in Q$, resulting in the tree automaton $\mathcal {A}'$. Here $q'$ is the (renamed) state in $\mathcal {B}'$ that corresponds to state *q* in $\mathcal {B}$. The GTT $\mathcal {G}= (\mathcal {A}',\mathcal {B}')$ satisfies $L_a(\mathcal {G}) = L(\mathcal {P})$. $\square$

The above lemma will be used in the sequel without mention.

#### Lemma 8

($A~\mathsf {::=}~T \times T$)  If *T* and *U* are regular sets of ground terms then $T \times U$ is an anchored GTT relation.

#### Proof

Let $A = (\mathcal {F},Q_A,Q_{fA},\Delta _A)$ and $B = (\mathcal {F},Q_B,Q_{fB},\Delta _B)$ be tree automata that accept *T* and *U*. The set $T \times U$ is accepted by the pair automaton $\mathcal {P}= (Q,\mathcal {A},\mathcal {B})$ with $Q = Q_{fA} \times Q_{fB}$. $\square$

There are several ways to associate a GTT $\mathcal {G}= (\mathcal {A},\mathcal {B})$ with a linear variable-separated TRS $\mathcal {R}$. The one in [9] uses for each rewrite rule $\ell \rightarrow r$ of $\mathcal {R}$ a unique interface state *i*, common to $\mathcal {A}$ and $\mathcal {B}$, and transition rules and states specific to $\mathcal {A}$ ($\mathcal {B}$) that accept all ground instances of $\ell$ (*r*) in state *i*. No states are shared between different rewrite rules. The resulting GTT accepts  and $\rightarrow _{\varepsilon }$ when viewed as an anchored GTT. The second way to associate a GTT with a linear variable-separated TRS $\mathcal {R}$ originates from Dauchet et al. [12]. The resulting GTT accepts a relation in between  and $\rightarrow ^{*}$. The construction that we formalized can be seen as a pair automaton version of the construction in [9].

#### Theorem 4

[$A~\mathsf {::=}~{\rightarrow _{\varepsilon }}$] The relation $\rightarrow _{\varepsilon }$ is an anchored GTT relation for every linear variable-separated TRS $\mathcal {R}$. 

#### Proof

Let $\mathcal {R}$ be a linear variable-separated TRS over a signature $\mathcal {F}$. We denote the set of left-hand (right-hand) sides of the rules in $\mathcal {R}$ by $\textsf{lhs}(\mathcal {R})$ ($\textsf{rhs}(\mathcal {R})$). Given a set of terms *T*, we write $s \mathrel {{\trianglelefteq }}T$ if *s* is a subterm of some term in *T*. Given a term *s* we write $\hat{s}$ for the ground term obtained from *s* by replacing each variable with a designated (fresh) constant $*$. Let *Q* be the set of states $\langle \hat{t}\rangle$ for each $t \mathrel {{\trianglelefteq }}\textsf{lhs}(\mathcal {R}) \cup \textsf{rhs}(\mathcal {R})$. The set $\Delta _\textsf{lhs}$ consists of the transitionsfor every $f(t_1,\dotsc ,t_{n}) \mathrel {{\trianglelefteq }}\textsf{lhs}(\mathcal {R})$ and, if some term in $\textsf{lhs}(\mathcal {R})$ contains a variable,

$$\begin{aligned} f(\langle *\rangle ,\dotsc ,\langle *\rangle )&\,\rightarrow \, \langle *\rangle \end{aligned}$$

for every $f \in \mathcal {F}$. The set $\Delta _\textsf{rhs}$ is defined similarly, using $\textsf{rhs}(\mathcal {R})$ instead of $\textsf{lhs}(\mathcal {R})$ for generating the rules. We now define $\mathcal {P}_\mathcal {R}= (Q,\Delta _\textsf{lhs},\Delta _\textsf{rhs})$ with $Q = \{(\langle \hat{\ell }\rangle ,\langle \hat{r}\rangle ) \mid \ell \rightarrow r \in \mathcal {R}\}$. It is easy to prove that $L_a(\mathcal {P}_\mathcal {R}) = {\rightarrow _{\varepsilon }}$. $\square$

The other binary relations associated with a TRS $\mathcal {R}$ (like  and $\leftrightarrow _{\mathcal {R}}^{*}$) will be obtained from the root-step relation $\rightarrow _{\varepsilon }$ by automata constructions that operate on anchored GTT relations and $\textsf{RR}_{2}$ relations.

#### Example 10

The pair automaton $\mathcal {P}_\mathcal {R}= (Q,\mathcal {A},\mathcal {B})$ constructed in the above proof consists of the transition rulesand accepts the root-step relation $\rightarrow _{\varepsilon }$ of our leading TRS $\mathcal {R}$. The state pairs in *Q* are presented as $\varepsilon$-transitions and perform the transfer from left-hand sides to right-hand sides of $\mathcal {R}$. For instance, $\textsf{g}(\textsf{a},\textsf{f}(\textsf{f}(\textsf{b}))) \rightarrow _{\varepsilon } \textsf{f}(\textsf{a})$ is witnessed by . To shorten the notation in subsequent examples, we number the states as follows:

$$\begin{aligned} 0&= \langle *\rangle&1&= \langle \textsf{a}\rangle&2&= \langle \mathsf {f(a)}\rangle&3&= \langle {\textsf{g}(\textsf{a},*)\rangle }&4&= \langle \textsf{b}\rangle&\end{aligned}$$

Hence the transition rules are presented as follows:To turn $\mathcal {P}_\mathcal {R}$ into an equivalent anchored GTT $\mathcal {G}_\mathcal {R}= (\mathcal {A}',\mathcal {B}')$ we rename states 1 and 2 in $\mathcal {B}$ into 5 and 6 and add the pairs in *Q* as $\varepsilon$-transitions to $\mathcal {A}$, after applying the renaming to their targets:

$$\begin{aligned} \Delta _A':{} & {} \textsf{a}&\rightarrow 0&\textsf{b}&\rightarrow 0&\textsf{f}(0)&\rightarrow 0&\textsf{g}(0,0)&\rightarrow 0 \\{} & {} \textsf{a}&\rightarrow 1{} & {} {}&\textsf{f}(1)&\rightarrow 2&\textsf{g}(1,0)&\rightarrow 3 \\{} & {} 1&\rightarrow 4&2&\rightarrow 4&3&\rightarrow 6 \\ \Delta _B:{} & {} \textsf{a}&\rightarrow 5&\textsf{b}&\rightarrow 4&\textsf{f}(5)&\rightarrow 6 \end{aligned}$$

Next we turn to composition and transitive closure.

#### Definition 9

Given tree automata $\mathcal {A}$ and $\mathcal {B}$, $\Delta _\varepsilon (\mathcal {A},\mathcal {B})$ is the set of $\varepsilon$-transitions $\leadsto$ defined by the inference rules in Fig. .

**Fig. 3:**
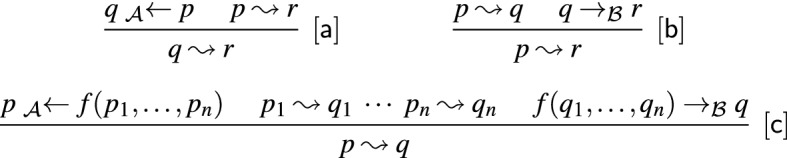
$$\Delta _\varepsilon (\mathcal {A},\mathcal {B})$$

The inference rule $[\textsf{c}]$ appeared first in [17]. Since there are only finitely many $\varepsilon$-transitions between states in *Q*, $\Delta _\varepsilon (\mathcal {A},\mathcal {B})$ can be effectively computed. The next result provides a useful equivalent characterization (which is presented as a definition in [8, 12]).

#### Example 11

For the (anchored) GTT $\mathcal {G}_\mathcal {R}$ of Example 10, which will be referred to as $\mathcal {G}= (\mathcal {A},\mathcal {B})$ in the following, the set $\Delta _\varepsilon (\mathcal {A},\mathcal {B})$ consists of the following seven $\varepsilon$-transitions:Since $\mathcal {B}$ does not contain $\varepsilon$-transitions, the inference rule $[\textsf{b}]$ is not used here.

#### Lemma 9

If $\mathcal {A}$ and $\mathcal {B}$ are tree automata over a signature $\mathcal {F}$ then 
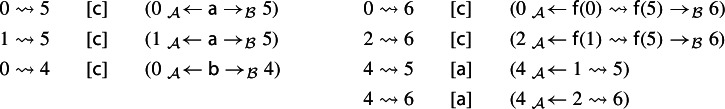


#### Proof

First suppose there exists a ground term $t \in \mathcal {T}(\mathcal {F})$ with  for states *p* of $\mathcal {A}$ and *q* of $\mathcal {B}$. We show $p \leadsto q$ by induction on $t = f(t_1,\dotsc ,t_{n})$. The sequence $t \rightarrow _{\!\mathcal {A}}^{*} p$ can be written as $t \rightarrow _{\!\mathcal {A}}^{*} f(p_1,\dotsc ,p_{n}) \rightarrow _{\!\mathcal {A}}^{} p' \rightarrow _{\!\mathcal {A}}^{*} p$ with states $p_1,\dotsc ,p_{n},p'$ of $\mathcal {A}$. Similarly, $t \rightarrow _{\mathcal {B}}^{*} f(q_1,\dotsc ,q_{n}) \rightarrow _{\mathcal {B}}^{} q' \rightarrow _{\mathcal {B}}^{*} q$ with states $q_1,\dotsc ,q_{n}, q'$ of $\mathcal {B}$. We have  and thus $p_i \leadsto q_i$ by the induction hypothesis, for $1 \leqslant i \leqslant n$. Hence we obtain $p' \leadsto q'$ by $[\textsf{c}]$. Repeated applications of the inference rules $[\textsf{a}]$ and $[\textsf{b}]$ in connection with $p' \rightarrow _{\!\mathcal {A}}^{*} p$ and $q' \rightarrow _{\mathcal {B}}^{*} q$ yields $p \leadsto q$. Hence $p \leadsto q \in \Delta _\varepsilon (\mathcal {A},\mathcal {B})$ as desired.

Next suppose $p \leadsto q \in \Delta _\varepsilon (\mathcal {A},\mathcal {B})$. We show the existence of a ground term $t \in \mathcal {T}(\mathcal {F})$ such that  by induction on the derivation of $p \leadsto q$. In the base case $[\textsf{c}]$ is used with  and $a \rightarrow _{\mathcal {B}}^{} q$ for some constant *a* and hence we can take $t = a$. For the induction step we consider three cases, depending on which inference rule is used to derive $p \leadsto q$. First suppose $[\textsf{c}]$ is used. So there exist transition rules $f(p_1,\dotsc ,p_{n}) \rightarrow p$ in $\mathcal {A}$ and $f(q_1,\dotsc ,q_{n}) \rightarrow q$ in $\mathcal {B}$ such that $p_i \leadsto q_i$ for $1 \leqslant i \leqslant n$. The induction hypothesis yields ground terms $t_1,\dotsc ,t_{n}$ such that  for $1 \leqslant i \leqslant n$. Hence  for $t = f(t_1,\dotsc ,t_{n})$. Next suppose $[\textsf{a}]$ is applied to derive $p \leadsto q$. So there exists a state $p'$ such that . The induction hypothesis yields a ground term $t \in \mathcal {T}(\mathcal {F})$ such that  and hence also . The reasoning for $[\textsf{b}]$ is the same. $\square$

#### Theorem 5

($A~\mathsf {::=}~A \mathrel {\circ } A$)  Anchored GTT relations are effectively closed under composition. 

#### Proof

Let $\mathcal {P}_1 = (Q_1,\mathcal {A}_1,\mathcal {B}_1)$ and $\mathcal {P}_2 = (Q_2,\mathcal {A}_2,\mathcal {B}_2)$ be pair automata (operating on terms over the same signature). We construct the pair automaton $\mathcal {P}= (Q,\mathcal {A}_1,\mathcal {B}_2)$ with

$$\begin{aligned} Q ~=~ Q_1 \mathrel {\circ } \Delta _\varepsilon (\mathcal {B}_1,\mathcal {A}_2) \mathrel {\circ } Q_2 \end{aligned}$$

We claim that $L(\mathcal {P}) = L(\mathcal {P}_1) \mathrel {\circ } L(\mathcal {P}_2)$. First let $(s,t) \in L(\mathcal {P})$. We have $s \rightarrow _{\mathcal {A}_1}^{*} p$ and $t \rightarrow _{\mathcal {B}_2}^{*} q$ for some $(p,q) \in Q$. The definition of $Q_2$ yields states $p'$ and $q'$ such that $(p,p') \in Q_1$, $(p',q') \in \Delta _\varepsilon (\mathcal {B}_1,\mathcal {A}_2)$, and $(q',q) \in Q_2$. According to Lemma 9 there exists a ground term *u* such that $u \rightarrow _{\mathcal {B}_1}^{*} p'$ and $u \rightarrow _{\mathcal {A}_2}^{*} q'$. Hence $(s,u) \in L(\mathcal {P}_1)$ and $(u,t) \in L(\mathcal {P}_2)$ and thus $(s,t) \in L(\mathcal {P}_1) \mathrel {\circ } L(\mathcal {P}_2)$.

For the converse, let $(s,t) \in L(\mathcal {P}_1) \mathrel {\circ } L(\mathcal {P}_2)$. So there exists a ground term *u* such that $(s,u) \in L(\mathcal {P}_1)$ and $(u,t) \in L(\mathcal {P}_2)$. Hence there are pairs $(p_1,q_1) \in Q_1$ and $(p_2,q_2) \in Q_2$ such that $s \rightarrow _{\mathcal {A}_1}^{*} p_1$, $u \rightarrow _{\mathcal {B}_1}^{*} q_1$, $u \rightarrow _{\mathcal {A}_2}^{*} p_2$, and $t \rightarrow _{\mathcal {B}_2}^{*} q_2$. Lemma 9 yields $(q_1,p_2) \in \Delta _\varepsilon (\mathcal {B}_1,\mathcal {A}_2)$. Hence $(p_1,q_2) \in Q$ and thus $(s,t) \in L(\mathcal {P})$. $\square$

#### Example 12

We compose the pair automaton $\mathcal {P}_\mathcal {R}= (Q,\mathcal {A},\mathcal {B})$ of Example 10 with itself. We have $\Delta _\varepsilon (\mathcal {B},\mathcal {A}) = \Delta _\varepsilon (\mathcal {A},\mathcal {B})^- = \{(1,0),(1,1),(4,0),(2,2),(2,0)\}$. Hence we obtain the pair automaton $\mathcal {P}' = (Q',\mathcal {A},\mathcal {B})$ with $Q' = Q \mathrel {\circ } \Delta _\varepsilon (\mathcal {B},\mathcal {A}) \mathrel {\circ } Q = \{(3,4)\}$. We have $L(\mathcal {A},3) = \{\textsf{g}(\textsf{a},t) \mid t \in \mathcal {T}(\mathcal {F})\}$ and $L(\mathcal {B},4) = \{\textsf{b}\}$. Hence, we obtain $L(\mathcal {P}') = L(\mathcal {A},3) \times L(\mathcal {B},4) = {\rightarrow _{\varepsilon }^{2}}$ as expected.

**Fig. 4:**
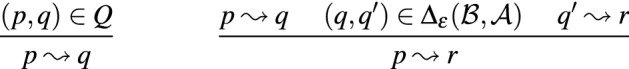
$$\Delta _+(\mathcal {P})$$ for $$\mathcal {P}= (Q,\mathcal {A},\mathcal {B})$$

#### Theorem 6

($A~\mathsf {::=}~A^+$)  Anchored GTT relations are effectively closed under transitive closure. 

#### Proof

Let $\mathcal {P}= (Q,\mathcal {A},\mathcal {B})$ be a pair automaton. We construct the pair automaton $\mathcal {P}_+ = (\Delta _+(\mathcal {P}),\mathcal {A},\mathcal {B})$ where $\Delta _+(\mathcal {P})$ is the binary relation on states defined by the inference rules in Fig. . We claim that $L(\mathcal {P}_+) = L(\mathcal {P})^+$. From the first inference rule we immediately obtain $L(\mathcal {P}) \subseteq L(\mathcal {P}_+)$. The second inference rule, together with the definition of *Q* in the proof of Theorem 5, yields $L(\mathcal {P}_+) \mathrel {\circ } L(\mathcal {P}_+) \subseteq L(\mathcal {P}_+)$. Hence $L(\mathcal {P})^+ \subseteq L(\mathcal {P}_+)$.

For the converse, let $(s,t) \in L(\mathcal {P}_+)$. So there exists a pair $p \leadsto q$ such that $s \rightarrow _{\mathcal {A}}^{*} p$ and $t \rightarrow _{\mathcal {B}}^{*} q$. We prove $(s,t) \in L(\mathcal {P})^+$ by induction on the derivation of $p \leadsto q$. If $(p,q) \in Q$ then $(s,t) \in L(\mathcal {P})$. Suppose $p \leadsto p'$, $(p',q') \in \Delta _\varepsilon (\mathcal {B},\mathcal {A})$, and $q' \leadsto q$. According to Lemma 9 there exists a ground term *u* such that $u \rightarrow _{\mathcal {B}}^{*} p'$ and $u \rightarrow _{\!\mathcal {A}}^{*} q'$. The induction hypothesis yields $(s,u) \in L(\mathcal {P})^+$ and $(u,t) \in L(\mathcal {P})^+$. Hence also $(s,t) \in L(\mathcal {P})^+$. $\square$

#### Example 13

Consider the pair automaton $\mathcal {P}_\mathcal {R}= (Q,\mathcal {A},\mathcal {B})$ of Example 10. As observed in Example 12, $\Delta _\varepsilon (\mathcal {B},\mathcal {A}) = \{(1,0),(1,1),(4,0),(2,2),(2,0)\}$. Hence we obtain the pair automaton $\mathcal {P}_+ = (\Delta _+(\mathcal {P}),\mathcal {A},\mathcal {B})$ with $\Delta _+(\mathcal {P}) = \{(1,4),(2,4),(3,2),(3,4)\}$. The pair (3, 4) is obtained from the second inference rules with $p = 3$, $q = q' = 2$ and $r = 4$. We have $\textsf{g}(\textsf{a},\textsf{b}) \rightarrow _{\varepsilon } \textsf{f}(\textsf{a}) \rightarrow _{\varepsilon } \textsf{b}$ and the pair $(\textsf{g}(\textsf{a},\textsf{b}),\textsf{b})$ is accepted by $\mathcal {P}_+$ as $\textsf{g}(\textsf{a},\textsf{b}) \rightarrow _{\!\mathcal {A}}^{*} 3$ and $\textsf{b} \rightarrow _{\mathcal {B}}4$ with $(3,4) \in \Delta _+(\mathcal {P})$. Furthermore, $\textsf{g}(\textsf{a},\textsf{b}) \rightarrow _{\varepsilon } \textsf{f}(\textsf{a}) \rightarrow _{}\textsf{f}(\textsf{b})$ but $\textsf{g}(\textsf{a},\textsf{b}) \rightarrow _{\varepsilon }^{+} \textsf{f}(\textsf{b})$ does not hold, and one readily checks that the pair $(\textsf{g}(\textsf{a},\textsf{b}),\textsf{f}(\textsf{b}))$ is not accepted by $\mathcal {P}_+$.

Two further closure operations on anchored GTT relations are inverse and union. Recall that GTT relations are not closed under union.

#### Lemma 10

($A~\mathsf {::=}~A^- \mid A \cup A$)  Anchored GTT relations are effectively closed under inverse and union. 

#### Proof

Given a pair automaton $\mathcal {P}= (Q,\mathcal {A},\mathcal {B})$, we have $L(\mathcal {P})^- = L(\mathcal {P}^-)$ for the pair automaton $\mathcal {P}^- = (Q^-,\mathcal {B},\mathcal {A})$. Here $Q^- = \{(q,p) \mid (p,q) \in Q\}$. Given pair automata $\mathcal {P}_1 =(Q_1,\mathcal {A}_1,\mathcal {B}_1)$ and $\mathcal {P}_2 = (Q_2,\mathcal {A}_2,\mathcal {B}_2)$ without common states, $L(\mathcal {P}_1) \cup L(\mathcal {P}_2) = L(\mathcal {P})$ for the pair automaton $\mathcal {P}= (Q_1 \cup Q_2,\mathcal {A}_1 \cup \mathcal {A}_2,\mathcal {B}_1 \cup \mathcal {B}_2)$. $\square$

Next we present a modified composition operation  that preserves anchored GTT relations.

#### Definition 10

Given two binary relations ${\bowtie }_1$ and ${\bowtie }_2$ on the same set of ground terms, their modified composition  is defined as the relation

We have . The proof that anchored GTT relations are closed under  requires a preliminary result on the interplay between GTTs and anchored GTTs.

#### Lemma 11

The composition of an anchored GTT relation and a GTT relation is an anchored GTT relation.

#### Proof

Let $\mathcal {P}= (Q,\mathcal {A}_1,\mathcal {B}_1)$ be a pair automaton and $\mathcal {G}= (\mathcal {A}_2,\mathcal {B}_2)$ a GTT. Without loss of generality we assume that $\mathcal {P}$ and $\mathcal {G}$ do not share states. Define the pair automaton

$$\begin{aligned} \mathcal {P}' = (Q,\mathcal {A}_1,\mathcal {B}_1 \cup \Delta _\varepsilon (\mathcal {A}_2,\mathcal {B}_1) \cup \mathcal {B}_2) \end{aligned}$$

We claim that $L(\mathcal {P}') = L(\mathcal {P}) \mathrel {\circ } L(\mathcal {G})$. First let $(s,t) \in L(\mathcal {P}')$. So $s \rightarrow _{\mathcal {A}_1}^{*} p$ and $t \rightarrow _{\mathcal {B}'}^{*} q$ with $(p,q) \in Q$ and $\mathcal {B}'$ abbreviating $\mathcal {B}_1 \cup \Delta _\varepsilon (\mathcal {A}_2,\mathcal {B}_1) \cup \mathcal {B}_2$. Because $\mathcal {P}$ and $\mathcal {G}$ do not share states, the sequence $t \rightarrow _{\mathcal {B}'}^{*} q$ can be rearranged as follows:

$$\begin{aligned} t = C[t_1,\dotsc ,t_{n}]&\rightarrow _{\mathcal {B}_2}^{*} C[q_1,\dotsc ,q_{n}] \rightarrow _{\Delta _\varepsilon (\mathcal {A}_2,\mathcal {B}_1)}^{*} C[r_1,\dotsc ,r_{n}] \rightarrow _{\mathcal {B}_1}^{*} q \end{aligned}$$

Here *C* is a multi-hole context with $n \geqslant 0$ holes. Using Lemma 9 we obtain ground terms $u_1,\dotsc ,u_{n}$ such that $u_i \rightarrow _{\mathcal {A}_2}^{*} q_i$ and $u \rightarrow _{\mathcal {B}_1}^{*} r_i$ for all $1 \leqslant i \leqslant n$. Define the term $u = C[u_1,\dotsc ,u_{n}]$. We have $u \rightarrow _{\mathcal {B}_1}^{*} C[r_1,\dotsc ,r_{n}] \rightarrow _{\mathcal {B}_1}^{*} q$ and thus $(s,u) \in L(\mathcal {P})$. Furthermore, $u \rightarrow _{\mathcal {A}_2}^{*} C[q_1,\dotsc ,q_{n}]$ and thus also $(u,t) \in L(\mathcal {G})$. Hence $(s,t) \in L(\mathcal {P}) \mathrel {\circ } L(\mathcal {G})$.

For the converse direction, let $(s,t) \in L(\mathcal {P})$ and $(t,u) \in L(\mathcal {G})$. So $s \rightarrow _{\mathcal {A}_1}^{*} p$ and $t \rightarrow _{\mathcal {B}_1}^{*} q$ with $(p,q) \in Q$. Moreover, there exists a multi-hole context *C* with $n \geqslant 0$ holes, terms $t_1,\dotsc ,t_{n}, u_1,\dotsc ,u_{n}$, and states $r_1,\dotsc ,r_{n}$ such that $t = C[t_1,\dotsc ,t_{n}]$, $u = C[u_1,\dotsc ,u_{n}]$, and $t_i \rightarrow _{\mathcal {A}_2}^{*} r_i$ and $u_i \rightarrow _{\mathcal {B}_2}^{*} r_i$ for all $1 \leqslant i \leqslant n$. The sequence $t \rightarrow _{\mathcal {B}_1}^{*} q$ can be written as $t = C[t_1,\dotsc ,t_{n}] \rightarrow _{\mathcal {B}_1}^{*} C[q_1,\dotsc ,q_{n}] \rightarrow _{\mathcal {B}_1}^{*} q$ for some states $q_1,\dotsc ,q_{n}$. By Lemma 9, $r_i \rightarrow q_i$ is a transition rule in $\Delta _\varepsilon (\mathcal {A}_2,\mathcal {B}_1)$. Hence $u = C[u_1,\dotsc ,u_{n}] \rightarrow _{\mathcal {B}_2}^{*} C[r_1,\dotsc ,r_{n}] \rightarrow _{\Delta _\varepsilon (\mathcal {A}_2,\mathcal {B}_1)}^{*} C[q_1,\dotsc ,q_{n}] \rightarrow _{\mathcal {B}_1}^{*} q$ and thus $(s,u) \in L(\mathcal {P}')$ as desired. $\square$

#### Example 14

We consider the pair automaton $\mathcal {P}_\mathcal {R}$ and the GTT $\mathcal {G}_\mathcal {R}$ of Example 10. The construction in the above proof requires that $\mathcal {P}_\mathcal {R}$ and $\mathcal {G}_\mathcal {R}$ do not share states, so we rename the states of $\mathcal {G}_\mathcal {R}$ (by adding a prime). We obtain the pair automaton $\mathcal {P}' = (\{(1,4),(2,4),(3,2)\},\mathcal {A}',\mathcal {B}')$ with

$$\begin{aligned} {\mathcal {A}'}:{} & {} \textsf{a}&\rightarrow 0&\textsf{b}&\rightarrow 0&\textsf{f}(0)&\rightarrow 0&\textsf{g}(0,0)&\rightarrow 0 \\{} & {} \textsf{a}&\rightarrow 1{} & {} {}&\textsf{f}(1)&\rightarrow 2&\textsf{g}(1,0)&\rightarrow 3 \\ \mathcal {B}':{} & {} \textsf{a}&\rightarrow 1&\textsf{b}&\rightarrow 4&\textsf{f}(1)&\rightarrow 2&0'&\rightarrow 1&1'&\rightarrow 1 \\{} & {} \textsf{a}&\rightarrow 5'&\textsf{b}&\rightarrow 4'&\textsf{f}(5')&\rightarrow 6'&0'&\rightarrow 4&0'&\rightarrow 2 \\{} & {} 2'&\rightarrow 2&4'&\rightarrow 1&4'&\rightarrow 2 \end{aligned}$$

We can also trim the resulting pair automata by trimming the underlying automata $\mathcal {A}'$ and $\mathcal {B}'$. We declare a state *q* of $\mathcal {A}'$ to be productive if $C[q] \rightarrow _{\mathcal {A}'}^{*} r$ for some context *C* and state $r \in \{p \mid (p,p') \in Q\}$. For the automaton $\mathcal {B}'$ we use the second components $\{p' \mid (p,p') \in Q\}$. In our case $\mathcal {A}'$ is already trim, but $\mathcal {B}'$ simplifies to

$$\begin{aligned} \textsf{a}&\rightarrow 1&\textsf{b}&\rightarrow 4&\textsf{f}(1)&\rightarrow 2&\textsf{b}&\rightarrow 4'&4'&\rightarrow 1&4'&\rightarrow 2 \end{aligned}$$

We have $L(\mathcal {P}') = \{(\textsf{f}(\textsf{a}),\textsf{b}),(\textsf{a},\textsf{b})\} \cup \{\textsf{g}(\textsf{a},t) \mid t \in \mathcal {T}(\mathcal {F})\} \times \{\textsf{b},\textsf{f}(\textsf{a}),\textsf{f}(\textsf{b})\}$, which indeed coincides with the relation  induced by our leading TRS $\mathcal {R}$.

#### Theorem 7

()  Anchored GTT relations are effectively closed under modified composition. 

#### Proof

The construction $L(\mathcal {P}) \times L(\mathcal {G}) \mapsto L(\mathcal {P}')$ in the proof of Lemma 11 and its symmetric counterpart $L(\mathcal {G}) \times L(\mathcal {P}) \mapsto L(\mathcal {P}')$ in connection with Lemma 10 ensure that  is an anchored GTT relation. $\square$

In Theorem 6 we have seen that anchored GTT relations are closed under transitive closure. GTT relations are also closed under transitive closure, which is the reason they were developed in the first place, but the construction is different from the one for anchored GTT relations and the correctness proof is considerably more involved. We present this construction as a modified transitive closure operation that preserves anchored GTT relations.

#### Definition 11

The modified transitive closure  of a binary relation ${\bowtie }$ on ground terms is defined as the relation

We have . The proof that anchored GTT relations are effectively closed under  employs the set $\Delta _+(\mathcal {A},\mathcal {B})$ consisting of $\varepsilon$-transitions $p \leadsto q$ that are computed by the inference rules in Fig. .

**Fig. 5:**
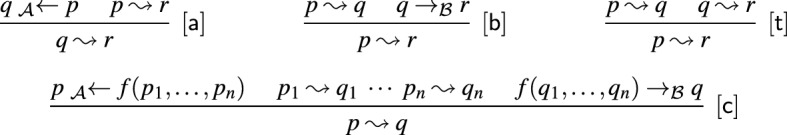
$$\Delta _+(\mathcal {A},\mathcal {B})$$

#### Definition 12

Given a GTT $\mathcal {G}= (\mathcal {A},\mathcal {B})$, we write $A_+$ for $\mathcal {A}\cup \Delta _+(\mathcal {B},\mathcal {A})$ and $B_+$ for $\mathcal {B}\cup \Delta _+(\mathcal {A},\mathcal {B})$. The GTT $\mathcal {G}_+$ is defined as $(\mathcal {A}_+,\mathcal {B}_+)$.

According to the following lemma, the multi-hole context closure of an anchored GTT relation is a GTT relation using the same GTT.

#### Lemma 12

For every GTT $\mathcal {G}$, $L(\mathcal {G}) = L_a(\mathcal {G})^{\geqslant }_{\geqslant }$. 

#### Proof

Let $\mathcal {G}= (\mathcal {A},\mathcal {B})$. If $(s,t) \in L(\mathcal {G})$ then there exist a context *C* with $n \geqslant 0$ holes, terms $s_1,\dotsc ,s_{n}, t_1,\dotsc ,t_{n}$, and states $q_1,\dotsc ,q_{n}$ with $s = C[s_1,\dotsc ,s_{n}]$, $t = C[t_1,\dotsc ,t_{n}]$, and  for all $1 \leqslant i \leqslant n$. We have $(s_i,t_i) \in L_a(\mathcal {G})$ for all $1 \leqslant i \leqslant n$ by definition of anchored GTTs. Moreover, $C \in \mathcal {C}_\geqslant \cap \mathcal {C}^\geqslant$. Hence $(s,t) \in L_a(\mathcal {G})^{\geqslant }_{\geqslant }$. The converse is equally easy. $\square$

#### Lemma 13

Let $\mathcal {G}= (\mathcal {A},\mathcal {B})$ be a GTT. If $(p,q) \in \Delta _+(\mathcal {A},\mathcal {B})$ then $(s,t) \in L(\mathcal {G}^-)^+$ for some ground terms $s \in L(\mathcal {A},p)$ and $t \in L(\mathcal {B},q)$. 

#### Proof

We use induction on the relation $\leadsto$ defined by the inference rules in Fig. 5. In the base case $[\textsf{c}]$ is used with  and $a \rightarrow _{\mathcal {B}}^{} q$ for some constant *a* and hence we can take $s = t = a$. For the induction step we consider four cases, depending on which inference rule is used to derive $p \leadsto q$. First suppose $[\textsf{c}]$ is used. So there exist transition rules $f(p_1,\dotsc ,p_{n}) \rightarrow p$ in $\mathcal {A}$ and $f(q_1,\dotsc ,q_{n}) \rightarrow q$ in $\mathcal {B}$ such that $p_i \leadsto q_i$ for $1 \leqslant i \leqslant n$. The induction hypothesis yields ground terms $s_1,\dotsc ,s_{n}$, $t_1,\dotsc ,t_{n}$ such that $(s_i,t_i) \in L(\mathcal {G}^-)^+$, $s_i \in L(\mathcal {A},p_i)$, and $t_i \in L(\mathcal {B},q_i)$ for $1 \leqslant i \leqslant n$. Let $s = f(s_1,\dotsc ,s_{n})$ and $t = f(t_1,\dotsc ,t_{n})$. We have $s \in L(\mathcal {A},p)$ and $t \in L(\mathcal {B},q)$. Moreover, $(s,t) \in L(\mathcal {G}^-)^+$ because the transitive closure of a parallel relation is parallel. Next suppose $[\textsf{a}]$ is applied to derive $p \leadsto q$. So there exists a state $p'$ such that . The induction hypothesis yields ground terms *s* and *t* such that $(s,t) \in L(\mathcal {G}^-)^+$, $s \in L(\mathcal {A},p')$, and $t \in L(\mathcal {B},q)$. Hence also $s \in L(\mathcal {A},p)$. The reasoning for $[\textsf{b}]$ is similar. The final case is the transitivity rule $[\textsf{t}]$. So $p \leadsto r$ and $r \leadsto q$ for some state *r*. The induction hypothesis yields terms *s*, *t*, *u*, *v* such that $(s,u), (v,t) \in L(\mathcal {G}^-)^+$, $s \in L(\mathcal {A},p)$, $u \in L(\mathcal {B},r)$, $v \in L(\mathcal {A},r)$, and $t \in L(\mathcal {B},q)$. From $u \in L(\mathcal {B},r)$ and $v \in L(\mathcal {A},r)$ we infer $(u,v) \in L(\mathcal {G}^-)$. Together with $(s,u), (v,t) \in L(\mathcal {G}^-)^+$, we obtain the desired $(s,t) \in L(\mathcal {G}^-)^+$. $\square$

#### Lemma 14

Let $\mathcal {G}= (\mathcal {A},\mathcal {B})$ be a GTT. Let $\mathcal {G}_+ = (\mathcal {A}_+,\mathcal {B}_+)$. If $s \rightarrow _{\mathcal {A}_+}^{*} q$ then $t \rightarrow _{\mathcal {A}}^{*} q$ for some ground term *t* with $(s,t) \in L(\mathcal {G})^+$. 

#### Proof

We proceed by induction on the length of the reduction $s \rightarrow _{\mathcal {A}_+}^{*} p$. If the last step is an epsilon transition $q \rightarrow p$ then the induction hypothesis yields a ground term *u* with $(s,u) \in L(\mathcal {G})^+$ and $u \in L(\mathcal {A},q)$. If $q \rightarrow p$ is a transition from $\mathcal {A}$ then $u \in L(\mathcal {A},p)$, and we conclude by letting $t = u$; otherwise, $q \rightarrow p$ must come from $\Delta _+(\mathcal {B},\mathcal {A})$, and using Lemma 13 we obtain ground terms *v* and *w* with $v \in L(\mathcal {B},q)$, $w \in L(\mathcal {A},p)$, and $(v,w) \in L(\mathcal {G})^+$. This implies $(u,v) \in L(\mathcal {G})$ and thus $(s,w) \in L(\mathcal {G})^+$ by transitivity. Letting $t = w$ gives the desired result. If the last step is not an $\varepsilon$-transition, then it must be a transition $f(p_1,\dotsc ,p_{n}) \rightarrow p$ from $\mathcal {A}$, and we have $s = f(s_1,\dotsc ,s_{n})$ for suitable $s_1,\dotsc ,s_{n}$. We apply the induction hypothesis to each argument position, resulting in $t_1,\dotsc ,t_{n}$ with $(s_i,t_i) \in L(\mathcal {G})^+$ and $t_i \in L(\mathcal {A},p_i)$ for $1 \leqslant i \leqslant n$. Let $t = f(t_1,\dotsc ,t_{n})$. We have $t \in L(\mathcal {A},p)$. Since $L(\mathcal {G})^+$ is transitive and closed under contexts, we obtain $(s,t) \in L(\mathcal {G})^*$. Since $L(\mathcal {G})$ is reflexive, we actually have $(s,t) \in L(\mathcal {G})^+$ as desired. $\square$

#### Lemma 15

Let $$\mathcal {G}= (\mathcal {A},\mathcal {B})$$ be a GTT. If $$\mathcal {G}_+ = (\mathcal {A}_+,\mathcal {B}_+)$$ then $$\Delta _\varepsilon (\mathcal {A}_+,\mathcal {B}_+)$$$$= \Delta _+(\mathcal {A},\mathcal {B})$$. 

#### Proof

We first show $\Delta _\varepsilon (\mathcal {A}_+,\mathcal {B}_+) \subseteq \Delta _+(\mathcal {A},\mathcal {B})$ via induction on the relation $\leadsto$ defined by the inference rules in Fig. 3. We proceed by case analysis, so assume $(p,q) \in \Delta _\varepsilon (\mathcal {A}_+,\mathcal {B}_+)$ is derived from a congruence step $[\textsf{c}]$. Hence we obtain $(p,q) \in \Delta _+(\mathcal {A},\mathcal {B})$ by a congruence step $[\textsf{c}]$ of Fig. 5, the fact that the constructions only add $\varepsilon$-transitions, and the induction hypothesis. Next assume that we derived $(q,r) \in \Delta _\varepsilon (\mathcal {A}_+,\mathcal {B}_+)$ by an $\varepsilon$-step $[\textsf{a}]$. So $p \rightarrow _{\mathcal {A}_+} q$ and $p \leadsto r$. We have $\mathcal {A}_+ = \mathcal {A}\cup \Delta _+(\mathcal {B},\mathcal {A})$. The result trivially follows for $p \rightarrow _{\!\mathcal {A}}q$. So let $(p,q) \in \Delta _+(\mathcal {B},\mathcal {A})$. Hence $(q,p) \in \Delta _+(\mathcal {A},\mathcal {B})$. The induction hypothesis yields $(p,r) \in \Delta _+(\mathcal {A},\mathcal {B})$ and therefore $(q,r) \in \Delta _+(\mathcal {A}, \mathcal {B})$ using the transitivity rule $[\textsf{t}]$. The $\varepsilon$-step $[\textsf{b}]$ case is obtained in the same way.

For the reverse inclusion we use induction on the relation $\leadsto$ defined by the inference rules in Fig. 5 and argue in a similar fashion. Hence $\Delta _\varepsilon (\mathcal {A}_+,\mathcal {B}_+) = \Delta _+(\mathcal {A},\mathcal {B})$ as desired. $\square$

#### Theorem 8

()  Anchored GTT relations are effectively closed under modified transitive closure. 

#### Proof

Let $\mathcal {G}= (\mathcal {A},\mathcal {B})$ be a GTT. We show . First let $(s,t) \in L_a(\mathcal {G}_+)$. So there exists a state *q* such that $s \rightarrow _{\mathcal {A}_+}^{*} q$ and $t \rightarrow _{\mathcal {B}_+}^{*} q$. Lemma 14 yields a ground term *u* such that $u \rightarrow _{\mathcal {A}}^{*} q$ and $(s,u) \in L(\mathcal {G})^+$. Applied to $\mathcal {G}^- = (\mathcal {B},\mathcal {A})$, Lemma 14 yields a ground term *v* such that $v \rightarrow _{\mathcal {B}}^{*} q$ and $(t,v) \in L(\mathcal {G}^-)^+$. Hence $(u,v) \in L_a(\mathcal {G})$ and $(v,t) \in L(\mathcal {G})^+$. Consequently, $(s,t) \in L(\mathcal {G})^+ \mathrel {\circ } L_a(\mathcal {G}) \mathrel {\circ } L(\mathcal {G})^+$ and, using Lemma 12, .

For the other direction we apply the modified composition operation  of Definition 10 with ${\bowtie }_1 = {\bowtie }_2 = L_a(\mathcal {G}_+)$ and obtainwith the help of Lemma 15. Note that we do not get equality, as one direction in the proof of Lemma 11 requires disjoint state sets. Since $L_a(\mathcal {G}) \subseteq L_a(\mathcal {G}_+)$ we also have

$$\begin{aligned} L_a(\mathcal {G}) \mathrel {\circ } L(\mathcal {G}_+) \,\cup \, L(\mathcal {G}_+) \mathrel {\circ } L_a(\mathcal {G}) \,\subseteq \, L_a(\mathcal {G}_+) \end{aligned}$$

At this point we can use the following well-known result in Kleene algebra

$$\begin{aligned} A \,\subseteq \, X ~\wedge ~ {B \mathrel {\circ } X} \,\subseteq \, X ~\wedge ~ {X \mathrel {\circ } C} \,\subseteq \, X \quad \implies \quad {B^* \mathrel {\circ } A \mathrel {\circ } C^*} \,\subseteq \, X \end{aligned}$$

with $A = L_a(\mathcal {G})$, $B = C = L(\mathcal {G})$, and $X = L_a(\mathcal {G}_+)$. Since $L(\mathcal {G})^* = L(\mathcal {G})^+$, we are done. $\square$

#### Example 15

For the GTT $\mathcal {G}= (\mathcal {A},\mathcal {B})$ of Example 11 we have $\Delta _+(\mathcal {A},\mathcal {B}) = \Delta _\varepsilon (\mathcal {A},\mathcal {B})$. Hence $\mathcal {G}_+ = (\mathcal {A}_+,\mathcal {B}_+)$ adds the pairs of $\Delta _+(\mathcal {B},\mathcal {A}) = \{(5,0),(5,1),(4,0),(6,0), (6,2),(5,4),(6,4)\}$ as $\varepsilon$-transitions to $\mathcal {A}$ and those of $\Delta _+(\mathcal {A},\mathcal {B}) = \Delta _+(\mathcal {B},\mathcal {A})^-$ to $\mathcal {B}$. We have $(\textsf{g}(\textsf{a},\textsf{b}),\textsf{f}(\textsf{b})) \in L_a(\mathcal {G}_+)$ as $\textsf{g}(\textsf{a},\textsf{b}) \rightarrow _{\mathcal {A}_+}^{*} 6$ and $\textsf{f}(\textsf{b}) \rightarrow _{\mathcal {B}_+} \textsf{f}(4) \rightarrow _{\mathcal {B}_+} \textsf{f}(5) \rightarrow _{\mathcal {B}_+} 6$. The term pair $(\textsf{f}(\textsf{a}),\textsf{f}(\textsf{b}))$ does not belong to $L_a(\mathcal {G}_+)$.

The penultimate operation on anchored GTT relations that we consider is complement. This requires the determinization of pair automata.

#### Lemma 16

For every pair automaton $\mathcal {P}= (Q,\mathcal {A},\mathcal {B})$ there exist deterministic tree automata $\mathcal {A}'$ and $\mathcal {B}'$ and a binary relation $Q^d$ such that $L(\mathcal {P}) = L((Q^d,\mathcal {A}',\mathcal {B}'))$. 

#### Proof

We use the subset construction to determinize $\mathcal {A}$ and $\mathcal {B}$ into equivalent deterministic tree automata $\mathcal {A}'$ and $\mathcal {B}'$. As the binary state relation we take $Q^d = \{(X,Y) \mid (p,q) \in Q$ for some $p \in X \subseteq Q_A$ and $q \in Y \subseteq Q_B\}$. We have $L(\mathcal {P}) = L((Q^d,\mathcal {A}',\mathcal {B}'))$ by the correctness of the subset construction. $\square$

#### Theorem 9

($A~\mathsf {::=}~A^c$)  Anchored GTT relations are effectively closed under complement. 

#### Proof

Let $\mathcal {G}$ be an anchored GTT. According to Lemma 16 we may assume that $L(\mathcal {G})$ is accepted by a deterministic pair automaton $\mathcal {P}= (Q,\mathcal {A},\mathcal {B})$. Without loss of generality we may further assume that $\mathcal {A}$ and $\mathcal {B}$ are completely defined. It follows that $L(\mathcal {P})^c = (Q^c,\mathcal {A},\mathcal {B})$ where $Q^c = (Q_A \times Q_B) {\setminus } Q$. $\square$

It is worth noting that GTT relations are *not* closed under complement [8, Exercise 3.4].

#### Example 16

For the pair automaton $\mathcal {P}_\mathcal {R}= (Q,\mathcal {A},\mathcal {B})$ of Example 10 we have $Q = \{(1,4),(2,4),(3,2)\}$. Determinizing $\mathcal {A}$ yields the tree automaton $\mathcal {A}'$ with the following transition rules:

$$\begin{aligned} \textsf{a}&\rightarrow A&\textsf{b}&\rightarrow B&\textsf{f}(X)&\rightarrow {\left\{ \begin{array}{ll} C &{}\text {if }X = A \\ B &{}\text {otherwise} \end{array}\right. }&\textsf{g}(X,Y)&\rightarrow {\left\{ \begin{array}{ll} D &{}\text {if }X = A \\ B &{}\text {otherwise} \end{array}\right. } \end{aligned}$$

for all $X, Y \in \{A,B,C,D\}$. Here $A = \{0,1\}$, $B = \{0\}$, $C = \{0,2\}$, and $D = \{0,3\}$. Next we determinize $\mathcal {B}$ to obtain the tree automaton $\mathcal {B}'$ consisting of the following transition rules:

$$\begin{aligned} \textsf{a}&\rightarrow E&\textsf{b}&\rightarrow F&\textsf{f}(X)&\rightarrow {\left\{ \begin{array}{ll} G &{}\text {if }X = E \\ H &{}\text {otherwise} \end{array}\right. }&\textsf{g}(X,Y)&\rightarrow H \end{aligned}$$

for all $X, Y \in \{E,F,G,H\}$. Here $E = \{1\}$, $F = \{4\}$, $G = \{2\}$, and $H = \varnothing$. The transition rules for $\textsf{g}$ are added to make $\mathcal {B}'$ completely defined. Now the complement $L(\mathcal {G})^c$ of $L(\mathcal {G})$ is accepted by the pair automaton $(Q',\mathcal {A}',\mathcal {B}')$ with

$$\begin{aligned} Q' = (\{A,B,C,D\} \times \{E,F,G,H\}) \setminus \{(A,F),(C,F),(D,G)\} \end{aligned}$$

The final closure property of anchored GTT relations that we mention is intersection.

#### Lemma 17

($A~\mathsf {::=}~A \cap A$)  Anchored GTT relations are effectively closed under intersection.

#### Proof

This follows from Theorem 9 and Lemma 10. $\square$

The formalized proof uses a more efficient product construction, to avoid the subset construction of the complement.

### Regular Relations

We continue with operations on regular relations. Again, most of the results and constructions are known. We provide detailed proofs that form the basis of the formalization. The following lemma takes care of transforming anchored GTT relations into binary regular (i.e., $\textsf{RR}_{2}$) relations.

#### Theorem 10

($R~\mathsf {::=}~A$)  Every anchored GTT relation is an $\textsf{RR}_{2}$ relation. 

#### Proof

Let $\mathcal {G}= (\mathcal {A},\mathcal {B})$ be a GTT. We construct an $\textsf{RR}_{2}$ automaton that accepts $L_a(\mathcal {G})$. We use a product construction with states *pq* where *p* is a state of $\mathcal {A}$ or $\bot$, and *q* is a state of $\mathcal {B}$ or $\bot$; the state $\bot \bot$ is not used. The transitions are

$$\begin{aligned} fg(p_1q_1,\dots ,p_kq_k)&\rightarrow p q \\ f\bot (p_1\bot ,\dots ,p_n\bot )&\rightarrow p\bot \\ \bot g(\bot q_1,\dots ,\bot q_m)&\rightarrow \bot q \end{aligned}$$

for all $f(p_1,\dotsc ,p_{n}) \rightarrow p \in \mathcal {A}$ and $g(q_1,\dotsc ,q_{m}) \rightarrow q \in \mathcal {B}$, where $k = \max (n,m)$ and $p_i = \bot$ if $n < i \leqslant k$ and $q_j = \bot$ if $m < j \leqslant k$, and

$$\begin{aligned} pq&\rightarrow p'q{} & {} \text {for all }p \rightarrow p' \in \mathcal {A}\text { and }q \in Q_\mathcal {B}\cup \{\bot \} \\ pq&\rightarrow pq'{} & {} \text {for all }q \rightarrow q' \in \mathcal {B}\text { and }p \in Q_\mathcal {A}\cup \{\bot \} \end{aligned}$$

These transitions accept $\langle s, t\rangle$ in state *pq* if and only if $s \in L(\mathcal {A},p)$ and $t \in L(\mathcal {B},q)$. As final states we pick *pp* with $p \in Q_\mathcal {A}\cap Q_\mathcal {B}$. A straightforward induction proof reveals that the resulting tree automaton accepts $L_a(\mathcal {G})$. $\square$

We illustrate the construction on our leading example.

#### Example 17

For the anchored GTT $\mathcal {G}$ of Example 11 we obtain the $\textsf{RR}_{2}$ automaton $\mathcal {A}= (\mathcal {F}^{(2)},Q,Q_f,\Delta )$ with $Q = (\{0,1,2,3,4,6,\bot \} \times \{4,5,6,\bot \}) {\setminus } \{\bot \bot \}$, $Q_f = \{44,66\}$, and $\Delta$ consisting of the following transition rules:

$$\begin{aligned} \textsf{aa}&\rightarrow 05&\textsf{ab}&\rightarrow 04&\textsf{af}(\bot 5)&\rightarrow 06 \\ \textsf{aa}&\rightarrow 15&\textsf{ab}&\rightarrow 14&\textsf{af}(\bot 5)&\rightarrow 16 \\ \textsf{ba}&\rightarrow 05&\textsf{bb}&\rightarrow 04&\textsf{bf}(\bot 5)&\rightarrow 06 \\ \textsf{fa}(0\bot )&\rightarrow 05&\textsf{fb}(0\bot )&\rightarrow 04&\textsf{ff}(05)&\rightarrow 06 \\ \textsf{fa}(1\bot )&\rightarrow 25&\textsf{fb}(1\bot )&\rightarrow 24&\textsf{ff}(15)&\rightarrow 26 \\ \textsf{ga}(0\bot ,0\bot )&\rightarrow 05&\textsf{gb}(0\bot ,0\bot )&\rightarrow 04&\textsf{gf}(05,0\bot )&\rightarrow 06 \\ \textsf{ga}(1\bot ,0\bot )&\rightarrow 35&\textsf{gb}(1\bot ,0\bot )&\rightarrow 34&\textsf{gf}(15,0\bot )&\rightarrow 36 \\ \textsf{a}\bot&\rightarrow 0\bot&\textsf{b}\bot&\rightarrow 0\bot&\bot \textsf{a}&\rightarrow \bot 5 \\ \textsf{a}\bot&\rightarrow 1\bot{} & {} {}&\bot \textsf{b}&\rightarrow \bot 4 \\ \textsf{f}\bot (0\bot )&\rightarrow 0\bot&\textsf{f}\bot (1\bot )&\rightarrow 2\bot&\bot \textsf{f}(\bot 5)&\rightarrow \bot 6 \\ \textsf{g}\bot (0\bot ,0\bot )&\rightarrow 0\bot&\textsf{g}\bot (1\bot ,0\bot )&\rightarrow 3\bot \\ 14&\rightarrow 44&24&\rightarrow 44&34&\rightarrow 64 \\ 15&\rightarrow 45&25&\rightarrow 45&35&\rightarrow 65 \\ 16&\rightarrow 46&26&\rightarrow 46&36&\rightarrow 66 \\ 1\bot&\rightarrow 4\bot&2\bot&\rightarrow 4\bot&3\bot&\rightarrow 6\bot \end{aligned}$$

We have

$$\begin{aligned} \langle \textsf{g}(\textsf{a},\textsf{f}(\textsf{b})), \textsf{f}(\textsf{a})\rangle&= \textsf{gf}(\textsf{aa},\textsf{f}\bot (\textsf{b}\bot )) \rightarrow _{\Delta }^{*} \textsf{gf}(15,\textsf{f}\bot (0\bot )) \rightarrow _{\Delta }\textsf{gf}(15,0\bot ) \rightarrow _{\Delta }^{*} 66 \end{aligned}$$

The various context closure operations are taken care of in the following general result.

#### Theorem 11

($R~\mathsf {::=}~R^{n}_p$)  If *R* is an $\textsf{RR}_{2}$ relation then $R_{p}^{n}$ is an $\textsf{RR}_{2}$ relation, for all $n \in \{{\geqslant }, 1, {>}\}$ and $p \in \{{\geqslant }, \varepsilon , {>}\}$. 

#### Proof

Let $\mathcal {A}= (\mathcal {F}^{(2)},Q,Q_f,\Delta )$ be the $\textsf{RR}_{2}$ automaton that accepts *R*. We add two new states $*$ and $\checkmark$. In the former the encoding of the identity relation on ground terms will be accepted. The latter will serve as the unique final state (unless specified otherwise). This is achieved by extending $\Delta$ with the transitions ${f\!f}(*,\dots ,*) \rightarrow *$ for every $f \in \mathcal {F}$ and $q \rightarrow \checkmark$ for every $q \in Q_f$. The resulting automaton $\mathcal {A}' = (\mathcal {F}^{(2)},Q \cup \{\checkmark ,*\},\{\checkmark \},\Delta ')$ is equivalent to $\mathcal {A}$ and the starting point for the various context closure operations.

- For $n = 1$ and $p = {\geqslant }$ we extend $\Delta$ with all rules of the form
  $$\begin{aligned} {f\!f}(*,\dots ,*,\checkmark ,*,\dots ,*) \rightarrow \checkmark \end{aligned}$$
- For $p = {>}$ we need a new final state $\checkmark '$ to ensure that the surrounding context is non-empty:
  $$\begin{aligned} {f\!f}(*,\dots ,*,\checkmark ,*,\dots ,*)&\rightarrow \checkmark '&{f\!f}(*,\dots ,*,\checkmark ',*,\dots ,*)&\rightarrow \checkmark ' \end{aligned}$$
  This is sufficient for $n = 1$. For $n = {>}$ we add the single $\varepsilon$-transition $\checkmark \rightarrow *$ and for $n = {\geqslant }$ we additionally add a new final state $*'$ together with transition rules ensuring that the accepted relation is reflexive:
  $$\begin{aligned} {f\!f}(*',\dots ,*')&\rightarrow *' \end{aligned}$$
- For $n = p = {\geqslant }$ we make $*$ the new (and only) final state and add the $\varepsilon$-transition $\checkmark \rightarrow *$.
- For $p = \varepsilon$ and $n \in \{1,{>}\}$ we have ${R_{p}^{n}} = R$ and thus we can just take the $\textsf{RR}_{2}$ automaton *A*. For $n = {\geqslant }$ we have ${R_{p}^{n}} = {R^=}$ and declare $*$ as an additional final state.
- In the remaining case we have $p = {\geqslant }$ and $n = {>}$. We extend $\Delta$ with all rules of the form
  $$\begin{aligned} {f\!f}(*,\dots ,*,\checkmark ,*,\dots ,*) \rightarrow \checkmark \end{aligned}$$
  and the single $\varepsilon$-transition $\checkmark \rightarrow *$.

The proof details can be found in the formalization. $\square$

#### Example 18

The following transition rules are added to the $\textsf{RR}_{2}$ automaton of Example 17 to model the relation :

$$\begin{aligned} \textsf{aa}&\rightarrow *&44&\rightarrow \checkmark&\textsf{ff}(\checkmark )&\rightarrow \checkmark '&\textsf{ff}(\checkmark ')&\rightarrow \checkmark ' \\ \textsf{bb}&\rightarrow *&66&\rightarrow \checkmark&\textsf{gg}(\checkmark ,*)&\rightarrow \checkmark '&\textsf{gg}(\checkmark ',*)&\rightarrow \checkmark ' \\ \textsf{ff}(*)&\rightarrow *&\checkmark&\rightarrow *&\textsf{gg}(*,\checkmark )&\rightarrow \checkmark '&\textsf{gg}(*,\checkmark ')&\rightarrow \checkmark ' \\ \textsf{gg}(*,*)&\rightarrow * \end{aligned}$$

The encoding of the term pair $(\textsf{g}(\textsf{f}(\textsf{a}),\textsf{f}(\textsf{a})),\textsf{g}(\textsf{b},\textsf{f}(\textsf{b})))$ is accepted: $\textsf{gg}(\textsf{fb}(\textsf{a}\bot ),\textsf{ff}(\textsf{ab})) \rightarrow ^{*} \textsf{gg}(\textsf{fb}(1\bot ),\textsf{ff}(14)) \rightarrow ^{*} \textsf{gg}(24,\textsf{ff}(44)) \rightarrow ^{*} \textsf{gg}(44,\textsf{ff}(\checkmark )) \rightarrow ^{*} \textsf{gg}(\checkmark ,\checkmark ') \rightarrow \textsf{gg}(*,\checkmark ') \rightarrow \checkmark '$.

We present one more operation that turns a regular set into an $\textsf{RR}_{2}$ relation. Here $=_T$ consists of all pairs (*t*, *t*) with $t \in T$.

#### Lemma 18

($R~\mathsf {::=}~{=_T}$)  If $T \subseteq \mathcal {T}(\mathcal {F})$ is regular then $=_T$ is an $\textsf{RR}_{2}$ relation. 

#### Proof

Let $A = (\mathcal {F},Q,Q_f,\Delta )$ be a tree automaton that accepts *T*. We turn *A* into the automaton $B = (\mathcal {F}^{(2)},Q,Q_f,\Delta ')$, where $\Delta '$ is obtained from $\Delta$ by modifying every transition rule $f(p_1,\dotsc ,p_{n}) \rightarrow q$ of $\Delta$ into $ff(p_1,\dotsc ,p_{n}) \rightarrow q$. The $\varepsilon$-transitions of $\Delta$ are kept. It is a trivial exercise to show that $L(B) \,=\, {=_{L(A)}} \,=\, {=_T}$. $\square$

The following result is an immediate consequence of the corresponding closure properties on regular sets (Theorem 1).

#### Theorem 12

($R~\mathsf {::=}~R \cup R \mid R \cap R$)  The class of *n*-ary regular relations is effectively closed under union and intersection for any $n \geqslant 0$. 

The final closure operations on regular relations are required for the logical structure of formulas in the first-order theory of rewriting.

#### Theorem 13

($R~\mathsf {::=}~R^c$)  The class of regular relations is effectively closed under complement.  

Given a regular relation *R*, its complement is denoted by $R^c$. Note that $\langle R^c\rangle \ne \langle R\rangle ^c$. The former is the topic of Theorem 13 and is used to model logical negation.

#### Proof

Let $R \subseteq \mathcal {T}(\mathcal {F})^n$ be a regular relation. We have $\langle R^c\rangle = \langle R\rangle ^c {\setminus } W^c$ where

$$\begin{aligned} W = \{t \in \mathcal {T}(\mathcal {F}^{(n)}) \mid t = \langle t_1,\dotsc ,t_{n}\rangle \text { for some }t_1,\dotsc ,t_{n} \in \mathcal {T}(\mathcal {F})\} \end{aligned}$$

is the set of encodings of *n*-tuples of ground terms. It is not difficult to show that *W* is regular. The set $\langle R\rangle$ is regular by assumption. Hence the regularity of $\langle R^c\rangle$ is a consequence of Theorem 1. $\square$

#### Definition 13

Let *R* be an *n*-ary relation over $\mathcal {T}(\mathcal {F})$. If $1 \leqslant i \leqslant n+1$ then the *i*-th cylindrification of *R* is the relation

$$\begin{aligned} C_i(R)&= \{(t_1,\dots ,t_{i-1},u,t_i,\dots ,t_n) \mid (t_1,\dotsc ,t_{n}) \in R \text { and }u \in \mathcal {T}(\mathcal {F})\} \end{aligned}$$

Moreover, if $\sigma$ is a permutation on $\{1,\dotsc ,n\}$ then

$$\begin{aligned} \sigma (R)&= \{(t_{\sigma (1)},\dots ,t_{\sigma (n)}) \mid (t_1,\dotsc ,t_{n}) \in R\} \end{aligned}$$

#### Theorem 14

The class of regular relations is effectively closed under cylindrification and permutation. 

In [8, Proposition 3.2.12] the closure under cylindrification is obtained via an inverse homomorphic image, resulting in a shorter proof. The proof of the latter operates on completely defined deterministic tree automata. The (formalized) proof below operates on arbitrary tree automata.

#### Proof

Let $\mathcal {A}= (\mathcal {F}^{(n)},Q,Q_f,\Delta )$ be a tree automaton that accepts $\langle R\rangle$. We construct tree automata that accept $\langle C_i(R)\rangle$ and $\langle \sigma (R)\rangle$. We first consider permutation. Let $\sigma$ be a permutation on $\{1,\dotsc ,n\}$ and define $\mathcal {A}_\sigma = (\mathcal {F}^{(n)},Q,Q_f,\Delta _\sigma )$ where $\Delta _\sigma$ is obtained from $\Delta$ by replacing every transition rule of the form $f_1 \cdots f_n(p_1,\dotsc ,p_{m}) \rightarrow q$ with $f_{\sigma (1)} \cdots f_{\sigma (n)}(p_1,\dotsc ,p_{m}) \rightarrow q$. Epsilon transitions in $\Delta$ are not affected. To conclude $L(\mathcal {A}_\sigma ) = \langle \sigma (R)\rangle$, we first define the effect of $\sigma$ on terms in $\mathcal {T}(\mathcal {F}^{(n)})$:

$$\begin{aligned} \sigma (t) \,=\, f_{\sigma (1)} \cdots f_{\sigma (n)}(\sigma (t_1),\dotsc ,\sigma (t_m)) \end{aligned}$$

for $t = f_1 \cdots f_n(t_1,\dotsc ,t_{m})$. The following preliminary fact 
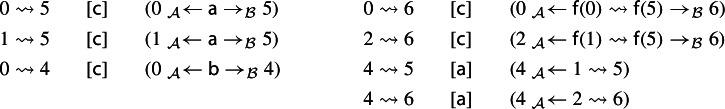


is proved as follows. We have

$$\begin{aligned} \mathcal {P}\textsf{os}(\langle t_{\sigma (1)},\dotsc ,t_{\sigma (n)}\rangle ) \,=\, \mathcal {P}\textsf{os}(t_1) \cup \cdots \cup \mathcal {P}\textsf{os}(t_n) \,=\, \mathcal {P}\textsf{os}(\langle t\rangle ) \,=\, \mathcal {P}\textsf{os}(\sigma (\langle t\rangle )) \end{aligned}$$

and, for every position $p \in \mathcal {P}\textsf{os}(\langle t_{\sigma (1)},\dotsc ,t_{\sigma (n)}\rangle )$,

$$\begin{aligned} \langle t_{\sigma (1)},\dotsc ,t_{\sigma (n)}\rangle (p) \,=\, f_1 \cdots f_n \,=\, \sigma (\langle t\rangle )(p) \end{aligned}$$

where $f_i = t_{\sigma (i)}(p)$ if $p \in \mathcal {P}\textsf{os}(t_{\sigma (i)})$ and $f_i = \bot$ otherwise. We now prove

6 $$\begin{aligned} \langle t_1,\dotsc ,t_{n}\rangle \,\rightarrow _{\!\mathcal {A}}^{*}\, q \quad \iff \quad \langle t_{\sigma (1)},\dotsc ,t_{\sigma (n)}\rangle \,\rightarrow _{\sigma (\mathcal {A})}^{*}\, q \end{aligned}$$

for all terms $t_1,\dotsc ,t_{n} \in \mathcal {T}(\mathcal {F}\cup \{\bot \})$ and states $q \in Q$. Suppose

$$\begin{aligned} \langle t_1,\dotsc ,t_{n}\rangle \,=\, f_1 \cdots f_n(u_1,\dotsc ,u_{m}) \,\rightarrow _{\!\mathcal {A}}^{*}\, q \end{aligned}$$

So there exists a transition rule $f_1 \cdots f_n(q_1,\dotsc ,q_{m}) \rightarrow p \in \Delta$ with $p \rightarrow _{\!\mathcal {A}}^{*} q$ and $u_i \rightarrow _{\!\mathcal {A}}^{*} q_i$ for all $1 \leqslant i \leqslant m$. We have $f_{\sigma (1)} \cdots f_{\sigma (n)}(q_1,\dotsc ,q_{m}) \rightarrow p \in \Delta _\sigma$ and $p \rightarrow _{\sigma (\mathcal {A})}^{*} q$. Using ($*_\sigma$) the induction hypothesis yields $\sigma (u_i) \rightarrow _{\sigma (\mathcal {A})}^{*} q_i$ for $1 \leqslant i \leqslant m$ and thus

$$\begin{aligned} \langle t_{\sigma (1)},\dotsc ,t_{\sigma (n)}\rangle \,=\, f_{\sigma (1)} \cdots f_{\sigma (n)}(\sigma (u_1),\dots ,\sigma (u_n)) \,\rightarrow _{\sigma (\mathcal {A})}^{*}\, q \end{aligned}$$

The converse is proved in a similar fashion. By specializing (6) to terms $t_1,\dotsc ,t_{n} \in \mathcal {T}(\mathcal {F})$ and states $q \in Q_f$ we obtain $L(\sigma (\mathcal {A})) \,=\, \{\sigma (\langle t_1,\dotsc ,t_{n}\rangle ) \mid \langle t_1,\dotsc ,t_{n}\rangle \in L(\mathcal {A})\} \,=\, L(\langle \sigma (R)\rangle )$.

Next we consider cylindrification. Let $i \in \{1,\dotsc ,n+1\}$. We define the tree automaton $\mathcal {A}_{C_i} = (\mathcal {F}^{(n+1)},(Q \cup \{\bot \}) \times \{\top ,\bot \},Q_f \times \{\top \},\Delta _{C_i})$ where $\bot$ is a fresh state and $\Delta _{C_i}$ is obtained from $\Delta$ by replacing every transition rule of the form

$$\begin{aligned} f_1 \cdots f_{i-1}f_i \cdots f_n(p_1,\dotsc ,p_{m})&\rightarrow q \end{aligned}$$

with the transitions

$$\begin{aligned} f_1 \cdots f_{i-1}gf_i \cdots f_n(p_1q_1,\dotsc ,p_mq_m,\dots ,p_kq_k)&\rightarrow q\top \\ f_1 \cdots f_{i-1} \bot f_i \cdots f_n(p_1\bot ,\dotsc ,p_m\bot )&\rightarrow q\bot \end{aligned}$$

for all *l*-ary $g \in \mathcal {F}$. Here $k = \max (m,l)$ is the arity of $f_1 \cdots f_{i-1}gf_i \cdots f_n$. Moreover, $p_j = \bot$ for all $m < j \leqslant k$, and

$$\begin{aligned} q_j = {\left\{ \begin{array}{ll} \top &{}\text {if }j \leqslant l \\ \bot &{}\text {if }j > l \end{array}\right. } \end{aligned}$$

for all $1 \leqslant j \leqslant k$. Additionally, $\Delta _{C_i}$ contains the transition rule

$$\begin{aligned} \bot \cdots \bot g \bot \cdots \bot (\bot \top ,\dotsc ,\bot \top ) \rightarrow \bot \top \end{aligned}$$

for every $g \in \mathcal {F}$. Here *g* is the *i*-th element in $\bot \cdots \bot g \bot \cdots \bot$. Finally, for every $\varepsilon$-transition $p \rightarrow q$ in $\Delta$ we add $p\top \rightarrow q\top$ and $p\bot \rightarrow q\bot$ to $\Delta _{C_i}$. The purpose of the second component $\bot /\top$ in states of $\mathcal {A}_{C_i}$ is to mark whether states are reached by terms where ($\top$) the *i*-th position in the encoded tuple is a term in $\mathcal {T}(\mathcal {F})$, or ($\bot$) it is $\bot$. In order to show $L(\mathcal {A}_{C_i}) = \langle C_i(R)\rangle$, we simplify the notation by considering $i = 1$, which entails no loss of generality as regular relations are closed under permutation. Again, first we define the effect of $C_1$ on terms in $\mathcal {T}(\mathcal {F}^{(1)}) \times \mathcal {T}(\mathcal {F}^{(n)})$:

$$\begin{aligned} C_1(s,t) \,=\, f f_1 \cdots f_n(C_1(s_1,u_1),\dotsc ,C_1(s_k,u_k)) \end{aligned}$$

for $s = f(s_1,\dotsc ,s_{l})$ and $t = f_1 \cdots f_n(u_1,\dotsc ,u_{m})$. Here $k = \max (l,m)$ is the arity of $f f_1 \cdots f_n$, $s_j = \bot$ for $l < j$ and $u_j = \bot ^n$ for $m < j$. By induction on $s \in \mathcal {T}(\mathcal {F}^{(1)})$ and $t \in \mathcal {T}(\mathcal {F}^{(n)})$ we show the preliminary statements

7 $$\begin{aligned} \mathcal {P}\textsf{os}(C_1(\bot ,t))&\,=\, \mathcal {P}\textsf{os}(t)&~&\text {and} ~~&C_1(\bot ,t)(p)&\,=\, \bot t(p)&~&\text {for all }p \in \mathcal {P}\textsf{os}(t) \end{aligned}$$

8 $$\begin{aligned} \mathcal {P}\textsf{os}(C_1(s,\bot ^n))&\,=\, \mathcal {P}\textsf{os}(s){} & {} \text {and}&C_1(s,\bot ^n)(p)&\,=\, s(p) \bot ^n{} & {} \text {for all }p \in \mathcal {P}\textsf{os}(s) \end{aligned}$$

Let $t = f_1 \cdots f_n(u_1,\dotsc ,u_{m})$. We have $C_1(\bot ,t) = \bot f_1 \cdots f_n(C_1(\bot ,u_1),\dotsc ,C_1(\bot ,u_k))$ and obtain $\mathcal {P}\textsf{os}(C_1(\bot ,u_i)) = \mathcal {P}\textsf{os}(u_i)$ and $C_1(\bot ,u_i)(q) = \bot u_i(q)$ for all $ip \in \mathcal {P}\textsf{os}(t)$ from the induction hypothesis. Note that $ip \in \mathcal {P}\textsf{os}(t)$ if and only if $p \in \mathcal {P}\textsf{os}(u_i)$. For $p = \varepsilon$ we have $C_1(\bot ,t)(p) = \bot f_1 \cdots f_n = \bot t(p)$. This establishes (7). The proof of (8) is similar and omitted. These statements are used to prove $\mathcal {P}\textsf{os}(C_1(s,t)) = \mathcal {P}\textsf{os}(s) \cup \mathcal {P}\textsf{os}(t)$ and $C_1(s,t)(p) = s(p)t(p)$ for all $p \in \mathcal {P}\textsf{os}(s) \cup \mathcal {P}\textsf{os}(t)$, by induction on $|s| + |t|$. Let $s = f(s_1,\dotsc ,s_{l})$ and $t = f_1 \cdots f_n(u_1,\dotsc ,u_{m})$. Let $k = \max (l,m)$ be the arity of $f f_1 \cdots f_n$. We have $\mathcal {P}\textsf{os}(C_1(s_i,u_i)) = \mathcal {P}\textsf{os}(s_i) \cup \mathcal {P}\textsf{os}(u_i)$ and $C_1(s_i,u_i)(p) = s_i(p)u_i(p)$ for all $p \in \mathcal {P}\textsf{os}(s_i) \cup \mathcal {P}\textsf{os}(u_i)$ for all $1 \leqslant i \leqslant k$. For $i \leqslant \min (l,m)$ this follows from the induction hypothesis and for $i > \min (l,m)$ this follows from (7) or (8). Moreover, $C_1(s,t)(\varepsilon ) = f f_1 \cdots f_n = s(\varepsilon )t(\varepsilon )$ so the second statement also holds for $p = \varepsilon$. From these statements we immediately obtain 
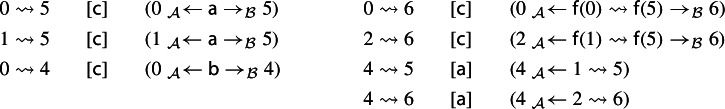


for all terms $s \in \mathcal {T}(\mathcal {F}^{(1)})$ and $t = \langle t_1,\dotsc ,t_{n}\rangle \in \mathcal {T}(\mathcal {F}^{(n)})$. The following two properties are easily proved by induction:

9 $$\begin{aligned} C_1(s,\bot ^n)&\,\rightarrow _{C_1(\mathcal {A})}^{*}\, \bot \top \end{aligned}$$

for all terms $s \in \mathcal {T}(\mathcal {F})$ and

10 $$\begin{aligned} t \,\rightarrow _{\!\mathcal {A}}^{*}\, q \quad \iff \quad C_1(\bot ,t)&\,\rightarrow _{C_1(\mathcal {A})}^{*}\, q\bot \end{aligned}$$

for all terms $t \in \mathcal {T}(\mathcal {F}^{(n)})$. For the first one we use induction on $s = f(s_1,\dotsc ,s_{l})$. We have $C_1(s,\bot ^n) = f\bot ^n(C_1(s_1,\bot ^n),\dots ,C_1(s_\ell ,\bot ^n))$ and obtain $C_1(s_i,\bot ^n) \rightarrow _{C_1(\mathcal {A})}^{*} \bot \top$ for $1 \leqslant l \leqslant n$ from the induction hypothesis. By construction $f\bot ^n(\bot \top ,\dotsc ,\bot \top ) \rightarrow \bot \top \in \Delta _{C_1}$. Hence $C_1(s,\bot ^n) \rightarrow _{C_1(\mathcal {A})}^{*} \bot \top$. The second property is proved by induction on $t = f_1 \cdots f_n(u_1,\dotsc ,u_{m})$. We have $C_1(\bot ,t) = \bot f_1 \cdots f_n(C_1(\bot ,u_1),\dots ,C_1(\bot ,u_m))$. First assume $t \rightarrow _{\!\mathcal {A}}^{*} q$. So there exists a transition rule $f_1 \cdots f_n(q_1,\dotsc ,q_{m}) \rightarrow p \in \Delta$ with $p \rightarrow _{\!\mathcal {A}}^{*} q$ and $u_i \rightarrow _{\!\mathcal {A}}^{*} q_i$ for all $1 \leqslant i \leqslant m$. The induction hypothesis yields $C_1(\bot ,u_i) \rightarrow _{C_1(\mathcal {A})}^{*} q_i\bot$ for $1 \leqslant i \leqslant m$. By construction $\bot f_1 \cdots f_n(q_1\bot ,\dotsc ,q_m\bot ) \rightarrow p\bot \in \Delta _{C_1}$ and $p\bot \rightarrow _{C_1(\mathcal {A})}^{*} q\bot$. Combining all this yields $C_1(\bot ,t) \rightarrow _{C_1(\mathcal {A})}^{*} q\bot$. For the converse, assume $C_1(\bot ,t) \rightarrow _{C_1(\mathcal {A})}^{*} q\bot$. So there exists a rule $\bot f_1 \cdots f_n(q_1\bot ,\dotsc ,q_m\bot ) \rightarrow p\bot \in \Delta _{C_1}$ with $p\bot \rightarrow _{C_1{\mathcal {A}}}^{*} q\bot$ and $C_1(\bot ,u_i) \rightarrow _{C_1(\mathcal {A})}^{*} q_i\bot$ for all $1 \leqslant i \leqslant m$. The induction hypothesis yields $u_i \rightarrow _{\!\mathcal {A}}^{*} q_i$ for $1 \leqslant i \leqslant m$. Furthermore, the transition rule $\bot f_1 \cdots f_n(q_1\bot ,\dotsc ,q_m\bot ) \rightarrow p\bot$ originates from $f_1 \cdots f_n(q_1,\dotsc ,q_{m}) \rightarrow p \in \Delta$ and we obtain $p\bot \rightarrow _{C_1(\mathcal {A})}^{*} q\bot$ from $p \rightarrow _{\!\mathcal {A}}^{*} q$. Hence $t \rightarrow _{\!\mathcal {A}}^{*} q$ as desired. This completes the proofs of (9) and (10). Next we prove

11 $$\begin{aligned} t \,\rightarrow _{\!\mathcal {A}}^{*}\, q \quad \iff \quad C_1(s,t)&\,\rightarrow _{C_1(\mathcal {A})}^{*}\, q\top \end{aligned}$$

for all $s \in \mathcal {T}(\mathcal {F})$, $t \in \mathcal {T}(\mathcal {F}^{(n)})$ and $q \in Q$. For the only-if direction we use induction on $t = f_1 \cdots f_n(u_1,\dotsc ,u_{m})$. Let $s = f(s_1,\dotsc ,s_{l})$. From $t \rightarrow _{\!\mathcal {A}}^{*} q$ we obtain $f_1 \cdots f_n(p_1,\dotsc ,p_{m}) \rightarrow p \in \Delta$ with $p \rightarrow _{\!\mathcal {A}}^{*} q$ and $u_i \rightarrow _{\!\mathcal {A}}^{*} p_i$ for all $1 \leqslant i \leqslant m$. We have

$$\begin{aligned} f f_1 \cdots f_n(p_1q_1,\dotsc ,p_mq_m,\dotsc ,p_kq_k) \rightarrow p\top \in \Delta _{C_1} \end{aligned}$$

by construction. Here $k = \max (l,m)$ is the arity of $f f_1 \cdots f_n$, $p_i = \bot$ for all $m < i \leqslant k$, $q_i = \top$ if $1 \leqslant i \leqslant l$ and $q_i = \bot$ if $l < i \leqslant k$. We have $p\top \rightarrow _{C_1(\mathcal {A})}^{*} q\top$ and $C_1(s,t) = f f_1 \cdots f_n(C_1(s_1,u_1),\dotsc ,C_1(s_k,u_k))$ with $s_i = \bot$ for $l < i \leqslant k$ and $u_i = \bot ^n$ for $m < i\,{\leqslant }\,k$. The induction hypothesis yields $C_1(s_i,u_i) \rightarrow _{C_1(\mathcal {A})}^{*} p_i\top$ for all $1 \leqslant i \leqslant \min (l,m)$. Note that $\top = q_i$. For $\min (l,m) < i \leqslant k$ we distinguish two cases.

- If $\min (l,m) = m$ then $m < i$ and thus $u_i = \bot ^n$. We obtain $C_1(s_i,u_i) \rightarrow _{C_1(\mathcal {A})}^{*} \bot \top$ from (9). Note that $p_i = \bot$ and $q_i = \top$.
- If $\min (l,m) = l$ then $l < i$ and thus $s_i = \bot$. We obtain $C_1(s_i,u_i) \rightarrow _{C_1(\mathcal {A})}^{*} p_i\bot$ from (10). Note that $q_i = \bot$.

So in all cases we have $C_1(s_i,u_i) \rightarrow _{C_1(\mathcal {A})}^{*} p_iq_i$. Hence

$$\begin{aligned} C_1(s,t) \,\rightarrow _{C_1(\mathcal {A})}^{*}\, f f_1 \cdots f_n(p_1q_1,\dotsc ,p_mq_m,\dotsc ,p_kq_k) \,\rightarrow _{C_1(\mathcal {A})}^{}\, p\top \,\rightarrow _{C_1(\mathcal {A})}^{*}\, q\top \end{aligned}$$

as desired. The if-direction of (11) is proved in a similar fashion. From

$$\begin{aligned} C_1(s,t) = f f_1 \cdots f_n(C_1(s_1,u_1),\dotsc ,C_1(s_k,u_k)) \,\rightarrow _{C_1(\mathcal {A})}^{*}\, q\top \end{aligned}$$

we obtain a rule $f f_1 \cdots f_n(p_1q_1,\dotsc ,p_mq_m,\dotsc ,p_kq_k) \rightarrow p\top \in \Delta _{C_1}$ with $p\top \rightarrow _{C_1(\mathcal {A})}^{*} q\top$ and $C_1(s_i,u_i) \rightarrow _{C_1(\mathcal {A})}^{*} p_iq_i$ for $1 \leqslant i \leqslant k$. We have $f_1 \cdots f_n(p_1,\dotsc ,p_{m}) \rightarrow p \in \Delta$ and $p \rightarrow _{\!\mathcal {A}}^{*} q$ due to the construction of $\Delta _{C_1}$. The induction hypothesis yields $u_i \rightarrow _{\!\mathcal {A}}^{*} p_i$ for $1 \leqslant i \leqslant m$ and thus $t = f_1 \cdots f_n(u_1,\dotsc ,u_{m}) \rightarrow _{\!\mathcal {A}}^{*} q$. Specializing (11) to terms $t = \langle t_1,\dotsc ,t_{n}\rangle$ with $t_1,\dotsc ,t_{n} \in \mathcal {T}(\mathcal {F})$ and $q \in Q_f$ yields $L(C_1(\mathcal {A})) = \{\langle s,t_1,\dotsc ,t_{n}\rangle \mid \langle t_1,\dotsc ,t_{n}\rangle \in L(\mathcal {A}) \text { and }s \in \mathcal {T}(\mathcal {F})\} = \langle C_1(R)\rangle$. $\square$

Note that for every $\textsf{RR}_{2}$ relation *R*, its inverse $R^-$ is the same as $\sigma (R)$ for the permutation $\sigma = (1 2)$.

#### Corollary 2

($R~\mathsf {::=}~R^-$)  The class of binary regular relations is effectively closed under inverse. $\square$

#### Example 19

Consider the $\textsf{RR}_{2}$ automaton $\mathcal {A}= (\mathcal {F}^{(2)},Q,Q_f,\Delta )$ of Example 17. We compute $C_2(\{(s,t,u) \mid s \rightarrow _{\varepsilon } u \text { and }t \in \mathcal {T}(\mathcal {F})\}$. To this end, we transform $\mathcal {A}$ by the construction in the above proof. This results in an automaton $\mathcal {B}= (\mathcal {F}^{(3)},Q',Q_f',\Delta ')$ with $Q' = (Q \cup \{\bot \}) \times \{\top ,\bot \}$, $Q_f' = \{44_\top ,66_\top \}$, and $\Delta '$ consisting of 183 transitions. Every non-$\varepsilon$-transition in $\Delta$ gives rise to five transitions in $\Delta '$. For instance, the transitions

$$\begin{aligned} \textsf{aaa}&\rightarrow 05_\top&\textsf{afa}(\bot _\top )&\rightarrow 05_\top&\textsf{aga}(\bot _\top ,\bot _\top )&\rightarrow 05_\top \\ \textsf{aba}&\rightarrow 05_\top&\textsf{a}\bot \textsf{a}&\rightarrow 05_\bot \end{aligned}$$

originate from $\textsf{aa} \rightarrow 05$ and the transitions

$$\begin{aligned} \bot \textsf{af}(\bot 5_\top )&\rightarrow \bot 6_\top&\bot \textsf{ff}(\bot 5_\top )&\rightarrow \bot 6_\top&\bot \textsf{gf}(\bot 5_\top ,\bot _\top )&\rightarrow \bot 6_\top \\ \bot \textsf{bf}(\bot 5_\top )&\rightarrow \bot 6_\top&\bot \bot \textsf{f}(\bot 5_\bot )&\rightarrow \bot 6_\bot \end{aligned}$$

originate from $\bot \textsf{f}(\bot 5) \rightarrow \bot 6$. Moreover, every $\varepsilon$-transition in $\Delta$ is duplicated in $\Delta '$. For instance, $25 \rightarrow 45$ gives rise to $25_\top \rightarrow 45_\top$ and $25_\bot \rightarrow 45_\bot$. Finally, $\Delta '$ contains the transitions

$$\begin{aligned} \bot \textsf{a}\bot&\rightarrow \bot _\top&\bot \textsf{b}\bot&\rightarrow \bot _\top&\bot \textsf{f}\bot (\bot _\top )&\rightarrow \bot _\top&\bot \textsf{g}\bot (\bot _\top ,\bot _\top )&\rightarrow \bot _\top&\end{aligned}$$

So in total there are $31 \times 5 + 12 \times 2 + 4 = 183$ transitions in $\Delta '$.

In Theorem 14 and its proof we have finally introduced all concepts needed to complete the proof that $\textsf{RR}_{n}$ relations are closed under projection (Theorem 2). It remains to be shown that $L(\mathcal {A}_{\Pi _i}) = \langle \Pi _i(R)\rangle$.

#### Proof of Theorem 2 (cont’d)

To simplify the notation, we consider $\Pi _1$ (which entails no loss of generality as regular relations are closed under permutation). Again, first we define the effect of $\Pi _1$ on terms in $\mathcal {T}(\mathcal {F}^{(n)})$:

$$\begin{aligned} \Pi _1(t) \,=\, f_2 \cdots f_n(\Pi _1(u_1),\dotsc ,\Pi _1(u_k)) \end{aligned}$$

for $t = f_1 \cdots f_n(u_1,\dotsc ,u_{m})$. Here $k \leqslant m$ is the arity of $f_2 \cdots f_n$. We show

12 $$\begin{aligned} \Pi _1(C_1(s,t)) \,=\, t \end{aligned}$$

for all terms $s \in \mathcal {T}(\mathcal {F}^{(1)})$ and $t \in \mathcal {T}(\mathcal {F}^{(n)})$ by induction on $|s| + |t|$. So let $s = f(s_1,\dotsc ,s_{l})$ and $t = f_1 \cdots f_n(u_1,\dotsc ,u_{m})$. We have

$$\begin{aligned} \Pi _1(C_1(s,t))&\,=\ \Pi _1(f f_1 \cdots f_n(C_1(s_1,u_1),\dotsc ,C_1(s_k,u_k))) \\&\,=\ f_1 \cdots f_n(\Pi _1(C_1(s_1,u_i)),\dotsc ,\Pi _1(C_1(s_m,u_m))) \\&\,=\ f_1 \cdots f_n(u_1,\dotsc ,u_{m}) \,=\ t \end{aligned}$$

Here $k = \max (l,m)$ is the arity of $f f_1 \cdots f_n$, $s_j = \bot$ for $l < j$, $u_j = \bot ^n$ for $m < j$, and the induction hypothesis is applied to $\Pi _1(C_1(s_i,u_i))$ for $1 \leqslant i \leqslant m$. Now we can easily show 
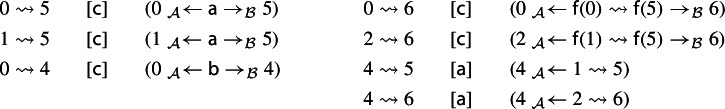


for all terms $\langle t_1,\dotsc ,t_{n}\rangle \in \mathcal {T}(\mathcal {F}^{(n)})$. From ($*_{C}$) in the proof of Theorem 14 we obtain

$$\begin{aligned} \langle t_1,t_2,\dotsc ,t_n\rangle \,=\, C_1(t_1,\langle t_2,\dotsc ,t_n\rangle ) \end{aligned}$$

and thus $\Pi _1(\langle t_1,\dotsc ,t_{n}\rangle ) = \Pi _1(C_1(t_1,\langle t_2,\dotsc ,t_n\rangle )) = \langle t_2,\dotsc ,t_n\rangle$ using (12). We now prove the following two statements:

13 $$\begin{aligned} t \,\rightarrow _{\!\mathcal {A}}^{*}\, q&\quad \implies \quad \Pi _1(t) \,\rightarrow _{\Pi _1(\mathcal {A})}^{*}\, q \end{aligned}$$

for all terms $t \in \mathcal {T}(\mathcal {F}^{(n)})$ and states $q \in Q$, and

14 $$\begin{aligned} u \,\rightarrow _{\Pi _1(\mathcal {A})}^{*}\, q&\quad \implies \quad t \,\rightarrow _{\!\mathcal {A}}^{*}\, q \text { for some term }t \in \mathcal {T}(\mathcal {F}^{(n)}) \text { with }\Pi _1(t) = u \end{aligned}$$

for all terms $u \in \mathcal {T}(\mathcal {F}^{(n)})$. We prove the first statement by induction on *t*. Suppose

$$\begin{aligned} t \,=\, f_1 \cdots f_n(u_1,\dotsc ,u_{m}) \,\rightarrow _{\!\mathcal {A}}^{*}\, q \end{aligned}$$

So there exist a transition rule $f_1 \cdots f_n(q_1,\dotsc ,q_{m}) \rightarrow p \in \Delta$ with $p \rightarrow _{\!\mathcal {A}}^{*} q$ such that $u_i \rightarrow _{\!\mathcal {A}}^{*} q_i$ for all $1 \leqslant i \leqslant m$. To simplify the reasoning, we assume that the condition $f_2 \cdots f_n \ne \bot ^{n-1}$ in the definition of $\Delta _{\Pi _1}$ is temporarily lifted. This entails that $f_2 \cdots f_n(q_1,\dotsc ,q_{k}) \rightarrow p$ is a transition rule in $\Delta _{\Pi _1}$. Here $k \leqslant m$ is the arity of $f_2 \cdots f_n$. We have $p \rightarrow _{\Pi _1(\mathcal {A})}^{*} q$. The induction hypothesis yields $\Pi (u_i) \,\rightarrow _{\Pi _1(\mathcal {A})}^{*}\, q_i$ for $1 \leqslant i \leqslant m$. Hence

$$\begin{aligned} \Pi _1(t) \,=\, f_2 \cdots f_n(\Pi _1(u_1),\dotsc ,\Pi _1(u_k)) \,\rightarrow _{\Pi _1(\mathcal {A})}^{*}\, f_2 \cdots f_n(q_1,\dotsc ,q_{k}) \,\rightarrow _{\Pi _1(\mathcal {A})}^{*}\, q \end{aligned}$$

as desired. For the second statement, suppose $u = f_2 \cdots f_n(u_1,\dotsc ,u_k) \rightarrow _{\Pi _1(\mathcal {A})}^{*} q$ and so there exists a transition rule $f_2 \cdots f_n(q_1,\dotsc ,q_{k}) \rightarrow p \in \Delta _{\Pi _1}$ with $p \rightarrow _{\Pi _1(\mathcal {A})}^{*} q$ and $u_i \rightarrow _{\Pi _1(\mathcal {A})}^{*} q_i$ for all $1 \leqslant i \leqslant k$. By construction of $\Pi _1(\mathcal {A})$, there exist a function symbol $f_1 \in \mathcal {F}\cup \{\bot \}$ and states $q_{k+1},\dotsc ,q_m$ such that $f_1f_2 \cdots f_n(q_1,\dotsc ,q_{m}) \rightarrow p \in \Delta$. Here $m \geqslant k$ is the arity of $f_1 \cdots f_n$. From the induction hypothesis we obtain terms $v_1,\dotsc ,v_{k} \in \mathcal {T}(\mathcal {F}^{(n)})$ such that $v_i \rightarrow _{\!\mathcal {A}}^{*} q_i$ and $\Pi _1(v_i) = u_i$ for $1 \leqslant i \leqslant k$. Because all states of $\mathcal {A}$ are reachable, there exist terms $v_{k+1},\dotsc ,v_m \in \mathcal {T}(\mathcal {F}^{(n)})$ such that $v_j \rightarrow _{\!\mathcal {A}}^{*} q_j$ for $k+1 \leqslant j \leqslant m$. Now let $t = f_1 \cdots f_n(v_1,\dotsc ,v_{m})$. We clearly have $t \rightarrow _{\!\mathcal {A}}^{*} f_1 \cdots f_n(q_1,\dotsc ,q_{m}) \rightarrow _{\!\mathcal {A}}^{*} p$ Moreover, $\Pi _1(t) = f_2 \cdots f_n(\Pi _1(v_1),\dotsc ,\Pi _1(v_k)) = f_2 \cdots f_n(u_1,\dotsc ,u_{k}) = u$. This concludes the proof of the two statements. Specializing statement (13) to $t = \langle t_1,\dotsc ,t_{n}\rangle$ where $t_1,\dotsc ,t_{n} \in \mathcal {T}(\mathcal {F})$ and states $q \in Q_f$ yields $\Pi _1(L(\mathcal {A})) \subseteq L(\Pi _1(\mathcal {A}))$. From statement (14) we conclude $L(\Pi _1(\mathcal {A})) \subseteq \Pi _1(L(\mathcal {A}))$ and hence

$$\begin{aligned} L(\Pi _1(\mathcal {A})) \,=\, \{\Pi _1(\langle t_1,\dotsc ,t_{n}\rangle ) \mid \langle t_1,\dotsc ,t_{n}\rangle \in L(\mathcal {A})\} \,=\, \langle \Pi _{1}(R)\rangle \end{aligned}$$

It remains to show that the automaton $\Pi _1(\mathcal {A})$ does not use any rule $\bot ^{n-1} \rightarrow p$ to accept terms when $n > 1$. Since $L(\Pi _1(\mathcal {A})) = \langle \Pi _1(R)\rangle$ and $\Pi _1(R) \subseteq \mathcal {T}(\mathcal {F})^{n-1}$, no term in $\langle \Pi _1(R)\rangle$ contains the function symbol $\bot ^{n-1}$. $\square$

### Normal Form Predicate

At this point we have formalized proofs for the constructs in the grammar in Fig. 1, with the exception of the normal form predicate ($T~\mathsf {::=}~\textsf{NF}$). This predicate can be defined in the first-order theory of rewriting as

$$\begin{aligned} \textsf{NF}(t) \quad \iff \quad \lnot \,\exists \,u\,(t \rightarrow u) \end{aligned}$$

which gives rise to the following procedure:

1. Using Theorems 4, 10 and 11 an $\textsf{RR}_{2}$ automaton is constructed that accepts the encoding of the rewrite relation $\rightarrow$.
2. Using Theorem 2 the $\textsf{RR}_{2}$ automaton of step 1 is projected into a tree automaton that accepts the set of reducible ground terms, corresponding to the subformula $\exists \,u\,(t \rightarrow u)$.
3. Complementation (Theorem 13) is applied to the automaton of step 2 to obtain a tree automaton that accepts the set of ground normal forms.

Since projection may transform a deterministic tree automaton into a non-deterministic one, this is inefficient. In this section we provide a direct construction of a tree automaton that accepts the set of ground normal forms of a left-linear TRS, which goes back to Comon [6], and present a formalized correctness proof. Throughout this section $\mathcal {R}$ is assumed to be left-linear.

We start with defining some preliminary concepts.

#### Definition 14

Given a signature $\mathcal {F}$, we write $\mathcal {F}_\bot$ for the extension of $\mathcal {F}$ with a fresh constant symbol $\bot$. Given $t \in \mathcal {T}(\mathcal {F},\mathcal {V})$, $t^\bot$ denotes the result of replacing all variables in *t* by $\bot$:

$$\begin{aligned} x^\bot&\,=\, \bot&f(t_1,\dotsc ,t_{n})^\bot&\,=\, f(t^\bot _1,\dotsc ,t^\bot _{n}) \end{aligned}$$

We define the partial order $\leqslant$ on $\mathcal {T}(\mathcal {F}_\bot )$ as the least congruence that satisfies $\bot \leqslant t$ for all terms $t \in \mathcal {T}(\mathcal {F}_\bot )$:The partial map ${\uparrow }:\mathcal {T}(\mathcal {F}_\bot )\times \mathcal {T}(\mathcal {F}_\bot )\rightarrow \mathcal {T}(\mathcal {F}_\bot )$ is defined as follows:

$$\begin{aligned} \bot \uparrow t&\,=\, t \uparrow \bot \,=\, t&f(t_1,\dotsc ,t_{n}) \uparrow f(u_1,\dotsc ,u_{n})&\,=\, f(t_1 \uparrow u_1,\dots ,t_n \uparrow u_n) \end{aligned}$$

It is not difficult to show that $t \uparrow u$ is the least upper bound of comparable terms *t* and *u*.

#### Definition 15

Let $\mathcal {R}$ be a TRS over a signature $\mathcal {F}$. We write $T^\bot$ for the set $\{t^\bot \mid t \mathrel {{\vartriangleleft }}\ell \text { for some }\ell \rightarrow r \in \mathcal {R}\} \cup \{\bot \}$. The set $T_\uparrow$ is obtained by closing $T^\bot$ under $\uparrow$.

#### Example 20

Consider the TRS $\mathcal {R}$ consisting of following rules:

$$\begin{aligned} \textsf{h}(\textsf{f}(\textsf{g}(\textsf{a}),x,y))&\rightarrow \textsf{g}(\textsf{a})&\textsf{g}(\textsf{f}(x,\textsf{h}(x),y)))&\rightarrow x&\textsf{h}(\textsf{f}(x,y,\textsf{h}(\textsf{a})))&\rightarrow \textsf{h}(x) \end{aligned}$$

We start by collecting the subterms of the left-hand sides:

$$\begin{aligned} T^\bot = \{\bot , \textsf{a}, \textsf{g}(\textsf{a}), \textsf{h}(\bot ), \textsf{h}(\textsf{a}), \textsf{f}(\textsf{g}(\textsf{a}),\bot ,\bot ), \textsf{f}(\bot ,\textsf{h}(\bot ),\bot ), \textsf{f}(\bot ,\bot ,\textsf{h}(\textsf{a}))\} \end{aligned}$$

Closing $T^\bot$ under $\uparrow$ adds the following terms:

$$\begin{aligned} \textsf{f}(\textsf{g}(\textsf{a}),\bot ,\bot ) \,\uparrow \, \textsf{f}(\bot ,\textsf{h}(\bot ),\bot )&\,=\, \textsf{f}(\textsf{g}(\textsf{a}),\textsf{h}(\bot ),\bot ) \\ \textsf{f}(\bot ,\bot ,\textsf{h}(\textsf{a})) \,\uparrow \, \textsf{f}(\bot ,\textsf{h}(\bot ),\bot )&\,=\, \textsf{f}(\bot ,\textsf{h}(\bot ),\textsf{h}(\textsf{a})) \\ \textsf{f}(\textsf{g}(a),\textsf{h}(\bot ),\bot ) \,\uparrow \, \textsf{f}(\bot ,\textsf{h}(\bot ),\textsf{h}(\textsf{a}))&\,=\, \textsf{f}(\textsf{g}(\textsf{a}),\textsf{h}(\bot ),\textsf{h}(\textsf{a})) \end{aligned}$$

#### Lemma 19

The set $T_\uparrow$ is finite. 

#### Proof

If $t \uparrow u$ is defined then $\mathcal {P}\textsf{os}(t \uparrow u) = \mathcal {P}\textsf{os}(t) \cup \mathcal {P}\textsf{os}(u)$. It follows that the positions of terms in $T_\uparrow {\setminus } T^\bot$ are positions of terms in $T^\bot$. Since $T^\bot$ is finite, there are only finitely many such positions. Hence the finiteness of $T_\uparrow$ follows from the finiteness of $\mathcal {F}$. $\square$

Although the above proof is simple enough, we formalized the proof below which is based on a concrete algorithm to compute $T_\uparrow$. Actually, the algorithm presented below is based on a general saturation procedure, which is of independent interest.

#### Definition 16

Let $f:U \times U \rightarrow U$ be a (possibly partial) function and let *S* be a finite subset of *U*. The *closure* $C_f(S)$ is the least extension of *S* with the property that $f(a,b) \in C_f(S)$ whenever $a, b \in C_f(S)$ and *f*(*a*, *b*) is defined.

The following lemma provides a sufficient condition for closures to exist. The proof gives a concrete algorithm to compute the closure.

#### Lemma 20

If *f* is a total, associative, commutative, and idempotent function then $C_f(S)$ exists and is finite. 

#### Proof

If $S = \varnothing$ then $C_f(S) = \varnothing$ and the claim trivially holds. Suppose $S \ne \varnothing$ and let *a* be an arbitrary element in *S*. We show

$$\begin{aligned} C_f(S) \,=\, C_f(S \setminus \{a\}) \cup \{a\} \cup \{f(a,c) \mid c \in C_f(S \setminus \{a\})\} \end{aligned}$$

Since *S* is finite, this gives rise to the following iterative algorithm to compute $C_f(S)$:

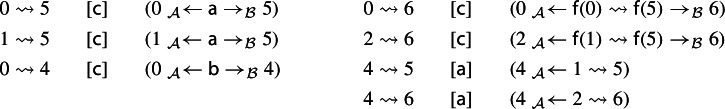

In each iteration only finitely many elements are added. Hence $C_f(S)$ is finite. It remains to show the above equation. The inclusion from left to right is immediate from the definition of $C_f(S)$. Let *b* be an arbitrary element of $C_f(S)$. If $b \in S$ then $b \in C_f(S {\setminus } \{a\}) \cup \{a\}$. If $b \notin S$ then $b = f(a_1,f(a_2,\dots f(a_{n-1},a_n)\dots ))$ for some sequence of elements $a_1,\dotsc ,a_{n} \in S$. If *a* is an element of this sequence then, using the properties of *f*, we may assume *a* appears exactly once in the sequence. Hence $b = f(a,c)$ for some element $c \in C_f(S {\setminus } \{a\})$. If *a* is not an element of $a_1,\dotsc ,a_{n}$ then $b \in C_f(S \setminus \{a\})$. This completes the proof. $\square$

Since our function $\uparrow$ is partial, we need to lift it to a total function that preserves associativity and commutativity. In our abstract setting this entails finding a binary predicate *P* on *U* such that *f*(*a*, *b*) is defined if *P*(*a*, *b*) holds. In addition, the following properties need to be fulfilled:

- *P* is reflexive and symmetric,
- if *P*(*a*, *f*(*b*, *c*)) and *P*(*b*, *c*) hold then *P*(*a*, *b*) and *P*(*f*(*a*, *b*), *c*) hold as well, for all $a, b, c \in U$.

For the details we refer to the formalization.

#### Definition 17

The tree automaton $\mathcal {A}_{\,\textsf{NF}(\mathcal {R})} = (\mathcal {F},Q,Q_f,\Delta )$ is defined as follows: $Q = Q_f = T_\uparrow$ and $\Delta$ consists of all transition rules $f(p_1,\dotsc ,p_{n}) \rightarrow q$ such that $f(p_1,\dotsc ,p_{n})$ is no redex of $\mathcal {R}$ and *q* is the maximal element of *Q* satisfying $q \leqslant f(p_1,\dotsc ,p_{n})$.^3^

#### Example 21

For the TRS $\mathcal {R}$ of Example 20, the tree automaton $\mathcal {A}_{\,\textsf{NF}(\mathcal {R})}$ consists of the following transition rules:

$$\begin{aligned}{} & {} \textsf{a} \rightarrow 1 \qquad \textsf{g}(p) \rightarrow {\left\{ \begin{array}{ll} 2 &{}\text {if }p = 1 \\ 0 &{}\text {if }p \notin \{1, 6, 9, 10\} \end{array}\right. } \qquad \textsf{h}(p) \rightarrow {\left\{ \begin{array}{ll} 4 &{}\text {if }p = 1 \\ 3 &{}\text {if }p \notin \{1, 8, 10\} \end{array}\right. } \\{} & {} \textsf{f}(p,q,r) \rightarrow {\left\{ \begin{array}{ll} 5 &{}\text {if }p = 2, q \notin \{3, 4\} \\ 6 &{}\text {if }p \ne 2, q \in \{3, 4\}, r \ne 4 \\ 7 &{}\text {if }q \notin \{3, 4\}, r = 4 \\ 8 &{}\text {if }p = 2, q \in \{3, 4\}, r \ne 4 \end{array}\right. } \\{} & {} \textsf{f}(p,q,r) \rightarrow {\left\{ \begin{array}{ll} 9 &{}\text {if }p \ne 2, q \in \{3, 4\}, r = 4 \\ 10 &{}\text {if }p = 2, q \in \{3, 4\}, r = 4 \\ 0 &{}\text {otherwise} \end{array}\right. } \end{aligned}$$

Here we use the following abbreviations:

$$\begin{aligned} 0&= \bot&3&= \textsf{h}(\bot )&6&= \textsf{f}(\bot ,\textsf{h}(\bot ),\bot )&8&= \textsf{f}(\textsf{g}(\textsf{a}),\textsf{h}(\bot ),\bot ) \\ 1&= \textsf{a}&4&= \textsf{h}(\textsf{a})&7&= \textsf{f}(\bot ,\bot ,\textsf{h}(\textsf{a}))&9&= \textsf{f}(\bot ,\textsf{h}(\bot ),\textsf{h}(\textsf{a})) \\ 2&= \textsf{g}(\textsf{a})&5&= \textsf{f}(\textsf{g}(\textsf{a}),\bot ,\bot ){} & {} {}&10&= \textsf{f}(\textsf{g}(\textsf{a}),\textsf{h}(\bot ),\textsf{h}(\textsf{a})) \end{aligned}$$

As can be seen from the above example, the tree automaton $\mathcal {A}_{\,\textsf{NF}(\mathcal {R})}$ is not completely defined. Unlike the construction in [6], we do not have an additional state that is reached by all reducible ground terms.

Before proving that $\mathcal {A}_{\,\textsf{NF}(\mathcal {R})}$ accepts the ground normal forms of $\mathcal {R}$, we first show that $\mathcal {A}_{\,\textsf{NF}(\mathcal {R})}$ is well-defined, which amounts to showing that for every $f(p_1,\dotsc ,p_{n})$ with $f \in \mathcal {F}$ and $p_1,\dotsc ,p_{n} \in T_\uparrow$ the set of states *q* such that $q \leqslant f(p_1,\dotsc ,p_{n})$ has a maximum element with respect to the partial order $\leqslant$.

#### Lemma 21

For every term $t \in \mathcal {T}(\mathcal {F}_\bot )$ the set $\{s \in T_\uparrow \mid s \leqslant t\}$ has a unique maximal element. 

#### Proof

Let $S = \{s \in T_\uparrow \mid s \leqslant t\}$. Because $\bot \leqslant t$ and $\bot \in T_\uparrow$, $S \ne \varnothing$. If $s_1, s_2 \in S$ then $s_1 \leqslant t$ and $s_2 \leqslant t$ and thus $s_1 \uparrow s_2$ is defined and satisfies $s_1 \uparrow s_2 \leqslant t$. Since $T_\uparrow$ is closed under $\uparrow$, $s_1 \uparrow s_2 \in T_\uparrow$ and thus $s_1 \uparrow s_2 \in S$. Consequently, *S* has a unique maximal element. $\square$

The next lemma is a trivial consequence of the fact that $\mathcal {A}_{\,\textsf{NF}(\mathcal {R})}$ has no $\varepsilon$-transitions.

#### Lemma 22

The tree automaton $\mathcal {A}_{\,\textsf{NF}(\mathcal {R})}$ is deterministic. 

#### Lemma 23

If $t \in \mathcal {T}(\mathcal {F})$ with $t \rightarrow _{\Delta }^{*} q$ and $s^\bot \leqslant t^\bot$ for a proper subterm *s* of some left-hand side of $\mathcal {R}$ then $s^\bot \leqslant q$. 

#### Proof

We use induction on *t*. Let $t = f(t_1,\dotsc ,t_{n})$. We have $t \rightarrow _{\Delta }^{*} f(q_1,\dotsc ,q_{n}) \rightarrow _{\Delta }q$. We proceed by case analysis on *s*. If *s* is a variable then $s^\bot = \bot$ and, as $\bot$ is minimal in $\leqslant$, we obtain $s^\bot \leqslant q$. Otherwise we must have $\textsf{root}(s) = f$ from the assumption $s^\bot \leqslant t^\bot$. So we may write $s = f(s_1,\dotsc ,s_{n})$. The induction hypothesis yields $s_i^\bot \leqslant q_i$ for all $1 \leqslant i \leqslant n$. Hence $s^\bot = f(s_1^\bot ,\dots ,s_n^\bot ) \leqslant f(q_1,\dotsc ,q_{n})$. Additionally we have $s^\bot \in Q$ by Definition 17 as *s* is a proper subterm of a left-hand side of $\mathcal {R}$. Since $f(q_1,\dotsc ,q_{n}) \rightarrow q$ is a transition rule, we obtain $f(s_1,\dotsc ,s_{n})^\bot \leqslant q$ from the maximality of *q*. $\square$

Using the previous result we can prove that no redex of $\mathcal {R}$ reaches a state in $\mathcal {A}_{\,\textsf{NF}(\mathcal {R})}$.

#### Lemma 24

If $t \in \mathcal {T}(\mathcal {F})$ is a redex then $t \rightarrow _{\Delta }^{*} q$ for no state $q \in T_\uparrow$. 

#### Proof

We have $\ell ^\bot \leqslant t$ for some left-hand side $\ell$ of $\mathcal {R}$. For a proof by contradiction, assume $t \rightarrow _{\Delta }^{*} q$. Write $t = f(t_1,\dotsc ,t_{n})$. We have $t \rightarrow _{\Delta }^{*} f(q_1,\dotsc ,q_{n}) \rightarrow _{\Delta }q$ and obtain $\ell ^\bot \leqslant f(q_1,\dotsc ,q_{n})$ by a case analysis on $\ell$ and Lemma 23. Therefore the transition rule $f(q_1,\dotsc ,q_{n}) \rightarrow _{\Delta }q$ cannot exist by Definition 17. $\square$

#### Lemma 25

If $t \rightarrow _{\Delta }^{*} q$ and $t \in \mathcal {T}(\mathcal {F})$ then $q \leqslant t$. 

#### Proof

We use induction on *t*. Let $t = f(t_1,\dotsc ,t_{n})$. We have $t \rightarrow _{\Delta }^{*} f(q_1,\dotsc ,q_{n}) \rightarrow _{\Delta }^{*} q$. The induction hypothesis yields $q_i \leqslant t_i$ for all $1 \leqslant i \leqslant n$ and thus also $f(q_1,\dotsc ,q_{n}) \leqslant f(t_1,\dotsc ,t_{n})$. We have $q \leqslant f(q_1,\dotsc ,q_{n})$ by Definition 17 and thus $q \leqslant t$ by the transitivity of $\leqslant$. $\square$

#### Lemma 26

If $t \in \textsf{NF}(\mathcal {R})$ then $t \rightarrow _{\Delta }^{*} q$ for some state $q \in T_\uparrow$. 

#### Proof

We use induction on *t*. Let $t = f(t_1,\dotsc ,t_{n})$. Since $t_1,\dotsc ,t_{n} \in \textsf{NF}(\mathcal {R})$ we obtain $f(t_1,\dotsc ,t_{n}) \rightarrow _{\Delta }^{*} f(q_1,\dotsc ,q_{n})$ from the induction hypothesis. Suppose $f(q_1,\dotsc ,q_{n})$ is a redex, so $\ell ^\bot \leqslant f(q_1,\dotsc ,q_{n})$ for some left-hand side $\ell$ of $\mathcal {R}$. From Lemma 25 we obtain $q_i \leqslant t_i$ for all $1 \leqslant i \leqslant n$ and thus $f(q_1,\dotsc ,q_{n}) \leqslant f(t_1,\dotsc ,t_{n})$. Hence $\ell ^\bot \leqslant f(t_1,\dotsc ,t_{n})$. This however contradicts the assumption that *t* is a normal form. (Here we need left-linearity of $\mathcal {R}$.) Therefore $f(q_1,\dotsc ,q_{n})$ is no redex and thus, using Lemma 21, there exists a transition $f(q_1,\dotsc ,q_{n}) \rightarrow q$ in $\Delta$ and thus $t \rightarrow _{\Delta }^{*} q$. $\square$

#### Theorem 15

($T~\mathsf {::=}~\textsf{NF}$)  If $\mathcal {R}$ is a left-linear TRS then $L(\mathcal {A}_{\,\textsf{NF}(\mathcal {R})}) = \textsf{NF}(\mathcal {R})$.

#### Proof

Let $t \in \mathcal {T}(\mathcal {F})$. If $t \in \textsf{NF}(\mathcal {R})$ then $t \rightarrow _{\Delta }^{*} q$ for some state $q \in T_\uparrow$ by Lemma 26. Since all states in $T_\uparrow$ are final, $t \in L(\mathcal {A}_{\,\textsf{NF}(\mathcal {R})})$. Next assume $t \notin \textsf{NF}(\mathcal {R})$. Hence $t = C[s]$ for some redex *s*. According to Lemma 24*s* does not reach a state in $\mathcal {A}_{\,\textsf{NF}(\mathcal {R})}$. Hence also *t* cannot reach a state and thus $t \notin L(\mathcal {A}_{\,\textsf{NF}(\mathcal {R})})$. $\square$

### Decision Procedure

In Table  we summarize the effective closure properties that were presented in detail in this section and formalized in Isabelle. The asterisks indicate that for anchored GTTs we have two closure properties each. The underlined result (the closure of $\textsf{RR}_{2}$ relations under composition) is not used in the decision procedure but does hold: If $R_1$ and $R_2$ are $\textsf{RR}_{2}$ relations then $R_1 \mathrel {\circ } R_2 = \Pi _2(C_3(R_1) \cap C_1(R_1))$. Concerning the empty entry in the table, it can be shown that GTT relations are closed under the context operation $(\cdot )^{n}_{p}$ if and only if $n \in \{{\geqslant }, 1, {>}\}$ and $p \in \{{\geqslant }, \varepsilon \}$. The second and third columns in the left part of Table 1 correspond to the *A* and *R* parts of the grammar in Fig. 1.

**Table 1:** Summary of (formalized) closure properties

Operation	GTTs	Anchored GTTs	$\textsf{RR}_{2}$	Operation	Regular relations
Union	$\times$	$\checkmark$	$\checkmark$	Union	$\checkmark$
Intersection	$\times$	$\checkmark$	$\checkmark$	Intersection	$\checkmark$
Complement	$\times$	$\checkmark$	$\checkmark$	Complement	$\checkmark$
Composition	$\checkmark$	$\checkmark$ ${}^*$	$\underline{\checkmark }$	Projection	$\checkmark$
Inverse	$\checkmark$	$\checkmark$	$\checkmark$	Cylindrification	$\checkmark$
Transitive closure	$\checkmark$	$\checkmark$ ${}^*$	$\times$	Permutation	$\checkmark$
Context closure		$\times$	$\checkmark$		

The logical structure of formulas in the first-order theory of rewriting is taken care of by the closure operations on regular relations listed in the second half of Table 1.

In Table  we show how some of the common binary predicates in term rewriting are represented as $\textsf{RR}_{2}$ relations using the corresponding operations. These are added to the language $\mathcal {L}$ of the first-order theory of rewriting without compromising the decidability result that is presented below.

**Table 2:** Binary predicates as $\textsf{RR}_{2}$ relations

$\rightarrow$ = $(\rightarrow _{\varepsilon })^1_\geqslant$	$\leftarrow$ = $((\rightarrow _{\varepsilon })^1_\geqslant )^-$
$\rightarrow _{\varepsilon }$ = $(\rightarrow _{\varepsilon })^1_\varepsilon$	$\rightarrow ^{+}$ =
$\rightarrow _{>\varepsilon }$ = $(\rightarrow _{\varepsilon })^1_>$	$\rightarrow _{>\varepsilon }^{*}$ =
= $(\rightarrow _{\varepsilon })^\geqslant _\geqslant$	$\rightarrow ^{*}$ =
$\rightarrow _{\varepsilon }^{+}$ = $((\rightarrow _{\varepsilon })^+)^1_\varepsilon$	$\leftrightarrow ^{*}$ =
$\leftrightarrow$ = $({(\rightarrow _{\varepsilon })^-} \,\cup \, {\rightarrow _{\varepsilon }})^1_\geqslant$	$\downarrow$ =
$\rightarrow ^{!}$ =	

#### Theorem 16

The first-order theory of rewriting is decidable for finite linear variable-separated TRSs.

#### Proof

Let $\varphi (x_1,\dotsc ,x_{n})$ be a first-order formula over the language $\mathcal {L}$ with free variables $x_1,\dotsc ,x_{n}$. Let $\mathcal {R}$ be a finite linear variable-separated TRS over a signature $\mathcal {F}$. We construct an $\textsf{RR}_{n}$ automaton that accepts the encoding of the relation $[\![\varphi ]\!] = \{(t_1,\dotsc ,t_{n}) \mid \mathcal {R}\vDash \varphi (t_1,\dotsc ,t_{n})\}$. For closed formulas, checking $\mathcal {R}\vDash \varphi$ then boils down to checking non-emptiness of $\langle {[\![}\varphi {]\!]}\rangle$, which is decidable. We prove the (correctness of the) construction by structural induction on $\varphi$. In the base case $\varphi$ is an atomic formula and we distinguish the following cases.

1. If $\varphi = (x \rightarrow y)$ then we use Theorem 4 to obtain an anchored GTT for $\rightarrow _{\varepsilon }$, which is transformed into an $\textsf{RR}_{2}$ automaton for $\langle \rightarrow _{\varepsilon }\rangle$ by Theorem 10. An application of Theorem 11 with $n = 1$ and $p = {\geqslant }$ yields an $\textsf{RR}_{2}$ automaton for $\langle (\rightarrow _{\varepsilon })^1_\geqslant \rangle = [\![\varphi ]\!]$.
2. If $\varphi = (x \rightarrow ^{*} y)$ then we repeat the constructions in the previous case, with an additional application of modified transitive closure (Theorem 8) before Theorem 11 (with $n = p = {\geqslant }$) is applied.
3. If $\varphi = (x = y)$ then $[\![\varphi ]\!]$ is regular by Lemma 18.

Here we assume that $x \ne y$. If *x* and *y* are the same variable then $[\![\varphi ]\!]$ is a set of ground terms and the above constructions need to be modified as follows. If $\varphi = (x = x)$ then $\langle {[\![}\varphi {]\!]}\rangle = \{\langle t\rangle \mid t \in \mathcal {T}(\mathcal {F})\} = \mathcal {T}(\mathcal {F})$ is accepted by the tree automaton $(\mathcal {F},\{q\},\{q\},\Delta )$ with $\Delta$ consisting of all rules $f(q,\dotsc ,q) \rightarrow q$ for $f \in \mathcal {F}$. Consider $\varphi = (x \rightarrow x)$. We have $\{\langle t,t\rangle \mid t \rightarrow _{\mathcal {R}}t\} = \{\langle t,u\rangle \mid t \rightarrow _{\mathcal {R}}u \text { and }t = u\}$. The latter is regular (cases 1 and 3 above together with Theorem 12) and hence the regularity of $\langle {[\![}\varphi {]\!]}\rangle = \{\langle t\rangle \mid t \rightarrow _{\mathcal {R}}t\}$ follows by an application of Theorem 2. In the remaining case ($\varphi = (x \rightarrow ^{*} x)$) we reason as in the previous case (using cases 2 and 3 above). Next we consider the propositional connectives.

4. Suppose $\varphi = \lnot \psi$. The induction hypothesis yields an $\textsf{RR}_{n}$ automaton that accepts $\langle {[\![}\psi {]\!]}\rangle$. Since the class of *n*-ary regular relations is effectively closed under complement (Theorem 13), we obtain an $\textsf{RR}_{n}$ automaton that accepts $\langle {[\![}\varphi {]\!]}\rangle$.
5. Suppose $\varphi = \psi _1 \wedge \psi _2$. Since $\psi _1$ and $\psi _2$ may have less free variables than $\varphi$, we cannot use Theorem 12 without further ado. Let $y_1,\dotsc ,y_{k}$ be the free variables in $\psi _1$ and $z_1,\dotsc ,z_{m}$ be the free variables in $\psi _2$. We have $\{x_1,\dotsc ,x_{n}\} = \{y_1,\dotsc ,y_{k}\} \cup \{z_1,\dotsc ,z_{m}\}$. Because regular relations are closed under permutation (Theorem 14), we may assume that the variables in $y_1,\dotsc ,y_{k}$ and $z_1,\dotsc ,z_{m}$ are listed in the same order as in $x_1,\dotsc ,x_{n}$. The induction hypothesis yields an $\textsf{RR}_{k}$ automaton $\mathcal {A}_1$ for $\langle {[\![}\psi _1{]\!]}\rangle$ and an $\textsf{RR}_{m}$ automaton $\mathcal {A}_2$ for $\langle {[\![}\psi _2{]\!]}\rangle$. Using $2n - (k + m)$ applications of cylindrification (Theorem 14), these automata are turned into $\textsf{RR}_{n}$ automata. Since *n*-ary regular relations are closed under intersection (Theorem 12), we obtain an $\textsf{RR}_{n}$ automaton for $\langle {[\![}\varphi {]\!]}\rangle$.
6. The other binary connectives are handled exactly like conjunction.

The final cases involve the two quantifiers.

7. Suppose $\varphi = \exists \,x\,\psi$. If *x* does not occur free in $\psi$ then $\langle {[\![}\varphi {]\!]}\rangle = \langle {[\![}\psi {]\!]}\rangle$ and hence the result follows immediately from the induction hypothesis. So we assume that *x* occurs free in $\psi$ and $n \geqslant 0$. The induction hypothesis yields an $\textsf{RR}_{n+1}$ automaton that accepts $\langle {[\![}\psi {]\!]}\rangle$. Since the class of regular relations is effectively closed under projection (Theorem 2), we obtain an $\textsf{RR}_{n}$ automation that accepts $\langle {[\![}\varphi {]\!]}\rangle$.
8. The case $\varphi = \forall \,x\,\psi$ reduces to the preceding case by the well-known equivalence $\forall \,x\,\psi \equiv \lnot \,\exists \,x\,\lnot \,\psi$. $\square$

## Properties on Non-ground Terms

Since tree automata operate on ground terms, the decision procedure presented in the preceding section is restricted to properties on ground terms. The following example shows that ground-confluence, i.e., confluence restricted to ground terms, is not the same as confluence.

### Example 22

The left-linear right-ground TRS $\mathcal {R}$ consisting of the rules

$$\begin{aligned} \textsf{a}&\rightarrow \textsf{b}&\textsf{f}(\textsf{a},x)&\rightarrow \textsf{b}&\textsf{f}(\textsf{b},\textsf{b})&\rightarrow \textsf{b} \end{aligned}$$

over the signature $\mathcal {F}= \{\textsf{a},\textsf{b},\textsf{f}\}$ is ground-confluent because every ground term in $\mathcal {T}(\mathcal {F})$ rewrites to $\textsf{b}$. Confluence does not hold; the term $\textsf{f}(\textsf{a},x)$ rewrites to the different normal forms $\textsf{b}$ and $\textsf{f}(\textsf{b},x)$.

In this section we present results that allow the use of FORT on (certain) properties over arbitrary terms. The main idea is to extend the given signature $\mathcal {F}$ with constants to replace variables in terms. The required number of additional constants depends on the property under consideration. We consider the following confluence-related properties: 
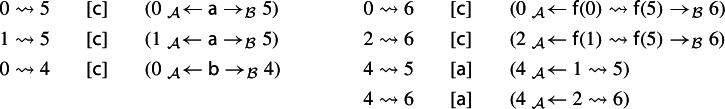


Here $t \downarrow u$ denotes joinability: $\exists v~(t \rightarrow _{}^{*} v \wedge u \rightarrow _{}^{*} v)$. Let $\mathcal {P}_1$ be the collection of these properties. We also consider the following properties involving two TRSs $\mathcal {R}$ and $\mathcal {S}$: 
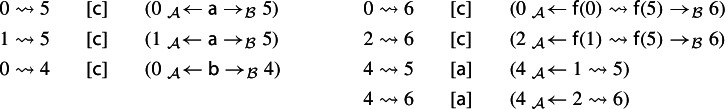


Let $\mathcal {P}_2 = \{\textsf{COM},\textsf{CE},\textsf{NE}\}$. For a property $P \in \mathcal {P}_1 \cup \mathcal {P}_2$, $\textsf{G}P$ denotes the property *P* restricted to ground terms. The diagram in Fig.  summarizes the relationships between properties *P* and $\textsf{G}P$ for $P \in \mathcal {P}_1$. The properties $\textsf{CE}, \textsf{NE} \in \mathcal {P}_2$ are unrelated.

**Fig. 6:**
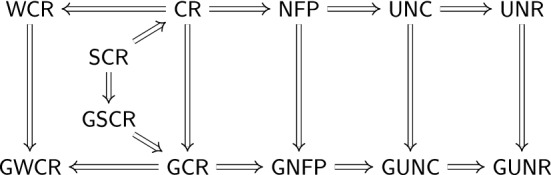
Confluence-related properties on ground and non-ground terms

According to the following result, all considered properties are closed under signature extension.

### Lemma 27

Let $\mathcal {R}$ and $\mathcal {S}$ be linear variable-separated TRSs over a common signature $\mathcal {F}$.

1. If $P \in \mathcal {P}_1$ and $(\mathcal {F},\mathcal {R}) \vDash P$ then $(\mathcal {F}\uplus \{c\},\mathcal {R}) \vDash P$.
2. If $P \in \mathcal {P}_2$ and $(\mathcal {F},\mathcal {R},\mathcal {S}) \vDash P$ then $(\mathcal {F}\uplus \{c\},\mathcal {R},\mathcal {S}) \vDash P$.

### Proof

Let $\mathcal {U}$ be a linear variable-separated TRS not containing the constant *c*. For any $x \in \mathcal {V}$, the mapping $\phi _c^x:\mathcal {T}(\mathcal {F}\uplus \{c\},\mathcal {V}) \rightarrow \mathcal {T}(\mathcal {F},\mathcal {V})$ replaces all occurrences of *c* in terms by the variable *x*:

$$\begin{aligned} \phi _c^x(t)&= {\left\{ \begin{array}{ll} x &{}\text {if }t = c \\ t &{}\text {if }t \in \mathcal {V}\\ f(\phi _c^x(t_1),\dots ,\phi _c^x(t_n)) &{}\text {if }t = f(t_1,\dotsc ,t_{n}) \end{array}\right. } \end{aligned}$$

A straightforward induction proof reveals that $\phi _c^x(s) \rightarrow _{\mathcal {U}}^{*} \phi _c^x(t)$ whenever $s \rightarrow _{\mathcal {U}}^{*} t$. By choosing $x \notin \mathcal {V}\textsf{ar}(s) \cup \mathcal {V}\textsf{ar}(t)$, the reverse direction holds as well. Moreover, since linear variable-separated TRSs are closed under rule inversion, the equivalence also holds for ${\leftrightarrow _{\mathcal {U}}^{*}} = {\rightarrow _{\mathcal {U}\cup \mathcal {U}^-}^{*}}$. The lemma is an easy consequence of these facts. We illustrate this for $\textsf{COM}$. Given $s \rightarrow _{\mathcal {R}}^{*} t$ and $s \rightarrow _{\mathcal {S}}^{*} u$, with $s, t, u \in \mathcal {T}(\mathcal {F}\uplus \{c\},\mathcal {V})$, we obtain $\phi _c^x(s) \rightarrow _{\mathcal {R}}^{*} \phi _c^x(t)$ and $\phi _c^x(s) \rightarrow _{\mathcal {S}}^{*} \phi _c^x(u)$. Commutation of $(\mathcal {F},\mathcal {R},\mathcal {S})$ yields a term $v \in \mathcal {T}(\mathcal {F},\mathcal {V})$ such that $\phi _c^x(t) \rightarrow _{\mathcal {S}}^{*} v$ and $\phi _c^x(u) \rightarrow _{\mathcal {R}}^{*} v$. By taking $x \notin \mathcal {V}\textsf{ar}(t) \cup \mathcal {V}\textsf{ar}(u)$, we obtain $t \rightarrow _{\mathcal {S}}^{*} v'$ and $u \rightarrow _{\mathcal {R}}^{*} v'$ for $v' = v\{x \mapsto c\}$ by closure of rewriting under substitutions. 

So adding constants preserves the properties of interest. For removing constants more effort is required. For the properties in $\mathcal {P}_1 \cup \mathcal {P}_2$, root steps will play a major role. Root steps are important since they permit the use of different substitutions for the left and right-hand side of the employed rewrite rule, due to variable separation. We therefore start with a preliminary result (Lemma 28) which provides abstract conditions that permit the restriction to rewrite sequences containing root steps. We write $\rightarrow _{\mathcal {R}}^{*\varepsilon *}$ for the relation $\rightarrow _{\mathcal {R}}^{*} \cdot \rightarrow _{\mathcal {R}}^{\varepsilon } \cdot \rightarrow _{\mathcal {R}}^{*}$. The proof of Lemma 28 is obtained by a straightforward induction on the term structure and the multi-hole context closure of the rewrite relation, and is omitted.

### Definition 18

A binary predicate *P* on terms over a given signature $\mathcal {F}$ is closed under *multi-hole contexts* if $P(C[s_1,\dotsc ,s_{n}], C[t_1,\dotsc ,t_{n}])$ holds whenever *C* is a multi-hole context over $\mathcal {F}$ with $n \geqslant 0$ holes and $P(s_i,t_i)$ holds for all $1 \leqslant i \leqslant n$. 

### Lemma 28

Let $\mathcal {A}$ and $\mathcal {B}$ be TRSs over the same signature $\mathcal {F}$ and let *P* be a binary predicate that is closed under multi-hole contexts over $\mathcal {F}$.

1. If $s \rightarrow _{\mathcal {A}}^{*\varepsilon *} t \implies P(s,t)$ for all terms *s* and *t* then $s \rightarrow _{\mathcal {A}}^{*} t \implies P(s,t)$ for all terms *s* and *t*.
2. If $s \rightarrow _{\mathcal {A}}^{*\varepsilon *} \cdot \rightarrow _{\mathcal {B}}^{*} t ~\vee ~ s \rightarrow _{\mathcal {A}}^{*} \cdot \rightarrow _{\mathcal {B}}^{*\varepsilon *} t \implies P(s,t)$ for all terms *s* and *t* then $s \rightarrow _{\mathcal {A}}^{*} \cdot \rightarrow _{\mathcal {B}}^{*} t \implies P(s,t)$ for all terms *s* and *t*.

For example, in the results below (Lemmata 34 and 35) for $\textsf{NFP}$ we make use of this lemma by instantiating part 2 with $P_1(s,t):\textsf{NF}(t) \implies s \rightarrow _{\mathcal {R}}^{*} t$, $\mathcal {R}^-$ for $\mathcal {A}$, and $\mathcal {R}$ for *B*. This results in the statement that if

$$\begin{aligned} s \rightarrow _{\mathcal {R}^-}^{*\varepsilon *} \cdot \rightarrow _{\mathcal {R}}^{*} t ~\vee ~ s \rightarrow _{\mathcal {R}^-}^{*} \cdot \rightarrow _{\mathcal {R}}^{*\varepsilon *} t&\implies \textsf{NF}(t) \implies s \rightarrow _{\mathcal {R}}^{*} t \end{aligned}$$

then

$$\begin{aligned} s \rightarrow _{\mathcal {R}^-}^{*} \cdot \rightarrow _{\mathcal {R}}^{*} t&\implies \textsf{NF}(t) \implies s \rightarrow _{\mathcal {R}}^{*} t \end{aligned}$$

Using the identity  and the definition of $\textsf{NFP}$, it follows that $\textsf{NFP}$ is a consequence of the statementfor all $s, t \in \mathcal {T}(\mathcal {F})$. Hence we only need to consider rewrite sequences involving root steps, which together with variable separation significantly simplifies the proof. For the other properties of interest, Lemma 28 is instantiated as follows.

- For $\textsf{UNC}$ we use part 1 with $P_2(s,t):\textsf{NF}(s) \wedge \textsf{NF}(t) \implies s = t$ and $\mathcal {R}\cup \mathcal {R}^-$ for $\mathcal {A}$.
- For $\textsf{UNR}$ we use part 2 with the same predicate $P_2$ and $\mathcal {R}^{-}$ for $\mathcal {A}$ and $\mathcal {R}$ for $\mathcal {B}$.
- For $\textsf{COM}$ we use part 2 with $P_3(s,t):s \rightarrow _{\mathcal {S}}^{*} \cdot \rightarrow _{\mathcal {R}^-}^{*} t$ and $\mathcal {R}^{-}$ for $\mathcal {A}$ and $\mathcal {S}$ for $\mathcal {B}$.
- For $\textsf{CR}$ we use part 2 with the same predicate $P_3$ and replace $\mathcal {S}$ by $\mathcal {R}$.
- For $\textsf{NE}$ we use part 1 twice, with $P_4(s,t):\textsf{NF}_\mathcal {R}(t) \implies s \rightarrow _{\mathcal {S}}^{*} t$ and $\mathcal {R}$ for $\mathcal {A}$, and with $P_5(s,t):\textsf{NF}_\mathcal {S}(t) \implies s \rightarrow _{\mathcal {R}}^{*} t$ and $\mathcal {S}$ for $\mathcal {A}$.
- For $\textsf{CE}$ we use part 1 twice, with $P_6(s,t):s \rightarrow _{\mathcal {S}\cup \mathcal {S}^-}^{*} t$ and $\mathcal {R}\cup \mathcal {R}^-$ for $\mathcal {A}$, and with $P_7(s,t):s \rightarrow _{\mathcal {R}\cup \mathcal {R}^-}^{*} t$ and $\mathcal {S}\cup \mathcal {S}^-$ for $\mathcal {A}$.

In addition, we make use of the identities ${\rightarrow _{\mathcal {R}\cup \mathcal {R}^-}^{*\varepsilon *}} = {\leftrightarrow _{\mathcal {R}}^{*\varepsilon *}}$ and ${\rightarrow _{\mathcal {R}\cup \mathcal {R}^-}^{*}} = {\leftrightarrow _{\mathcal {R}}^{*}}$ for $\textsf{UNC}$ and $\textsf{CE}$.

### Lemma 29

The properties $P_1, \dots , P_7$ are closed under multi-hole contexts. 

Strong confluence ($\textsf{SCR}$) and local confluence ($\textsf{WCR}$) cannot be reduced to root steps with Lemma 28, because they involve single steps in their definition, which are not multi-hole context closed. However, by investigating the positions involved in $s \rightarrow t$ and $s \rightarrow u$ we easily deduce a reduction to root steps for both properties.

### Lemma 30

A TRS is local confluent if and only if

$$\begin{aligned} s \rightarrow ^{\varepsilon } t \wedge s \rightarrow u&~\implies ~ t \downarrow u \end{aligned}$$

for all terms *s*, *t* and *u*. A TRS is strongly confluent if and only iffor all terms *s*, *t* and *u*. 

The next lemma is a key result. It allows the removal of introduced fresh constants while preserving the reachability relation. Note that variable-separation is not required.

### Lemma 31

Let $\mathcal {R}$ be a linear TRS over a signature $\mathcal {F}$ that contains a constant *c* which does not appear in $\mathcal {R}$. If $s \rightarrow _{\mathcal {R}}^{*} t$ with $c \in \mathcal {F}\textsf{un}(s) {\setminus } \mathcal {F}\textsf{un}(t)$ then $s[u]_p \rightarrow _{\mathcal {R}}^{*} t$ using the same rewrite rules at the same positions, for all terms *u* and positions $p \in \mathcal {P}\textsf{os}(s)$ such that $s|_p = c$. 

The restriction to linear TRSs can also be lifted, at the expense of a more complicated replacement function and proof. Since the decision procedure implemented in FORT-h relies on linearity and variable-separation, we present a simple proof for linear TRSs. Due to calculations involving positions, the formalization in Isabelle/HOL was anything but simple.

### Proof

We use induction on the length of $s \rightarrow _{\mathcal {R}}^{*} t$. If this length is zero then there is nothing to show as $\mathcal {F}\textsf{un}(s) {\setminus } \mathcal {F}\textsf{un}(t) = \varnothing$. Suppose $s \rightarrow _{\mathcal {R}}v \rightarrow _{\mathcal {R}}^{*} t$ and write $s = C[\ell \sigma ] \rightarrow _{\mathcal {R}}C[r\sigma ] = v$. Let $p'$ be the position of the hole in *C* and let $p \in \mathcal {P}\textsf{os}(s)$ with $s|_p = c$. We distinguish two cases.

If $p' \parallel p$ then $s[u]_p = (C[u]_p)[\ell \sigma ]_{p'} \rightarrow _{\mathcal {R}}v'$ with $v' = (C[u]_p)[r\sigma ]_{p'}$. Since $v|_p = C|_p = c$ we can apply the induction hypothesis to $v \rightarrow _{\mathcal {R}}^{*} t$. This yields $v' \rightarrow _{\mathcal {R}}^{*} t$ and hence $s[u]_p \rightarrow _{\mathcal {R}}^{*} t$ as desired.

In the remaining case, $p' \leqslant p$. From $s|_p = c$ and the fact that *c* does not appear in $\mathcal {R}$ we infer that there exists a variable $y \in \mathcal {V}\textsf{ar}(\ell )$ such that $c \in \mathcal {F}\textsf{un}(\sigma (y))$. Let *q* be the (unique) position of *y* in $\ell$ and consider the substitution

$$\begin{aligned} \tau (x)&= {\left\{ \begin{array}{ll} \sigma (y)[u]_{q'} &{}\text {if }x = y \\ \sigma (x) &{}\text {otherwise} \end{array}\right. } \end{aligned}$$

Here $q' = p \backslash (p'q)$ is the position of *c* in $\sigma (y)$. If $y \notin \mathcal {V}\textsf{ar}(r)$ then $v = C[r\sigma ] = C[r\tau ]$ and thus $s[u]_p = C[\ell \tau ] \rightarrow _{\mathcal {R}}C[r\tau ] = v \rightarrow _{\mathcal {R}}^{*} t$. If $y \in \mathcal {V}\textsf{ar}(r)$ then there exists a unique position $q'' \in \mathcal {P}\textsf{os}(r)$ such that $r|_{q''} = y$. So $v|_{p'q''q'} = c$ and we obtain $s[u]_p = C[\ell \tau ] \rightarrow _{\mathcal {R}}C[r\tau ] = v[u]_{p'q''q'} \rightarrow _{\mathcal {R}}^{*} t$ from the induction hypothesis. $\square$

In the proofs below Lemma 31 (also for $\mathcal {R}^-$) is used as follows. Let $\sigma _c$ denote the substitution mapping all variables to *c*. If $s \sigma _c \rightarrow _{\mathcal {R}}^{*} t$ then $s \rightarrow _{\mathcal {R}}^{*} t$ by repeated applications of Lemma 31 (if the conditions are satisfied).

We now prove that two fresh constants are sufficient to reduce commutation ($\textsf{COM}$), confluence ($\textsf{CR}$), local confluence ($\textsf{WCR}$), unique normal forms ($\textsf{UNC}$ and $\textsf{UNR}$), and the normal form property ($\textsf{NFP}$) to the corresponding ground properties.

### Lemma 32

Linear variable-separated TRSs $\mathcal {R}$ and $\mathcal {S}$ over a common signature $\mathcal {F}$ commute if and only if $\mathcal {R}$ and $\mathcal {S}$ ground-commute over $\mathcal {F}\uplus \{c,d\}$. 

### Proof

The only-if direction follows from Lemma 27. For the if direction suppose $\mathcal {R}$ and $\mathcal {S}$ ground-commute on terms in $\mathcal {T}(\mathcal {F}\uplus \{c,d\})$. In order to conclude that $\mathcal {R}$ and $\mathcal {S}$ commute on terms in $\mathcal {T}(\mathcal {F},\mathcal {V})$, according to Lemma 28, it suffices to show the inclusions

$$\begin{aligned} {\rightarrow _{\mathcal {R}^-}^{*\varepsilon *} \cdot \rightarrow _{\mathcal {S}}^{*}}&~\subseteq ~ {\rightarrow _{\mathcal {S}}^{*} \cdot \rightarrow _{\mathcal {R}^-}^{*}}&{\rightarrow _{\mathcal {R}^-}^{*} \cdot \rightarrow _{\mathcal {S}}^{*\varepsilon *}}&~\subseteq ~ {\rightarrow _{\mathcal {S}}^{*} \cdot \rightarrow _{\mathcal {R}^-}^{*}} \end{aligned}$$

on terms in $\mathcal {T}(\mathcal {F},\mathcal {V})$. Suppose $s \rightarrow _{\mathcal {R}^-}^{*\varepsilon *} \cdot \rightarrow _{\mathcal {S}}^{*} t$. Let the substitution $\sigma _c$ map all variables to *c* and let $\sigma _d$ map all variables to *d*. Since rewriting is closed under substitutions and the variable-separated rule used in the root step $\rightarrow _{\mathcal {R}^-}^{\varepsilon }$ allows changing the substitution, we obtain $s\sigma _c \rightarrow _{\mathcal {R}^-}^{*\varepsilon *} \cdot \rightarrow _{\mathcal {S}}^{*} t\sigma _d$. From ground commutation we obtain $s\sigma _c \rightarrow _{\mathcal {S}}^{*} \cdot \rightarrow _{\mathcal {R}^-}^{*} t\sigma _d$. Note that *s* and *t* are terms in $\mathcal {T}(\mathcal {F},\mathcal {V})$ and hence do not contain the constants *c* and *d*. Therefore, $d \notin \mathcal {F}\textsf{un}(s\sigma _c)$ and $c \notin \mathcal {F}\textsf{un}(t\sigma _d)$. As a consequence, repeated applications of Lemma 31 transform $s\sigma _c \rightarrow _{\mathcal {S}}^{*} \cdot \rightarrow _{\mathcal {R}^-}^{*} t\sigma _d$ into a sequence $s \rightarrow _{\mathcal {S}}^{*} \cdot \rightarrow _{\mathcal {R}^-}^{*} t$ in which *c* and *d* do not appear, proving the first inclusion. Note that in our setting TRSs are closed under rule reversal. Hence we can apply Lemma 31 in both directions, which allows us to remove the constant *d* from the term *t*. The second inclusion ${\rightarrow _{\mathcal {R}^-}^{*} \cdot \rightarrow _{\mathcal {S}}^{*\varepsilon *}} ~\subseteq ~ {\rightarrow _{\mathcal {S}}^{*} \cdot \rightarrow _{\mathcal {R}^-}^{*}}$ is obtained in the same way. $\square$

If the TRSs $\mathcal {R}$ and $\mathcal {S}$ are left-linear right-ground (as opposed to linear variable-separated) then the term *t* in the above proof is ground due to the root step involved. Hence $t\sigma _d = t$, which allows us to simplify the proof and strengthen the statement to use only one additional constant.

### Lemma 33

Left-linear right-ground TRSs $\mathcal {R}$ and $\mathcal {S}$ over a common signature $\mathcal {F}$ commute if and only if $\mathcal {R}$ and $\mathcal {S}$ ground-commute over $\mathcal {F}\uplus \{c\}$. 

The proof for confluence follows directly from commutation. The proofs for the other properties in $\mathcal {P}_1$ are obtained in a similar manner. We present the proof details for strong confluence since it requires a bit more effort.

### Lemma 34

Let $\mathcal {R}$ be a linear variable-separated TRS over a signature $\mathcal {F}$. If $P \in \mathcal {P}_1$ then 
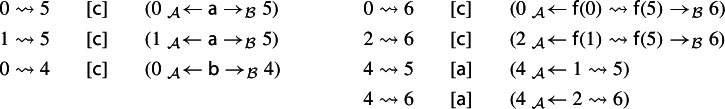


### Proof

We present the if direction for $P = \textsf{SCR}$. First we use Lemma 30 to reduce the problem to local peaks involving a root step. Following the reasoning in the proof of Lemma 32, we obtain a witness *v* such that . If $t\sigma _d = v$ then $u\sigma _c \rightarrow _{\mathcal {R}}^{*} t\sigma _d$ and we obtain $u \rightarrow _{\mathcal {R}}^{*} t$ with the help of Lemma 31. So assume $u\sigma _c \rightarrow _{\mathcal {R}}^{*} \cdot \rightarrow _{\mathcal {R}^-} t\sigma _d$. Using Lemma 31 and induction on the number of variables in *u* we deduce $u \rightarrow _{\mathcal {R}}^{*} \cdot \rightarrow _{\mathcal {R}^-} t\sigma _d$. The same argument applied to *t* produces $u \rightarrow _{\mathcal {R}}^{*} w \rightarrow _{\mathcal {R}^-} t$. Note that *w* may contain occurrences of the constants *c* and *d* since $\mathcal {R}$ is a variable-separated TRS. We use the map defined in the proof of Lemma 27 to eliminate these: $u = \phi _c^x(\phi _d^x(u)) \rightarrow _{\mathcal {R}}^{*} \phi _c^x(\phi _d^x(w)) \rightarrow _{\mathcal {R}^-} \phi _c^x(\phi _d^x(t)) = t$. $\square$

### Lemma 35

Let $\mathcal {R}$ be a left-linear right-ground TRS over a signature $\mathcal {F}$. If $P \in \mathcal {P}_1 \setminus \{\textsf{UNC}\}$ then

$$\begin{aligned} (\mathcal {F},\mathcal {R}) \vDash P&~\iff ~ (\mathcal {F}\uplus \{c\},\mathcal {R}) \vDash \textsf{G}P \end{aligned}$$

Moreover, 
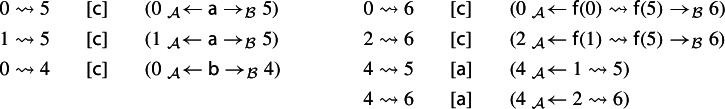


The simplification in the proof of Lemma 32 for left-linear right-ground systems is not applicable for $\textsf{UNC}$ as conversion can introduce variables. The following example shows that adding a single fresh constant is indeed insufficient for $\textsf{UNC}$.

### Example 23

The left-linear right-ground TRS $\mathcal {R}$ consisting of the rules

$$\begin{aligned} \textsf{a}&\rightarrow \textsf{b}&\textsf{f}(x,\textsf{a})&\rightarrow \textsf{f}(\textsf{b},\textsf{b})&\textsf{f}(\textsf{b},x)&\rightarrow \textsf{f}(\textsf{b},\textsf{b})&\textsf{f}(\textsf{f}(x,y),z)&\rightarrow \textsf{f}(\textsf{b},\textsf{b}) \end{aligned}$$

does not satisfy $\textsf{UNC}$ since $\textsf{f}(x,\textsf{b}) \leftarrow \textsf{f}(x,\textsf{a}) \rightarrow \textsf{f}(\textsf{b},\textsf{b}) \leftarrow \textsf{f}(y,\textsf{a}) \rightarrow \textsf{f}(y,\textsf{b})$ is a conversion between distinct normal forms. Adding a single fresh constant $\textsf{c}$ is not enough to violate $\textsf{GUNC}$ as the last two rewrite rules ensure that $\textsf{f}(\textsf{c},\textsf{b})$ is the only ground instance of $\textsf{f}(x,\textsf{b})$ that is a normal form. Adding another fresh constant $\textsf{d}$, $\textsf{GUNC}$ is lost: $\textsf{f}(\textsf{c},\textsf{b}) \leftarrow \textsf{f}(\textsf{c},\textsf{a}) \rightarrow \textsf{f}(\textsf{b},\textsf{b}) \leftarrow \textsf{f}(\textsf{d},\textsf{a}) \rightarrow \textsf{f}(\textsf{d},\textsf{b})$.

The following example shows that at least two fresh constants are required to reduce confluence to ground-confluence for linear variable-separated TRSs.

### Example 24

Consider the linear variable-separated TRS $\mathcal {R}$ consisting of the single rule $\textsf{a} \rightarrow x$ over the signature $\mathcal {F}= \{\textsf{a}\}$. Since  with distinct variables *x* and *y*, $\mathcal {R}$ is not confluent. Ground-confluence holds trivially as $\textsf{a} \rightarrow _{\mathcal {R}}\textsf{a}$ is the only rewrite step between ground terms. Adding a single fresh constant $\textsf{b}$ does not destroy ground-confluence ($\textsf{a} \rightarrow _{\mathcal {R}}\textsf{a}$ and $\textsf{a} \rightarrow _{\mathcal {R}}\textsf{b}$ are the only steps). By adding a second fresh constant $\textsf{c}$, ground-confluence is lost: .

We now turn our attention to the equivalence properties ($\textsf{CE}$ and $\textsf{NE}$) in $\mathcal {P}_2$. For conversion equivalence a single fresh constant suffices to reduce it to ground conversion equivalence.

### Lemma 36

Linear variable-separated TRSs $\mathcal {R}$ and $\mathcal {S}$ over a common signature $\mathcal {F}$ such that $\mathcal {T}(\mathcal {F})\ne \varnothing$ are conversion equivalent if and only if $\mathcal {R}$ and $\mathcal {S}$ are ground conversion equivalent over $\mathcal {F}\uplus \{c\}$. 

### Proof

For the if direction we assume that $\mathcal {R}$ and $\mathcal {S}$ are ground conversion equivalent over $\mathcal {F}\uplus \{c\}$. Due Lemma 28 and symmetry, it suffices to show the inclusion ${\leftrightarrow _{\mathcal {R}}^{*\varepsilon *}} \subseteq {\leftrightarrow _{\mathcal {S}}^{*}}$ on terms in $\mathcal {T}(\mathcal {F},\mathcal {V})$. Suppose $s \leftrightarrow _{\mathcal {R}}^{*\varepsilon *} t$. Let $d \in \mathcal {F}$ be a constant, whose existence is guaranteed by the assumption $\mathcal {T}(\mathcal {F})\ne \varnothing$, and consider the substitutions $\sigma _c$ and $\sigma _d$ in the proof of Lemma 32. Closure under substitutions and variable separation yields $s\sigma _c \leftrightarrow _{\mathcal {R}}^{*\varepsilon *} t\sigma _c$ and $s\sigma _c \leftrightarrow _{\mathcal {R}}^{*\varepsilon *} t\sigma _d$. Ground conversion equivalence gives $s\sigma _c \leftrightarrow _{\mathcal {S}}^{*}t\sigma _c$ and $s\sigma _c \leftrightarrow _{\mathcal {S}}^{*}t\sigma _d$, and thus also $t\sigma _c \leftrightarrow _{\mathcal {S}}^{*}t\sigma _d$. Using Lemma 31 yields $s \leftrightarrow _{\mathcal {S}}^{*}t\sigma _d$ and $t \leftrightarrow _{\mathcal {S}}^{*}t\sigma _d$. Hence $s \leftrightarrow _{\mathcal {S}}^{*}t$ as desired. The only-if direction easily follows from Lemma 27. $\square$

Two fresh constants are required to reduce normalization equivalence to its ground version.

### Lemma 37

Linear variable-separated TRSs $\mathcal {R}$ and $\mathcal {S}$ over a common signature $\mathcal {F}$ are normalization equivalent if and only if $\mathcal {R}$ and $\mathcal {S}$ are ground normalization equivalent over $\mathcal {F}\uplus \{c,d\}$. 

### Proof

For the if direction we assume that $\mathcal {R}$ and $\mathcal {S}$ are ground normalization equivalent over $\mathcal {F}\uplus \{c,d\}$. Note that this implies that $\textsf{NF}_\mathcal {R}(t) \iff \textsf{NF}_\mathcal {S}(t)$ for all terms *t*. We apply Lemma 28 and symmetry, reducing the problem to $s \rightarrow _{\mathcal {R}}^{*\varepsilon *} t \implies \textsf{NF}_\mathcal {R}(t) \implies s \rightarrow _{\mathcal {S}}^{*} t$. Let $\sigma _c$ and $\sigma _d$ be substitutions replacing all variables by *c* and *d* respectively. Closure under substitution and variable separation yields $s\sigma _c \rightarrow _{\mathcal {R}}^{*\varepsilon *} t\sigma _d$, and $\textsf{NF}_\mathcal {R}(t\sigma _d)$ since *d* does not appear in $\mathcal {R}$. Ground normalization equivalence gives $s\sigma _c \rightarrow _{\mathcal {S}}^{*} t\sigma _d$. Applying Lemma 31 we obtain the desired $s \rightarrow _{\mathcal {S}}^{*} t$. The only-if direction follows from Lemma 27. $\square$

Contrary to Lemma 36 one fresh constant is not sufficient as seen by the following counterexample.

### Example 25

Consider the two linear variable-separated TRSs

$$\begin{aligned}&\mathcal {R}:&\textsf{a}&\rightarrow \textsf{b}&\textsf{f}(\textsf{f}(x,y),z)&\rightarrow \textsf{f}(\textsf{b},\textsf{b})&\textsf{f}(\textsf{b},x)&\rightarrow \textsf{f}(\textsf{b},\textsf{b}) \\{} & {} \textsf{f}(x,\textsf{a})&\rightarrow \textsf{f}(z,\textsf{b}) \\&\mathcal {S}:&\textsf{a}&\rightarrow \textsf{b}&\textsf{f}(\textsf{f}(x,y),z)&\rightarrow \textsf{f}(\textsf{b},\textsf{b})&\textsf{f}(\textsf{b},x)&\rightarrow \textsf{f}(\textsf{b},\textsf{b}) \\{} & {} \textsf{f}(\textsf{b},\textsf{a})&\rightarrow \textsf{f}(z,\textsf{b})&\textsf{f}(\textsf{f}(x,y),\textsf{a})&\rightarrow \textsf{f}(z,\textsf{b}) \end{aligned}$$

They are not normalization equivalent since $\textsf{f}(x,\textsf{a}) \rightarrow _{\mathcal {R}}^{!} \textsf{f}(z,\textsf{b})$ and $\textsf{f}(x,\textsf{a}) \not \rightarrow _{\mathcal {S}}^{*} \textsf{f}(z,\textsf{b})$. The TRSs are however ground normalization equivalent over the signature $\mathcal {F}\uplus \{\textsf{c}\}$. First observe that the only ground normal forms reachable via a rewrite sequence involving a root step are $\textsf{b}$ and $\textsf{f}(\textsf{c},\textsf{b})$. The normal form $\textsf{b}$ is reached (using a root step) only from $\textsf{a}$, in both $\mathcal {R}$ and $\mathcal {S}$. The normal form $\textsf{f}(\textsf{c},\textsf{b})$ can be reached from all ground terms of the shape $\textsf{f}(t,\textsf{a})$. For $\mathcal {R}$ this is obvious and for $\mathcal {S}$ this can be seen by a case analysis on the root symbol of *t*. Adding a second constant $\textsf{d}$ allows one to mimick the original counterexample since $\textsf{f}(\textsf{c},\textsf{a}) \rightarrow _{\mathcal {R}}^{!} \textsf{f}(\textsf{d},\textsf{b})$ and $\textsf{f}(\textsf{c},\textsf{a}) \not \rightarrow _{\mathcal {S}}^{*} \textsf{f}(\textsf{d},\textsf{b})$.

For left-linear right-ground TRSs, a single fresh constant is enough to reduce normalization equivalence to ground normalization equivalence.

### Lemma 38

Left-linear right-ground TRSs $\mathcal {R}$ and $\mathcal {S}$ over a common signature $\mathcal {F}$ are normalization equivalent if and only if $\mathcal {R}$ and $\mathcal {S}$ are ground normalization equivalent over $\mathcal {F}\uplus \{c\}$. 

### Proof

We mention the differences with the proof of Lemma 37. For the equivalence of $\textsf{NF}_\mathcal {R}(t)$ and $\textsf{NF}_\mathcal {S}(t)$ for arbitrary terms $t \in \mathcal {T}(\mathcal {F},\mathcal {V})$, a single constant suffices. If $s \rightarrow _{\mathcal {R}}^{*\varepsilon *} t$ then *t* is ground. Hence $s\sigma _c \rightarrow _{\mathcal {R}}^{*} t$ and thus $s\sigma _c \rightarrow _{\mathcal {S}}^{*} t$ by ground normalization equivalence. Lemma 31 gives $s \rightarrow _{\mathcal {S}}^{*} t$. $\square$

Each additional constant can increases the execution time of FORT-h significantly, as seen later in Example 36. Hence results that reduce the required number are of obvious interest. In the remainder of this section we present results for ground TRSs and for TRSs over *monadic* signatures, which are signatures that consist of constants and unary function symbols.

### Lemma 39

Let $\mathcal {R}$ and $\mathcal {S}$ be right-ground TRSs over a signature $\mathcal {F}$. If $\mathcal {R}$ and $\mathcal {S}$ are ground or $\mathcal {F}$ is monadic then 
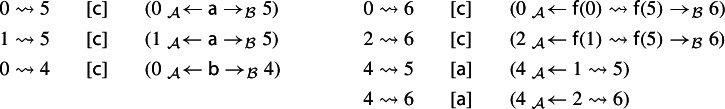


### Proof

First assume that $\mathcal {R}$ is ground. In this case only ground subterms can be rewritten. Given a term $t \in \mathcal {T}(\mathcal {F},\mathcal {V})$, we write $t = C[\![t_1,\dotsc ,t_{n}]\!]$ if $t = C[t_1,\dotsc ,t_{n}]$ and $t_1,\dotsc ,t_{n}$ are the maximal ground subterms of *t*. So all variables appearing in *t* occur in *C*. The following property is obvious:

- if $t = C[\![t_1,\dotsc ,t_{n}]\!] \rightarrow _{\mathcal {R}}^{*} u$ then $u = C[\![u_1,\dotsc ,u_{n}]\!]$ and $t_i \rightarrow _{\mathcal {R}}^{*} u_i$ for all $1 \leqslant i \leqslant n$.

Suppose $(\mathcal {F},\mathcal {R}) \vDash \textsf{GCR}$ and consider $s \rightarrow _{\mathcal {R}}^{*} t$ and $s \rightarrow _{\mathcal {R}}^{*} u$ with $s \in \mathcal {T}(\mathcal {F},\mathcal {V})$. Writing $s = C[\![s_1,\dotsc ,s_{n}]\!]$, we obtain $t = C[\![t_1,\dotsc ,t_{n}]\!]$ and $u = C[\![u_1,\dotsc ,u_{n}]\!]$ with $s_i \rightarrow _{\mathcal {R}}^{*} t_i$ and $s_i \rightarrow _{\mathcal {R}}^{*} u_i$ for all $1 \leqslant i \leqslant n$. $\textsf{GCR}$ yields $t_i \downarrow u_i$ for all $1 \leqslant i \leqslant n$. Hence $t \downarrow u$ as desired. The proofs for the other properties in $\mathcal {P}$ are equally easy. For $\textsf{UNC}$ we note that $\leftrightarrow _{\mathcal {R}}^{*}$ coincides with $\rightarrow _{\mathcal {R}\cup \mathcal {R}^-}^{*}$ for the *ground* TRS $\mathcal {R}\cup \mathcal {R}^-$.

Next suppose that $\mathcal {F}$ is monadic. Let $(\mathcal {F},\mathcal {R}) \vDash \textsf{G}P$ and let $\sigma$ be the substitution that maps all variables to some arbitrary but fixed ground term. In this case the following property holds:

(b) if $t \in \mathcal {T}(\mathcal {F},\mathcal {V})$ and $t \rightarrow u$ then $u \in \mathcal {T}(\mathcal {F})$ and $t\sigma \rightarrow u$.

We consider $P = \textsf{NFP}$ and $P = \textsf{UNC}$ and leave the proof for the other properties to the reader. Let $s \rightarrow _{\mathcal {R}}t$ and $s \rightarrow _{\mathcal {R}}^{!} u$. We obtain $s\sigma \rightarrow _{\mathcal {R}}t$ and $s\sigma \rightarrow _{\mathcal {R}}^{!} u$ from property 2. (Note that $s \ne u$.) Hence $t \rightarrow _{\mathcal {R}}^{*} u$ follows from $\textsf{GNFP}$. Let $t \leftrightarrow _{\mathcal {R}}^{*}u$ with normal forms *t* and *u*. If *t* and *u* are ground terms then we obtain $t = u$ from $\textsf{GUNC}$ (after applying the substitution $\sigma$ to all intermediate terms in the conversion between *t* and *u*). Otherwise, the conversion between *t* and *u* must be empty due to property (b) and the fact that *t* and *u* are normal forms. Hence also in this case $t = u$. $\square$

In contrast to $\textsf{COM}$, the properties $\textsf{NE}$ and $\textsf{CE}$ require additional constants for TRSs over monadic signatures.

### Example 26

The linear variable-separated TRSs

$$\begin{aligned} \mathcal {R}:\quad \textsf{f}(x)&\rightarrow \textsf{a}&\mathcal {S}:\quad \textsf{f}(\textsf{a})&\rightarrow \textsf{a} \qquad \textsf{f}(\textsf{f}(\textsf{a})) \rightarrow \textsf{a} \end{aligned}$$

are neither normalization equivalent nor conversion equivalent as can be seen from $\textsf{f}(x) \rightarrow _{\mathcal {R}}^{!} \textsf{a}$ and $\textsf{f}(x) \not \leftrightarrow _{\mathcal {S}}^{*}\textsf{a}$. Since every ground term rewrites in $\mathcal {R}$ and in $\mathcal {S}$ to the unique ground normal form $\textsf{a}$, the TRSs are ground normalization equivalent as well as ground conversion equivalent.

Nevertheless, we can reduce the number of constants to one if the signature is monadic. A key observation is that in non-empty rewrite sequences in a linear variable-separated TRS over a monadic signature fresh constants can be replaced by arbitrary terms.

### Lemma 40

Let $\mathcal {R}$ be a linear variable-separated TRS over a monadic signature $\mathcal {F}$ that contains a constant *c* which does not appear in $\mathcal {R}$. If $s \rightarrow _{\mathcal {R}}^{+} t$ and $p \in \mathcal {P}\textsf{os}(s)$ such that $s|_p = c$ then $s[u]_p \rightarrow _{\mathcal {R}}^{+} t$ using the same rewrite rules at the same positions, for all terms *u*. 

The proof follows the same structure as Lemma 31 and the details are left for the reader. As linear variable-separated TRSs are closed under inverse we can immediately deduce that rewrite sequences of the shape $s\sigma _c \rightarrow _{\mathcal {R}}^{+} t\sigma _c$ imply $s \rightarrow _{\mathcal {R}}^{+} t$ for monadic systems. With this we are ready to prove our claim.

### Lemma 41

Linear variable-separated TRSs $\mathcal {R}$ and $\mathcal {S}$ over a common monadic signature $\mathcal {F}$ are normalization equivalent if and only if $\mathcal {R}$ and $\mathcal {S}$ are ground normalization equivalent over $\mathcal {F}\uplus \{c\}$. 

### Proof

We again mention the differences with the proof of Lemma 37. For the equivalence of $\textsf{NF}_\mathcal {R}(t)$ and $\textsf{NF}_\mathcal {S}(t)$ for arbitrary terms $t \in \mathcal {T}(\mathcal {F},\mathcal {V})$, a single constant suffices. Consider a rewrite sequence $s \rightarrow _{\mathcal {R}}^{*\varepsilon *} t$ with $\textsf{NF}_\mathcal {R}(t)$. Ground normalization equivalence and substitution closure yields $s\sigma _c \rightarrow _{\mathcal {S}}^{*} t\sigma _c$. Furthermore, since the sequence $s \rightarrow _{\mathcal {R}}^{*\varepsilon *} t$ is non-empty by definition we know that $\lnot \textsf{NF}_{\mathcal {R}}(s\sigma _c)$, which in turn yields $\lnot \textsf{NF}_{\mathcal {S}}(s\sigma _c)$. Together with $\textsf{NF}_{\mathcal {S}}(t\sigma _c)$ this means $s\sigma _c \ne t\sigma _c$, and we obtain $s\sigma _c \rightarrow _{\mathcal {S}}^{+} t\sigma _c$. Applying Lemma 40 twice allows us to replace *c* in $s\sigma _c$ and $t\sigma _c$ by the corresponding variables, leading to $s \rightarrow _{\mathcal {S}}^{*} t$. $\square$

The following example shows that we cannot reduce the number of constants (in Lemmata 32 and 34) for linear variable-separated TRSs over a monadic signature and properties $P \in \mathcal {P}_1 \cup \{\textsf{COM}\}$.

### Example 27

The monadic linear variable-separated TRS $\mathcal {R}$ consisting of the rules

$$\begin{aligned} \textsf{g}(\textsf{a})&\rightarrow \textsf{g}(x)&\textsf{g}(\textsf{g}(x))&\rightarrow \textsf{g}(y) \end{aligned}$$

does not satisfy $\textsf{WCR}$ and $\textsf{UNR}$, and hence also not $\textsf{CR}$, $\textsf{SCR}$, $\textsf{NFP}$ and $\textsf{UNC}$, because $\textsf{g}(x) \leftarrow \textsf{g}(\textsf{a}) \rightarrow \textsf{g}(y)$ with different normal forms $\textsf{g}(x)$ and $\textsf{g}(y)$. Adding a single fresh constant $\textsf{c}$ is insufficient to violate $\textsf{GSCR}$ and thus also $\textsf{GCR}$, $\textsf{GWCR}$, $\textsf{GNFP}$, $\textsf{GUNC}$ and $\textsf{GUNR}$, because every term in $\mathcal {T}(\{\textsf{g}, \textsf{a}, \textsf{c}\})$ can reach precisely one of the three ground normal forms $\textsf{a}$, $\textsf{c}$ or $\mathsf {g(c)}$ and they can all do so in at most one step. Adding an additional constant $\textsf{d}$ does suffice: $\textsf{g}(\textsf{c}) \leftarrow \textsf{g}(\textsf{a}) \rightarrow \textsf{g}(\textsf{d})$ with different ground normal forms $\textsf{g}(\textsf{c})$ and $\textsf{g}(\textsf{d})$. The same behaviour is observed for $\textsf{COM}$ by noting that a TRS is (ground) confluent if and only if it (ground) commutes with itself.

The results in this section are summarized in Table , which shows the number of additional constants needed to reduce a property to the corresponding property on ground terms. In parentheses are the numbers for monadic TRSs.

**Table 3:** Additional constants required to reduce a property to the corresponding ground property

Property	Ground TRSs	Left-linear right-ground TRSs	Linear variable-separated TRSs
$\textsf{CR}$	0	1 (0)	2 (2)
$\textsf{SCR}$	0	1 (0)	2 (2)
$\textsf{WCR}$	0	1 (0)	2 (2)
$\textsf{COM}$	0	1 (0)	2 (2)
$\textsf{UNR}$	0	1 (0)	2 (2)
$\textsf{UNC}$	0	2 (0)	2 (2)
$\textsf{NFP}$	0	1 (0)	2 (2)
$\textsf{CE}$	0	1 (1)	1 (1)
$\textsf{NE}$	0	1 (1)	2 (1)

For termination ($\textsf{SN}$) and normalization ($\textsf{WN}$) there is no need to add fresh constants, since these properties hold if and only if they hold for all ground terms. For other properties that can be expressed in the first-order theory of rewriting, one or two fresh constants may be insufficient. Consider for instance the formula $\varphi$:

$$\begin{aligned} \lnot \,\exists \,s\,\exists \,t\,\exists \,u\,\forall \,v\, (v \leftrightarrow _{}^{*}s \,\vee \, v \leftrightarrow _{}^{*}t \,\vee \, v \leftrightarrow _{}^{*}u) \end{aligned}$$

which is satisfied on arbitrary terms (with respect to any left-linear right-ground TRS $(\mathcal {F},\mathcal {R})$). For the TRS consisting of the rule $\textsf{f}(x) \rightarrow \textsf{a}$ and two additional constants $\textsf{c}$ and $\textsf{d}$, $\varphi$ does not hold for ground terms because every ground term is convertible to $\textsf{a}$, $\textsf{c}$ or $\textsf{d}$. It is tempting to believe that adding a fresh unary symbol *g* in addition to a fresh constant *c*, in order to create infinitely many ground normal forms which can replace variables that appear in open terms, is sufficient for any property *P*. The formula $\forall \,s\,\forall \,t\,(s \rightarrow t \implies s \rightarrow _{\varepsilon } t)$ and the TRS consisting of the rule $\textsf{a} \rightarrow \textsf{b}$ show that this is incorrect.

## Automation and Certification

**Fig. 7:**
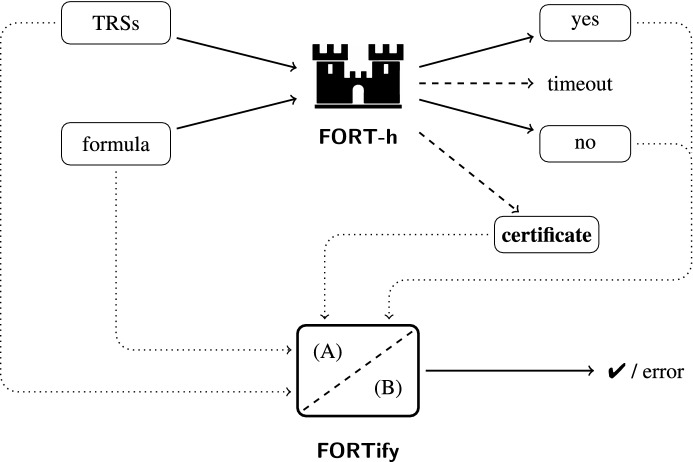
FORT-h and FORTify

### Decision Mode

FORT-h is a new decision tool for the first order theory of rewriting. It is a reimplementation of the decision mode of the previous FORT tool [48], referred to as FORT-j in the remainder of the paper. The decision procedure implemented in FORT-j is based on the original procedure described in [10, 11], in which the basic relations are one-step and parallel rewriting. Anchored GTTs, which form the backbone of the formalized decision procedure described in this paper and implemented in FORT-h, were developed later. The new tool is implemented in Haskell whereas FORT-j is written in Java. FORT-h supports all features of FORT-j while extending the domain of supported TRSs from left-linear right-ground TRSs to *linear variable-separated* ones. While FORT-j could technically take such TRSs as input, it is unsound when checking non-ground properties on them.

#### Example 28

To check confluence of the linear variable-separated TRS

$$\begin{aligned} \textsf{g}(\textsf{g}(x))&\rightarrow \textsf{g}(y)&\textsf{a}&\rightarrow \textsf{g}(\textsf{a}) \end{aligned}$$

FORT-h can be called with the formula CR. It correctly states that NO the system is not confluent. However, FORT-j incorrectly identifies this as confluent due to the lack of support for variables appearing in right-hand sides of rules.

FORT-h took part in the 2020, 2021 and 2022 editions of the Confluence Competition (CoCo),^4^ competing in five categories: $\textsf{COM}$, $\textsf{GCR}$, $\textsf{NFP}$, $\textsf{UNC}$ and $\textsf{UNR}$. In 2021 and 2022 it also competed together with FORTify in the categories $\textsf{COM}$, $\textsf{TRS}$, $\textsf{GCR}$, $\textsf{UNC}$, $\textsf{UNR}$ and $\textsf{NFP}$ (only in 2022) producing certified answers. Even though it does not support many problems tested in the competition, due to the restriction to linear variable-separated TRSs, it was able to win the category for most YES results in UNR in all three years. The tool expects as input a formula and one or more TRSs, as seen in Fig. . It then outputs the answer YES or NO depending on whether the formula is satisfied or not by the given TRSs. The command-line interface of FORT-h is described in Appendix B.

The implemented procedure closely follows the procedure described in Sect. 5.5. When called it first parses the formula (format described below) and converts it into an internal represention using de Bruijn indices as described in Sect. 7.2. Additionally, universal quantifiers and implications are eliminated, and negations are pushed as far as possible to the atomic subformulas. The tool then traverses the formula in a bottom-up fashion, constructing the corresponding anchored GTTs and $\textsf{RR}_n$ automata. During this traversal we also keep track of the steps taken, to construct the certificate if necessary. To improve performance the automata are cached and reused for equal subformulas. The tree automaton representing the whole formula is then checked for emptiness. If the accepted language is empty, FORT-h reports NO, otherwise it outputs YES.

To avoid having to write formulas using de Bruijn indices when using FORT-h, we use a more convenient syntax for interacting with the tool. The input format (later called FORT syntax) is described in Appendix A.

#### Witness Generation

The usual output of FORT-h consists of a YES or NO answer, and possibly a certificate containing size information of the automata. To help the user in understanding why a property holds or does not hold we support witness generation. This is possible in two cases. Firstly for *satisfiable existentially quantified* formulas, where FORT-h can produce an *n*-tuple of ground terms as evidence of existence. Secondly for *unsatisfiable universally quantified* formulas, where the tuple presents a counterexample. For instance, if a given or synthesized TRS is not ground-confluent $\lnot \,\forall \,s\,\forall \,t\,\forall \,u\,(s \rightarrow _{}^{*} t \,\wedge \, s \rightarrow _{}^{*} u \,\implies \, \exists \,v\,(t \rightarrow _{}^{*} v \,\wedge \, u \rightarrow _{}^{*} v))$, it is interesting to provide witnessing terms for the variables *s*, *t*, and *u*. Given the TRS consisting of the rules

$$\begin{aligned} \textsf{a}&\rightarrow \textsf{f}(\textsf{a},\textsf{b})&\textsf{f}(\textsf{a},\textsf{b})&\rightarrow \textsf{f}(\textsf{b},\textsf{a}) \end{aligned}$$

FORT-h produces the following terms as witnesses: $s = \textsf{f}(\textsf{a},\textsf{b})$, $t = \textsf{f}(\textsf{b},\textsf{a})$, and $u = \textsf{f}(\textsf{f}(\textsf{a},\textsf{b}),\textsf{b})$. To find these ground terms FORT-h first eliminates universal quantifiers using $\forall = \lnot \,\exists \,\lnot$, pushes negations inwards and removes double negations in the formula resulting in $\exists \,s\,\exists \,t\,\exists \,u\, (s \rightarrow _{}^{*} t \,\wedge \, s \rightarrow _{}^{*} u \,\wedge \, \lnot \,\exists \,v\,(t \rightarrow _{}^{*} v \,\wedge \, u \rightarrow _{}^{*} v))$. In the next step FORT-h strips outermost negations, none in this case, followed by outermost existential quantifiers resulting in the so-called *formula body*: $(s \rightarrow _{}^{*} t \,\wedge \,s \rightarrow _{}^{*} u \,\wedge \, \lnot \,\exists \,v\,(t \rightarrow _{}^{*} v \,\wedge \, u \rightarrow _{}^{*} v))$. Since the original formula is satisfiable, the $\textsf{RR}_{n}$ automaton corresponding to the formula body must accept at least one *n*-tuple of ground terms.

**Fig. 8:**
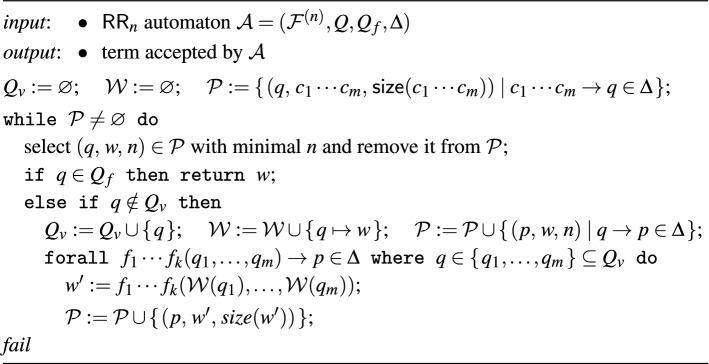
Witness generation

The algorithm depicted in Fig.   generates (encoded) witnesses that are accepted by the given $\textsf{RR}_{n}$ automaton. To find minimal witnesses we use a version of Dijkstra’s shortest path algorithm. We keep track of visited states in $Q_v$, a mapping $\mathcal {W}$ from states to terms where $\mathcal {W}(q)$ is a minimal witness which reaches the state *q*, and a priority queue $\mathcal {P}$. The search is started at the states reachable in a single step from some constant. We also map from these states to the respective constants as witnesses in $\mathcal {W}$. In each iteration we select the state *q* with the smallest witness *w* from $\mathcal {P}$. The size of a witness is determined by the function $\textsf {size}(\langle w_1,\dotsc ,w_{n} \rangle ) = \textsf {size}(w_1) + \cdots + \textsf {size}(w_n)$, where $\textsf {size}(w_i)$ is the total number of function symbols in $\mathcal {F}$ occurring in $w_i$, so $\bot$ is not counted. If *q* is a final state we have found an accepted term and return the witness *w*. Otherwise we check that we have not visited *q* previously, set $\mathcal {W}(q) = w$, and enumerate all transition rules containing *q* on the left-hand side where all states on the left-hand side have been visited, and hence have a witness. If the transition rule is an epsilon transition $q \rightarrow p$, then the state *p* has the same witness as *q* so we add $(p,\,w,\,\textsf {size}(w))$ to $\mathcal {P}$. For a transition rule $f_1 \cdots f_k(q_1,\dotsc ,q_{m}) \rightarrow p$ we construct a witness $w' = f_1 \cdots f_k(\mathcal {W}(q_1),\dotsc ,\mathcal {W}(q_m))$ and add $(p,w,\textsf {size}(w))$ to the queue. The search continues until a final state is reached or all reachable states have been visited. In the latter case the algorithm fails, since the automaton does not accept any terms.

#### Collapsing ${\varvec{\varepsilon }}$-transitions

Keeping the size of automata small is crucial for the performance of FORT-h. One way to reduce the number of states and transitions is based on the observation that when two states *q* an *p* are strongly connected by $\varepsilon$-transitions, which means $q \rightarrow _{\varepsilon }^{*} p$ and $p \rightarrow _{\varepsilon }^{*} q$, then they are equivalent. In other words, for all ground terms *s* and *t* we have $s \rightarrow _{}^{*} q$ if and only if $t \rightarrow _{}^{*} p$, and for all ground contexts *C* and states *r* we have $C[q] \rightarrow _{}^{*} r$ if and only if $C[p] \rightarrow _{}^{*} r$. We can therefore replace all occurrences of a state in the transition rules by an equivalent one without changing the accepted language. This reduces the number of states, and may remove duplicate transition rules.

In FORT-h we can further take advantage of the fact that some of the most common constructions already produce sets of $\varepsilon$-transitions which are transitively closed. Instead of constructing the strongly connected components, checking if two states *q* and *p* are strongly connected then boils down to checking if $q \rightarrow _{\varepsilon } p$ and $p \rightarrow _{\varepsilon } q$. For example, this is case after computing the transitive closure of anchored GTT relations as in the Theorems 6 and 8. We therefore immediately collapse and eliminate the $\varepsilon$-transitions in the underlying tree automata after these constructions.

##### Example 29

**Fig. 9:**
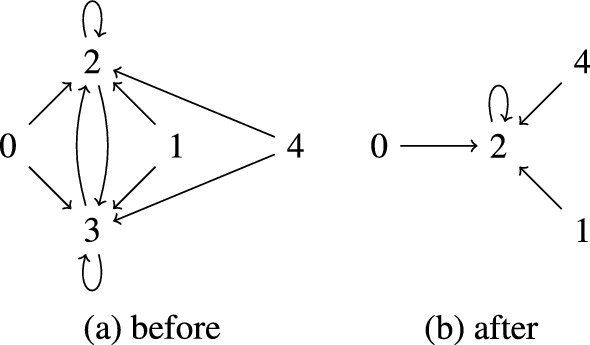
Collapsing $\varepsilon$-transitions in $\Delta _+(\mathcal {A},\mathcal {B})$

The anchored GTT $G = (\mathcal {A},\mathcal {B})$ with

$$\begin{aligned} \Delta _{\mathcal {A}}:{} & {} \textsf{a}&\rightarrow 0&\textsf{b}&\rightarrow 1 \\{} & {} 0&\rightarrow 3&1&\rightarrow 2&1&\rightarrow 4 \\ \Delta _{\mathcal {B}}:{} & {} \textsf{a}&\rightarrow 2&\textsf{b}&\rightarrow 3&\textsf{c}&\rightarrow 4 \end{aligned}$$

accepts the rewrite relation of the ARS $\{\textsf{a} \rightarrow \textsf{b}, \textsf{b} \rightarrow \textsf{a}, \textsf{b} \rightarrow \textsf{c}\}$. When constructing $\mathcal {G}_+ = (\mathcal {A}\cup \Delta _+(\mathcal {B},\mathcal {A}),\mathcal {B}\cup \Delta _+(\mathcal {A},\mathcal {B}))$, we need to compute the $\varepsilon$-transitions in $\Delta _+(\mathcal {A},\mathcal {B})$. The result is shown in Fig. (a). We can see that the graph contains one non-trivial strongly connected component, consisting of the states $\{2,3\}$. Instead of adding all 10 $\varepsilon$-transitions we can therefore simplify *G* and $\Delta _+$ beforehand by replacing all occurrences of state 3 by state 2. This reduces the number of transitions in $\Delta _+(\mathcal {A},\mathcal {B})$ to 4, as shown in Fig. 9(b), which, when added to *G*, results in the GTT $G_+ = (\mathcal {A}',\mathcal {B}')$ with

$$\begin{aligned} \Delta _{\mathcal {A}'}:{} & {} \textsf{a}&\rightarrow 0&\textsf{b}&\rightarrow 1 \\{} & {} 0&\rightarrow 2&1&\rightarrow 2&1&\rightarrow 4&2&\rightarrow 0&2&\rightarrow 4&2&\rightarrow 1 \\ \Delta _{\mathcal {B}'}:{} & {} \textsf{a}&\rightarrow 2&\textsf{b}&\rightarrow 2&\textsf{c}&\rightarrow 4 \\{} & {} 0&\rightarrow 2&4&\rightarrow 2&1&\rightarrow 2 \end{aligned}$$

Note that we also dropped the redundant transition $2 \rightarrow 2$ from $\Delta _+(\mathcal {A},\mathcal {B})$.

### Certification

Whereas witness generation can only provide some evidence to assist the user in understanding why certain formulas hold or not, in certification we are interested in machine-readable proofs that are verified by an independent and trustworthy certifier. The first step in the certification process is to translate formulas in the first-order theory of rewriting into a format suitable for further processing. We adopt de Bruijn indices [13] to avoid alpha renaming.

#### Example 30

Consider the formula

$$\begin{aligned} \forall \,s\,\forall \,t\,\forall \,u\,(s \rightarrow _{0}^{*} t \wedge s \rightarrow _{1}^{*} u \implies \exists \,v\,(t \rightarrow _{1}^{*} v \wedge u \rightarrow _{0}^{*} v)) \end{aligned}$$

It expresses the *commutation* of two TRSs, indicated by the indices 0 and 1. Using de Bruijn indices for the term variables *s*, *t*, *u*, *v* produces

$$\begin{aligned} \forall \,\forall \,\forall \,(2 \rightarrow _{0}^{*} 1 \wedge 2 \rightarrow _{1}^{*} 0) \implies \exists \,(2 \rightarrow _{1}^{*} 0 \wedge 1 \rightarrow _{0}^{*} 0) \end{aligned}$$

We refer to Example 32 for further explanation.

The formal syntax of formulas in certificates is given below. Here $\langle \textit{rr}_2\rangle$ denotes the supported binary regular relations, which are formally defined after Example 31. Likewise, $\langle \textit{rr}_1\rangle$ stands for regular sets (which are identified with unary regular relations).

$$\begin{aligned} \langle \textit{formula}\rangle ~::=~&\texttt {(rr1 }\,\langle \textit{rr}_1\rangle \,\langle \textit{term}\rangle \texttt {) } ~|~ \texttt {(rr2 }\,\langle \textit{rr}_2\rangle \,\langle \textit{term}\rangle \,\langle \textit{term}\rangle \texttt {) } \\ ~|~&\texttt {(and }\,\langle \textit{formula}\rangle * \texttt {) } ~|~ \texttt {(or }\,\langle \textit{formula}\rangle * \texttt {) } ~|~ \texttt {(not }\,\langle \textit{formula}\rangle \texttt {) } \\ ~|~&\texttt {(forall }\,\langle \textit{formula}\rangle \texttt {) } ~|~ \texttt {(exists }\,\langle \textit{formula}\rangle \texttt {) } ~|~ \texttt {(true) } ~|~ \texttt {(false) } \\ ~|~&\texttt {(restrict }\,\langle \textit{formula}\rangle \,\texttt {( }\,\langle \textit{trs}\rangle + \texttt {)) } \\ \langle \textit{term}\rangle ~::=~&\langle \textit{nat}\rangle \qquad \langle \textit{trs}\rangle ~::=~ \langle \textit{nat}\rangle ~|~ \langle \textit{nat}\rangle \,\texttt {- } \qquad \langle \textit{nat}\rangle ~::=~ \texttt {0 } ~|~ \texttt {1 } ~|~ \texttt {2 } ~|~ \cdots \end{aligned}$$

De Bruijn indices are used for $\langle \textit{term}\rangle$ variables and $\langle \textit{nat}\rangle \,\texttt {- }$ denotes a TRS with index $\langle \textit{nat}\rangle$ in which the left- and right-hand sides of the rules have been swapped. The class of linear variable-separated TRSs is closed under this operation. We use it to represent the conversion relation $\leftrightarrow _{}^{*}$ of a TRS $\mathcal {R}$ as the reachability relation $\rightarrow ^{*}$ induced by the TRS $\mathcal {R}\cup \mathcal {R}^-$.

#### Example 31

The commutation property in Example 30 is rendered as follows:

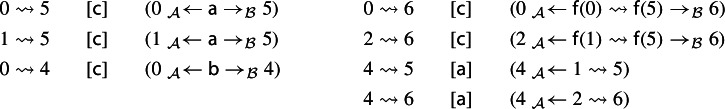

Here (step* (0)) denotes the $\textsf{RR}_2$ relation $\rightarrow ^{*}$ induced by the first TRS (which is indexed by 0) and (rr2 (step* (1)) 2 0) represents the subformula [1] t ->* v of the FORT formula in Example 30.

We continue with the certificate syntax of $\textsf{RR}_1$ and $\textsf{RR}_2$ relations:

$$\begin{aligned} \langle \textit{rr}_1\rangle ~::=~&\texttt {(terms) } ~|~ \texttt {(nf }\,\texttt {( }\,\langle \textit{trs}\rangle + \texttt {)) } ~|~ \texttt {(inf }\,\langle \textit{rr}_2\rangle \texttt {) } ~|~ \texttt {(proj }\,(\texttt {1 } \,|\, \texttt {2 })\,\langle \textit{rr}_2\rangle \texttt {) } \\ ~|~&\texttt {(union }\,\langle \textit{rr}_1\rangle \,\langle \textit{rr}_1\rangle \texttt {) } ~|~ \texttt {(inter }\,\langle \textit{rr}_1\rangle \,\langle \textit{rr}_1\rangle \texttt {) } ~|~ \texttt {(diff }\,\langle \textit{rr}_1\rangle \,\langle \textit{rr}_1\rangle \texttt {) } \\ \langle \textit{rr}_2\rangle ~::=~&\texttt {(gtt }\,\langle \textit{gtt}\rangle \,\langle \textit{pos}\rangle \,\langle \textit{num}\rangle \texttt {) } ~|~ \texttt {(product }\,\langle \textit{rr}_1\rangle \,\langle \textit{rr}_1\rangle \texttt {) } ~|~ \texttt {(id }\,\langle \textit{rr}_1\rangle \texttt {) } \\ ~|~&\texttt {(union }\,\langle \textit{rr}_2\rangle \,\langle \textit{rr}_2\rangle \texttt {) } ~|~ \texttt {(inter }\,\langle \textit{rr}_2\rangle \,\langle \textit{rr}_2\rangle \texttt {) } ~|~ \texttt {(diff }\,\langle \textit{rr}_2\rangle \,\langle \textit{rr}_2\rangle \texttt {) } \\ ~|~&\texttt {(comp }\,\langle \textit{rr}_2\rangle \,\langle \textit{rr}_2\rangle \texttt {) } ~|~ \texttt {(inverse }\,\langle \textit{rr}_2\rangle \texttt {) } \\ \langle pos \rangle ~::=~&\texttt {>= } ~|~ \texttt {e } ~|~ \texttt {> } \qquad \langle \textit{num}\rangle ~::=~ \texttt {>= } ~|~ \texttt {1 } ~|~ \texttt {> } \\ \langle gtt \rangle ~::=~&\texttt {(root-step }\,\texttt {( }\,\langle \textit{trs}\rangle + \texttt {)) } ~|~ \texttt {(gsteps }\,\texttt {( }\,\langle \textit{trs}\rangle + \texttt {)) } ~|~ \texttt {(inverse }\,\langle \textit{gtt}\rangle \texttt {) } \\ ~|~&\texttt {(union }\,\langle \textit{gtt}\rangle \,\langle \textit{gtt}\rangle \texttt {) } ~|~ \texttt {(acomp }\,\langle \textit{gtt}\rangle \,\langle \textit{gtt}\rangle \texttt {) } ~|~ \texttt {(gcomp }\,\langle \textit{gtt}\rangle \,\langle \textit{gtt}\rangle \texttt {) } \\ ~|~&\texttt {(inter }\,\langle \textit{gtt}\rangle \,\langle \textit{gtt}\rangle \texttt {) } ~|~ \texttt {(acomplement }\,\langle \textit{gtt}\rangle \texttt {) } ~|~ \texttt {(atc }\,\langle \textit{gtt}\rangle \texttt {) } ~|~ \texttt {(gtc }\,\langle \textit{gtt}\rangle \texttt {) } \end{aligned}$$

Here (terms) refers to $\mathcal {T}(\mathcal {F})$, $\texttt {(nf }\,\texttt {( }\,\langle \textit{trs}\rangle + \texttt {)) }$ to the normal forms ($\textsf{NF}$) induced by the union of the underlying TRSs, and $\texttt {(inf }\,\langle \textit{rr}_2\rangle \texttt {) }$ to the infinity predicate ($\textsf{INF}_R$) which is satisfied by all terms having infinitely many successors with respect to the relation *R*. Furthermore, $\texttt {(proj }\,(\texttt {1 } \,|\, \texttt {2 })\,\langle \textit{rr}_2\rangle \texttt {) }$ denotes projection ($\pi$) to the first (second) argument, $\texttt {(gtt }\,\langle \textit{gtt}\rangle \,\langle \textit{pos}\rangle \,\langle \textit{num}\rangle \texttt {) }$ the transformation of a GTT relation into an $\textsf{RR}_2$ relation with corresponding context closure (Theorems 10 and 11), $\texttt {(id }\,\langle \textit{rr}_1\rangle \texttt {) }$ the identity relation on the underlying set, and $\texttt {(gtc }\,\langle \textit{gtt}\rangle \texttt {) }$ ($\texttt {(atc }\,\langle \textit{gtt}\rangle \texttt {) }$) the (anchored) transitive closure of the underlying (anchored) GTT relation. The $\texttt {(gsteps }\,\texttt {( }\,\langle \textit{trs}\rangle + \texttt {)) }$ construct serves as an abbreviation for $\texttt {(gtc }~\texttt {((root-step }\,\texttt {( }\,\langle \textit{trs}\rangle + \texttt {)) } \texttt {)) }$.

The constructs defined above closely correspond to the formalized closure operations for the predicates in the first-order theory of rewriting, summarized in the grammar in Fig. 1.

For convenience of tool authors, we add a few other constructs to $\langle \textit{rr}_2\rangle$. The certifier expands these to a sequence of basic constructs given above.

$$\begin{aligned} \langle \textit{rr}_2\rangle ~::=~&\cdots ~|~ \texttt {(step }\,\texttt {( }\,\langle \textit{trs}\rangle + \texttt {)) } ~|~ \texttt {(step= }\,\texttt {( }\,\langle \textit{trs}\rangle + \texttt {)) } ~|~ \texttt {(step+ }\,\texttt {( }\,\langle \textit{trs}\rangle + \texttt {)) } \\ ~|~&\texttt {(step* }\,\texttt {( }\,\langle \textit{trs}\rangle + \texttt {)) } ~|~ \texttt {(step! }\,\texttt {( }\,\langle \textit{trs}\rangle + \texttt {)) } ~|~ \texttt {(equality) } \\ ~|~&\texttt {(parallel-step }\,\texttt {( }\,\langle \textit{trs}\rangle + \texttt {)) } ~|~ \texttt {(root-step }\,\texttt {( }\,\langle \textit{trs}\rangle + \texttt {)) } \\ ~|~&\texttt {(root-step= }\,\texttt {( }\,\langle \textit{trs}\rangle + \texttt {)) } ~|~ \texttt {(root-step+ }\,\texttt {( }\,\langle \textit{trs}\rangle + \texttt {)) } \\ ~|~&\texttt {(root-step* }\,\texttt {( }\,\langle \textit{trs}\rangle + \texttt {)) } ~|~ \texttt {(non-root-step }\,\texttt {( }\,\langle \textit{trs}\rangle + \texttt {)) } \\ ~|~&\texttt {(non-root-step= }\,\texttt {( }\,\langle \textit{trs}\rangle + \texttt {)) } ~|~ \texttt {(non-root-step+ }\,\texttt {( }\,\langle \textit{trs}\rangle + \texttt {)) } \\ ~|~&\texttt {(non-root-step* }\,\texttt {( }\,\langle \textit{trs}\rangle + \texttt {)) } ~|~ \texttt {(meet }\,\texttt {( }\,\langle \textit{trs}\rangle + \texttt {)) } \\ ~|~&\texttt {(join }\,\texttt {( }\,\langle \textit{trs}\rangle + \texttt {)) } ~|~ \texttt {(reflcl }\,\texttt {( }\,\langle \textit{rr}_2\rangle \texttt {)) } \end{aligned}$$

A certificate for a first-order formula $\varphi$ explains how the corresponding $\textsf{RR}_n$ automaton is constructed. We adopt a line-oriented natural deduction style. The automata are implicit. This is a deliberate design decision to keep certificates small. More importantly, it avoids having to check equivalence of finite tree automata, which is EXPTIME-complete [8, Sect. 1.7].

$$\begin{aligned} \langle \textit{certificate}\rangle ~::=~&\texttt {( }\,\langle \textit{item}\rangle \,\langle \textit{inference}\rangle \,\langle \textit{formula}\rangle \,\langle \textit{info}\rangle * \texttt {) } \, \langle \textit{certificate}\rangle \\ ~|~&\texttt {(empty }\,\langle \textit{item}\rangle \texttt {) } ~|~ \texttt {(nonempty }\,\langle \textit{item}\rangle \texttt {) } \\ \langle \textit{item}\rangle ~::=~&\langle \textit{nat}\rangle \qquad \langle \textit{info}\rangle ~::=~ \texttt {(size }\,\langle \textit{nat}\rangle \,\langle \textit{nat}\rangle \,\langle \textit{nat}\rangle \texttt {) } \\ \langle \textit{inference}\rangle ~::=~&\texttt {(rr1 }\,\langle \textit{rr}_1\rangle \,\langle \textit{term}\rangle \texttt {) } ~|~ \texttt {(rr2 }\,\langle \textit{rr}_2\rangle \,\langle \textit{term}\rangle \,\langle \textit{term}\rangle \texttt {) } ~|~ \texttt {(and }\,\langle \textit{item}\rangle * \texttt {) } \\ ~|~&\texttt {(or }\,\langle \textit{item}\rangle * \texttt {) } ~|~ \texttt {(not }\,\langle \textit{item}\rangle \texttt {) } ~|~ \texttt {(exists }\,\langle \textit{item}\rangle \texttt {) } ~|~ \texttt {(nnf }\,\langle \textit{item}\rangle \texttt {) } \end{aligned}$$

Currently the $\langle \textit{info}\rangle$ field only serves as an interface between the tool (which provides the certificate) and the certifier to compare the sizes of the constructed automata. In the future we plan to extend this field with concrete automata. This allows to test language equivalence of a tree automaton computed by a tool that supports our certificate language and the one reconstructed by FORTify, thereby providing tool authors with a mechanism to trace buggy constructions in case a certificate is rejected.

We revisit Example 3 to illustrate the construction of certificates.

#### Example 32

The formula $\varphi = \forall \,s\,\exists \,t\,(s \rightarrow ^{*} t\,\wedge \,\textsf{NF}(t))$ expressing normalization is rendered as $\varphi ' = \forall \exists (1 \rightarrow _{0}^{*} 0\,\wedge \,0 \in \textsf{NF}[0])$ in de Bruijn notation. Here 1 refers to the variable *s*, the second and third occurrences of 0 refer to *t*, and the last occurrence of 0 refer to the first (and only) TRS, which has index 0. We construct the certificate bottom-up, to mimic the decision procedure. The first line is for $\textsf{NF}[0]$: 
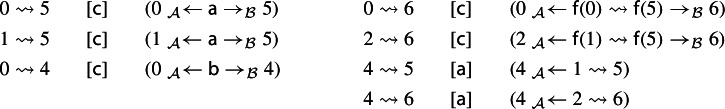
 The components can be read as follows:

- $\langle \textit{item}\rangle = \texttt {0}$ denotes the first step in our proof,
- $\langle \textit{inference}\rangle = \texttt {rr1 (nf (0)) 0}$ constructs the automaton that accepts the normal forms and keeps track of the variable 0,
- $\langle \textit{formula}\rangle = \texttt {rr1 (nf (0)) 0}$ denotes the subformula $0 \in \textsf{NF}[0]$; it is satisfiable if and only if the automaton constructed using the description in $\langle \textit{inference}\rangle$ is not empty.

The apparent redundancy will disappear when we continue. We proceed by expressing the relation $\rightarrow _{0}^{*}$ and subsequently make sure that the second component of $\rightarrow _{0}^{*}$ is in normal form: 
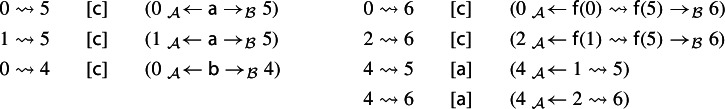
 Line 1 is similar to line 0. The inference step $\texttt {(and 1 0)}$ in line 2 constructs an $\textsf{RR}_2$ automaton that accepts the intersection of the relations modeled in lines 1 and 0. This automaton corresponds to $\mathcal {A}_5$ in Example 3. The cylindrification step from $\mathcal {A}_1$ to $\mathcal {A}_4$ in Example 3 is left implicit. We continue with the projection of variable 0 and afterwards complement the resulting automaton. This is done by an exists followed by a not inference step: 
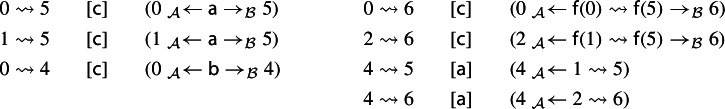
 The inference steps until this point describe the construction of $\mathcal {A}_7$ in Example 3. We complete the certificate by introducing the remaining operators: 
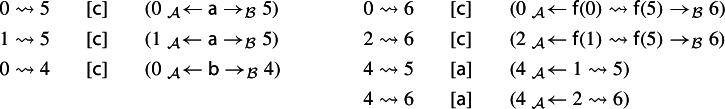
 The $\texttt {nnf }$ inference step does not modify the tree automaton computed in step 6 (which corresponds to $\mathcal {A}_9$ in Example 3) but checks the equivalence of the formula in line 6 with the one of line 7, which corresponds to the input formula $\varphi '$. The equivalence check incorporates $\forall$ elimination, negation normal form, and associativity, commutativity and idempotency of $\wedge$ and $\vee$. In the future we might add support for additional equivalences in first-order logic. The final step (nonempty 7) checks that $L(\mathcal {A}_9) \ne \varnothing$. So this certificate claims that the input TRS is normalizing. For TRSs that do not satisfy $\varphi$, the final line in the certificate would be (empty 7).

In the previous example we intentionally skipped over some details to convey the underlying intuition. First of all, the $\langle \textit{rr}_2\rangle$ construct $\texttt {(step* (0)) }$ is derived and internally unfolded via (anchored) GTTs into

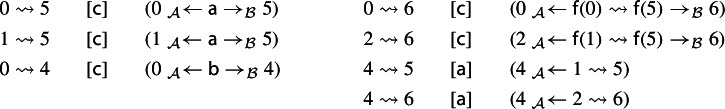

Starting from an anchored GTT that accepts the root step relation induced by the first (and only) TRS in the list, an application of the GTT transitive closure operation followed by a multi-hole context closure operation with at least one hole that may appear in any position, an $\textsf{RR}_{2}$ automaton that accepts the relation $\rightarrow _{0}^{*}$ is constructed. We also mentioned that cylindrification is implicit. The same holds for the projection operation that is used in the exists inference steps. A projection takes place in the first component if the variable 0 is present in the list of variables, otherwise the inference step preserves the automaton. This approach is sound as variables indicate the relevant components of the $\textsf{RR}_n$ automaton. Thanks to the de Bruijn representation, the innermost quantifier refers to variable 0, the first component in the given $\textsf{RR}_{n}$ automaton. However we must keep track of all variables occurring in the surrounding formula and update that list accordingly.

### FORTify

The example in the preceding subsection makes clear that certificate can be viewed as a recipe for the certifier to perform certain operations on automata and formulas to confirm the final (non-)emptiness claim. In particular, checking a certificate is expensive because the decision procedure for the first-order theory is replayed using code-generated operations from a verified version of the decision procedure. In this subsection we describe the steps we performed to turn the Isabelle formalization of the decision procedure into our certifier FORTify.

The formalization is split into two parts. The second part is about the certification process, but we start our description with the first part [35] which serves as a general tree automata library. This part includes bottom-up tree automata with $\varepsilon$-transitions, (anchored) ground tree transducers, encoding of regular relations, and their respective closure properties. Additionally it contains a framework to simplify code generation of inductively defined sets as in Fig. 3. Such inductive sets, if they are finite, can be computed by a saturation procedure. We provide an abstraction for that, which essentially does Horn inference without negative atoms. The point of the abstraction is that it separates a common iterative or recursive part of saturation procedures (which gives rise to non-trivial correctness proofs) from the enumeration of inferences without premises ($\mathcal {H}_0$, see below), and inferences induced by a single new conclusion ($\mathcal {H}_1$, also below), which usually are set comprehensions that can be computed in a very straightforward way.

#### Definition 19

A positive Horn inference system is given by a set of atoms *A* (with elements $\alpha$, $\beta$, ...) and set $\mathcal {H}$ of inference rules of the shape $\alpha _1 \wedge \dots \wedge \alpha _n \rightarrow \beta$. We write $\top \rightarrow \beta$ if the list of premises is empty. Each positive Horn inference system defines a predicate $\overline{\mathcal {H}}$ on atoms inductively by the rule

#### Example 33

Consider the inference rules from Fig. 3. To obtain a positive Horn inference system for given automata $\mathcal {A}$ and $\mathcal {B}$, let $A = Q \times Q$ where *Q* is the set of states occurring in $\mathcal {A}$ or $\mathcal {B}$. The set $\mathcal {H}$ consists of the following inference rules:

- $$(p,r) \rightarrow (q,r)$$ if $$p \rightarrow _{\!\mathcal {A}}q$$ and $$r \in Q$$,
- $(p,q) \rightarrow (p,r)$ if $q \rightarrow _{\mathcal {B}}r$ and $p \in Q$, and
- $$(p_1,q_1) \,\wedge \, \dots \,\wedge \, (p_n,q_n) \rightarrow (p,q)$$ if $$f(p_1,\dotsc ,p_{n}) \rightarrow _{\!\mathcal {A}}p$$ and $$f(q_1,\dotsc ,q_{n}) \rightarrow _{\mathcal {B}}q$$.

These Horn clauses correspond directly to Fig. 3 with $p \leadsto q$ replaced by (*p*, *q*). It is easy to see that the resulting $\overline{\mathcal {H}}$ satisfies $(p,q) \in \overline{\mathcal {H}}$ if and only if $p \leadsto q$.

We have formalized an abstract marking algorithm for positive Horn inference systems. In order to use this algorithm, the user has to provide implementations for two building blocks, $\mathcal {H}_0$ and $\mathcal {H}_1$, which are given by

$$\begin{aligned} \mathcal {H}_0&= \{\beta \mid \top \rightarrow \beta \in \mathcal {H}\} \\ \mathcal {H}_1(\alpha ,B)&= \{\beta \mid \alpha _1 \wedge \dots \wedge \alpha _n \rightarrow \beta \in \mathcal {H}\text { and }\alpha \in \{\alpha _1,\dotsc ,\alpha _{n}\} \subseteq B \cup \{\alpha \}\} \end{aligned}$$

In essence, $\mathcal {H}_0$ computes inferences without premises, whereas $\mathcal {H}_1(\alpha ,B)$ provides all possible conclusions involving a particular premise $\alpha$ together with other premises fulfilled by *B*. These two ingredients are sufficient to implement a simple marking algorithm:

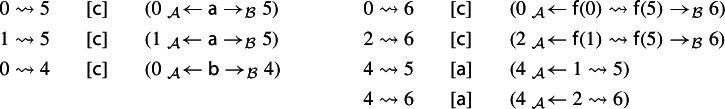

Most of the work is performed by saturate_rec, whose purpose is to add a newly inferred atom $\alpha$ to an accumulator *I* of previously inferred atoms, taking into account all further inferences that can be made using $\alpha$ and elements of *I*. It relies on $\mathcal {H}_1$ for computing the set of atoms that can be inferred using $\beta$ at least once and elements of *I* for other premises. The main method $\texttt {saturate}$ iterates over the elements of $\mathcal {H}_0$ and adds them to the accumulator *I* using the saturate_rec helper, starting with $I = \varnothing$. We formalized soundness of $\texttt {saturate}$, and of refinements to lists and finite sets.

#### Example 34

Continuing from Example 33, we note that the computation of $\mathcal {H}_0$ and $\mathcal {H}_1$ can often be done efficiently without ever computing the full set $\mathcal {H}$. For the inference rules from Fig. 3, we obtain the following descriptions:

$$\begin{aligned} \mathcal {H}_0&= \{(p,q) \mid f \rightarrow _{\!\mathcal {A}}p \text { and }f \rightarrow _{\mathcal {B}}q\} \\ \mathcal {H}_1((p,q),B)&= \{(r,q) \mid p \rightarrow _{\!\mathcal {A}}r\} \cup \{(p,r) \mid q \rightarrow _{\mathcal {B}}r\} \cup \mathcal {H}_1' \end{aligned}$$

where $\mathcal {H}_1'$ consists of all pairs $(p',q')$ such that

$$\begin{aligned} f(p_1,\dotsc ,p_{n})&\rightarrow _{\!\mathcal {A}}p'&f(q_1,\dotsc ,q_{n})&\rightarrow _{\mathcal {B}}q' \end{aligned}$$

with $(p_i,q_i) \in B \cup \{(p,q)\}$ for all $1 \leqslant i \leqslant n$, and $(p,q) = (p_i,q_i)$ for some $1 \leqslant i \leqslant n$. This last component is slightly complicated (but not much more complicated than the definition of $\mathcal {H}$ itself). On the other hand, the first two components of $\mathcal {H}_1$ make no reference to *Q*, which is a welcome simplification.

Isabelle/HOL has a *predicate compiler* [5] that produces executable code for certain inductive sets, but it is quite restricted; basically, it works by searching all possible derivation trees to arrive at a conclusion. This easily leads to non-termination when there are infinitely many such trees, which often happens. For example, using the rules in Fig. 3, if we want to check whether $1 \leadsto 2$ and there is an $\varepsilon$-transition $1 \rightarrow _{\!\mathcal {A}}1$, then the first inference rule is a possible candidate for the last inference step, leading us to check $1 \leadsto 2$ recursively, ad infinitum.

In our formalization, GTT compositions and GTT transitive closure are implemented on top of positive Horn inference. The other building blocks are derived directly from the definitions, using automatic and some manual refinement to obtain concrete implementations.

This concludes the first part. In the remainder of this section details of the second part are discussed [33]. We use the FOL-Fitting library [4], which is part of the Archive of Formal Proofs, to connect the first-order theory of rewriting and first-order logic. The translation is more or less straightforward. We interpret $\textsf{RR}_1$ constructions as predicates and $\textsf{RR}_2$ constructions as relations in first-order logic and prove both interpretations to be semantically equivalent: 
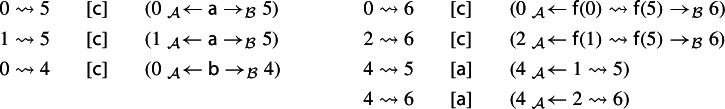


Last but not least we define the important function check_certificate which takes as input a signature, a list of TRSs, a Boolean, a formula, and a certificate. This function first verifies that the given formula and the claim corresponds to the ones referenced in the certificate and afterwards checks the integrity of the certificate. The following lemmata, which are formally proved in Isabelle, state the correctness of the check_certificate function: 
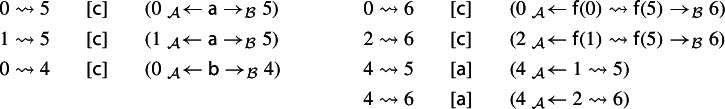


The first lemma ensures that our check function verifies that the provided parameters *fm* (formula) and *A* (answer satisfiable/unsatisfiable) match the formula and the claim stated in the certificate. The second lemma is the key result. It states that the check function returns Some True if and only if the certificate is correct. The only-if case is hidden in the last two lines. More precisely, if the claim of the certificate is wrong then negating the claim (the first-order theory of rewriting is complete) leads to a correct certificate. Therefore, if our check function returns Some None then the certificate is correct after negating the claim.

Our check function returns None if the global assumptions (the input TRS is not linear variable-separated, the signature is not empty, etc.) are not fulfilled. We plan to extend the check_certificate function in the near future such that it reports these kinds of errors.

A central part of the formalization is to obtain a trustworthy decision procedure to verify certificates. Hence we use the code generation facility of Isabelle/HOL to produce an executable version of our check_certificate function. Isabelle’s code generation facility is able to derive executable code for our constructions with the exception of inductively defined sets. We use the abstract Horn inference system framework of Definition 19 to obtain executable code for the following constructions defined as inductive sets:

- reachable and productive states of a tree automaton,
- states of tree automata obtained by the subset construction,
- $\varepsilon$-transitions for the composition and transitive closure constructions of (anchored) GTTs,
- an inductive set needed for the tree automaton for the infinity predicate.

At this point we can use Isabelle’s code generation to obtain an executable check function. The resulting code-generated certifier is called FORTify.

The overall design of FORTify is shown in the bottom half of Fig. 7. It can be viewed as two separate modules A and B. Module B is the verified Haskell code base that is generated by Isabelle’s code generation facility, containing the check_certificate function and the data type declarations for formulas and certificates. To use this functionality, we wrote a parser which translates strings representing formulas (signatures, TRSs, certificates) to semantically equivalent formulas (signatures, TRSs, certificates) represented in the data types obtained from the generated code. This was done in Haskell and refers to module A in Fig. 7. Module A accepts formulas in FORT syntax. Hence it also applies the conversion to the de Bruijn representation. After the translation in module A, the check_certificate function in module B is executed and its output is reported.

Importantly, the code in module A is not verified in Isabelle. Correctness of FORTify must therefore assume correctness of module A as well as the correctness of the Glasgow Haskell Compiler, which we use to generate a standalone executable from the generated code.

Table  lists some statistics of the underlying formalization.

**Table 4:** Formalization statistics

Topics	Lines	Facts	Defs
Utility files	1892	187	19
Terms, context, and rewriting	3969	454	97
Horn inference system	462	39	17
Tree automata	2891	319	66
Regular relations	4016	285	65
Primitives and context closure	4043	318	43
FORT decision procedure	2023	107	60
Signature extension	2874	182	15
Implementation files	3058	190	81
Total	25,228	2081	463

### Synthesis Mode

FORT can be used to synthesize TRSs that satisfy properties given by the user (which is different from finding witnessing terms in formulas as described in Sect. 7.1). This is useful for finding counterexamples and non-trivial TRSs for exam exercises as well as competitions. The synthesis procedure for a given signature $\mathcal {F}$ boils down to generating candidate TRSs and then checking the given property as shown in Fig. . The latter is done using a call to the decision procedure $\texttt {decide}(\mathcal {F},\, \varphi ,\, C)$, which checks if the formula $\varphi$ holds for the system *C* over the domain $\mathcal {T}(\mathcal {F})$. To limit and control the search space we introduce the parameters *r*, *R*, *D* and *v*:

- *r* and *R* specify the lower and upper bound on the number of rewrite rules,
- *D* specifies the upper bound on the height of the left- and right-hand sides of the rules,
- *v* specifies the number of different variables that may appear in the rewrite rules.

**Fig. 10:**
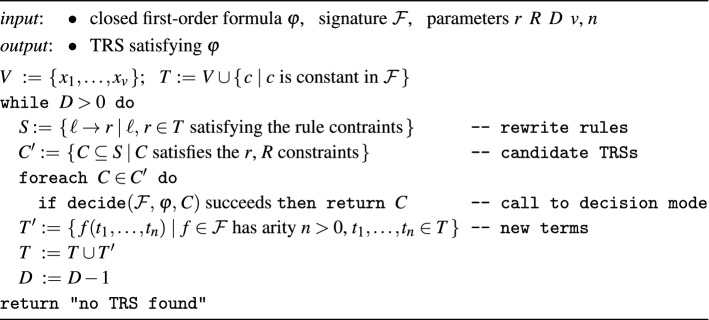
Simplified synthesis procedure (for a fixed signature)

By default the procedure searches for left-linear right-ground TRSs, but can also synthesize linear variable-separated systems. This affects the generation of candidate TRSs *S* in Fig. 10.

To extend the functionality and improve performance, the implementation in the synthesis tool (FORT-s) differs from the procedure in Fig. 10. Since the greatest cost when running the procedure comes from executing the decision procedure, care is taken to not generate and check equivalent system more than once. To this end, we keep track of fresh terms from previous iterations and only generate rules containing at least one new term, and the fresh terms in $T'$ must contain at least one new term in an argument position. Similar improvements are used when generating the rewrite systems. The second major performance improvement is the possibility of checking systems in parallel.

It is of interest to synthesize TRSs that depend on one or more other TRSs. This can be done by passing additional TRSs to FORT-s in addition to a formula which references multiple systems. The additional systems are then also passed to the decision procedure. For example, if we want to transform our leading TRS $\mathcal {R}$ (see Example 1) into an equivalent complete TRS (on ground terms), we pass both $\mathcal {R}$ and the formula

$$\begin{aligned} (\textsf{GWCR}_{0} \wedge \textsf{SN}_{0}) \wedge \forall \,s\,\forall \,t\,(s \leftrightarrow ^{*}_{0} t \iff s \leftrightarrow ^{*}_{1} t) \end{aligned}$$

to FORT-s. Here the index 1 refers to $\mathcal {R}$ and the index 0 to the system to be synthesized. This returns the TRS consisting of the rules

$$\begin{aligned} \textsf{a}&\rightarrow \textsf{b}&\textsf{f}(\textsf{b})&\rightarrow \textsf{g}(\textsf{a},\textsf{a})&\textsf{g}(\textsf{b},\textsf{b})&\rightarrow \textsf{a} \end{aligned}$$

Using formulas referencing multiple TRSs FORT-s can also be used to synthesize multiple systems.

For convenience FORT-s supports multiple ways to specify the signature used during synthesis. The full user interface of FORT-s is given in Appendix C.

### Undecidability of Synthesis

Since the first-order theory is decidable for linear variable-separated TRSs a natural question arises. Is synthesis also decidable for these systems? In other words, can we determine if there exists a linear variable-separated TRS satisfying a given property? Unfortunately this is not the case.

#### Theorem 17

The following problem is undecidable:
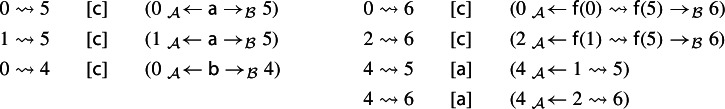


#### Proof

We show the undecidability by a reduction from Post correspondence problem. Let *P* be a finite set of pairs of non-empty strings over the alphabet $\{0,1\}$. We define a formula $\varphi _P$ in the first-order theory of rewriting that is satisfiable if and only if *P* has a solution. To this end, consider the following predicates:

$$\begin{aligned} \textsf{node}(x)&~:=~ x \rightarrow x \\ \textsf{next}(x,y)&~:=~ \textsf{node}(x) \wedge \textsf{node}(y) \wedge x \rightarrow y \wedge x \ne y \\ \textsf{step}&~:=~ \forall x\,\bigl (\textsf{node}(x) \wedge x \ne e \implies \exists \,y~\textsf{next}(x,y)\bigr ) \\ \textsf{unique}&~:=~ \forall x\,\forall y\,\forall z \bigl (\textsf{next}(x,y) \wedge \textsf{next}(x,z) \implies y = z\bigr ) \\ \textsf{linear}&~:=~ \textsf{step} \wedge \textsf{unique} \\ \textsf{value}(x,0)&~:=~ x \rightarrow a \wedge \lnot (x \rightarrow b) \\ \textsf{value}(x,1)&~:=~ x \rightarrow b \wedge \lnot (x \rightarrow a) \\ \textsf{finite}&~:=~ \lnot \exists \,x~\textsf{INF}_{\ne }(x) \end{aligned}$$

Positions in a solution string are represented by *nodes*, which are linearly ordered. Nodes are characterized by self-loops. The special nodes *s* and *e* mark the starting and final positions in a solution of *P*. The predicate *finite* ensures that solution strings are finite. We have two additional elements, *a* and *b* that correspond to the symbols 0 and 1.

$$\begin{aligned} \textsf{border}(x,y)&~:=~ \textsf{node}(x) \wedge \textsf{node}(y) \wedge \exists \,z\, (\lnot \textsf{node}(z) \wedge x \rightarrow z \wedge z \rightarrow y) \end{aligned}$$

The *border* predicate marks the two positions in a solution string corresponding to the decomposition into first and second components. The latter is checked by the *solution* predicate:The formula $\varphi _P$ is now defined as

$$\begin{aligned} \exists \,s\,\exists \,e\,\exists \,a\,\exists \,b\, \bigl (s \ne e \wedge \textsf{border}(s,s) \wedge \textsf{linear} \wedge \textsf{finite} \wedge \textsf{solution}\bigr ) \end{aligned}$$

$$\square $$

Note that the witnessing TRSs constructed in the above proof are actually abstract rewrite systems (ARSs) that consist of rewrite rules between constants. The construction is illustrated in Fig. , for the PCP instance $P = \{(1,011),(10,11),(001,00)\}$ with solution $001|10|001|1 = 00|11|00|011$. The separation bars correspond to the elements $b_1$, $b_2$ and $b_3$. Node $n_9$ witnesses *e*. Elements 0 and 1 witness *a* and *b*.

**Fig. 11:**
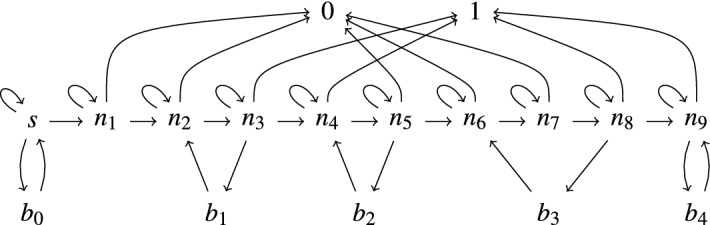
The construction for PCP instance *P*

The synthesis problem is obviously decidable for ARSs over a fixed signature, but remains undecidable for TRSs over a fixed signature, since we can still generate an arbitrary number of ground terms using non-constant function symbols. Take for example the signature $\{\textsf{E}, \textsf{s}, \textsf{0}\}$, where $\textsf{E}$ and $\textsf{s}$ are unary function symbols and $\textsf{0}$ is a constant. We can then represent an arbitrary number *n* of objects (nodes, borders and values in the encoding) using ground terms of the shape $\textsf{E}(\textsf{s}^n(\textsf{0}))$. The rules of the ARS correspond to rules between such ground terms of the generated TRS. (The inclusion of the function symbol $\textsf{E}$ removes any possibility of unwanted overlap between rules of the TRS.)

## Experiments

In this section we describe the experiments we performed with FORT-h, FORT-s, and FORTify. We include version 1.0 of FORT-h, which was first published as part of an artifact^5^ in conjunction with [42]. The current version of FORT-h is 2.0. Full details of the experiments are available from the website^6^ accompanying this paper. Precompiled binaries of FORT-h 2.0, FORT-s, and FORTify are available from the same site. All experiments were run on a computer equipped with an Intel Core i7-5930K processor with 6 cores, and with 32 GB of memory. To remove any ambiguity in the calls made to the tools we use FORT-syntax (see Appendix A) to specify formulas in this section. This also aids in replicating the experiments.

### FORT-h and FORTify

For the experiments reported in this section we used a timeout of 60 s for the decision tools and 600 s for FORTify.

#### Comparing Different Representations of Properties

The problems for these experiments are taken from the Confluence Problems database (COPS),^7^ and consists of 122 left-linear right-ground TRSs. The formulas were taken from the experiments reported in [46].

##### Experiment 1

The first three

15 $$ \begin{aligned} \texttt {"forall s, t, u }&\texttt {(s ->* t  \&  s ->* u => t join u)"} \end{aligned}$$

16 $$ \begin{aligned} \texttt {"forall s, t, u }&\texttt {(s ->* t  \&  s -> u => t join u)"} \end{aligned}$$

17 $$\begin{aligned} \texttt {"forall t, u }&\texttt {(t <->* u => t join u)"} \end{aligned}$$

denote different but equivalent formulations of ground-confluence ($\textsf{GCR}$). The results are shown in Table , where the YES (NO) column shows the number of systems determined to be (non-)ground-confluent together with average time ($\varnothing$-time) the tool took. The $\infty$ column is the number of timeouts. To compare overall performance the *total time* column contains the sum of all run times, including timeouts but excluding the time taken by FORTify. The ✔ columns show the numbers of certifiable results as well as the overall time taken by FORTify. These results show that, even though they have the same meaning, the choice of formula has an impact on performance. Most notably this can be seen when comparing the number of solved problems by FORT-h 2.0. The formula (16) (semi-confluence) was fastest with no timeouts, followed by (15) with one timeout and (17) with two. It is apparent that formulas containing conversion ($\leftrightarrow _{}^{*}$) are especially slow, which we will also see in later experiments. Further note that FORT-h 2.0 can solve an additional problem compared to the 1.0 version, for each formula.

**Table 5:** FORT-h (with FORTify) and FORT-j run on $\textsf{GCR}$ formulas

		YES	$\varnothing$-time	✔	NO	$\varnothing$-time	✔	$\infty$	total (✔) time	
(15)	FORT-h 2.0	37	0.89 s	37	84	0.69 s	81	1	151.12 s	(0.8 h)
	FORT-h 1.0	36	0.26 s	10	84	0.56 s	16	2	176.23 s	(17.6 h)
	FORT-j	37	0.31 s	–	82	0.52 s	–	3	234.08 s	
(16)	FORT-h 2.0	38	1.50 s	37	84	0.06 s	81	0	62.13 s	(0.9 h)
	FORT-h 1.0	37	1.48 s	10	84	0.09 s	16	1	122.55 s	(17.8 h)
	FORT-j	37	0.32 s	–	82	0.50 s	–	3	233.20 s	
(17)	FORT-h 2.0	37	0.91 s	37	83	0.04 s	81	2	156.64 s	(1.0 h)
	FORT-h 1.0	36	0.45 s	6	83	0.08 s	9	3	202.64 s	(18.2 h)
	FORT-j	37	0.32 s	–	82	0.55 s	–	3	236.69 s	

Interestingly FORT-h (2.0) is generally faster and can solve more problems than FORT-j even though the latter implements parallelism. This performance advantage is more prominent in systems which are non-confluent where FORT-h can solve more problems, while for problems with the answer YES, FORT-j can solve close to the same number of problems, while taking less time per problem in general. The table also shows that FORTify can certify most of the results, which is a large improvement over the previous version. Here the difference between the three formulas is not as visible, but it is also faster on (16) and (15), and slowest on (17). The times for FORTify must also be seen in the context that it ran on more problems on the first two formulas, since FORT-h could produce more certificates. No wrong results by the decision tools where identified.

##### Experiment 2

The second set of formulas represents the normal form property, restricted to ground terms ($\textsf{GNFP}$):

18 $$ \begin{aligned} \texttt {"forall t, u }&\texttt {(t <->* u  \&  NF(u) => t ->* u)"} \end{aligned}$$

19 $$ \begin{aligned} \texttt {"forall s, t, u }&\texttt {(s -> t  \&  s ->! u => t ->* u)"} \end{aligned}$$

20 $$\begin{aligned} \texttt {"forall t }&\texttt {(WN(t) => CR(t))"} \end{aligned}$$

The results for these are shown in Table . The same pattern is observed, where even though all three can (dis)prove satisfaction for the same formulas, FORT-h 2.0 is faster than FORT-j overall, and has improved over FORT-h 1.0.

**Table 6:** FORT-h (with FORTify) and FORT-j run on $\textsf{GNFP}$ formulas

		YES	$\varnothing$-time	✔	NO	$\varnothing$-time	✔	$\infty$	Total (✔) time	
(18)	FORT-h 2.0	59	0.30 s	57	63	0.04 s	63	0	20.37 s	(0.5 h)
	FORT-h 1.0	59	0.70 s	31	63	0.07 s	20	0	45.62 s	(14.6 h)
	FORT-j	59	0.23 s	–	63	0.39 s	–	0	38.16 s	
(19)	FORT-h 2.0	59	0.02 s	59	63	0.01 s	63	0	1.76 s	(0.1 h)
	FORT-h 1.0	59	0.03 s	46	63	0.01 s	50	0	2.55 s	(6.3 h)
	FORT-j	59	0.22 s	–	63	0.30 s	–	0	31.83 s	
(20)	FORT-h 2.0	59	0.03 s	56	62	0.11 s	62	1	68.83 s	(0.8 h)
	FORT-h 1.0	59	0.05 s	42	62	0.12 s	45	1	70.51 s	(8.6 h)
	FORT-j	59	0.31 s	–	62	0.64 s	–	1	117.86 s	

Since the representations containing conversion ($\leftrightarrow _{}^{*}$) in the previous experiments are outperformed by the other representations, it is often a good idea to avoid it. Obviously this is not always possible. Take the properties UNC, CE or consistency for example. It is therefore important to choose the correct representation in the primitive automata constructions, to ensure good performance when conversion cannot be avoided.

##### Experiment 3

We tested the following three representations of conversion for a TRS $\mathcal {R}$:212223The representation (21) is the one listed in Table 2. Using composition () instead of union as in (22) works becauseThe third representation (23) uses the identity ${\rightarrow _{\mathcal {R}\cup \mathcal {R}^-}^{\varepsilon }} = {\leftrightarrow _{\mathcal {R}}^{\varepsilon }}$ and is the default used by FORT-h. The results of running FORT-h 2.0 on the COPS dataset, using the formula  for consistency with the three different representations of conversion can be seen in Table . We can see that (23) is the fastest with and overall runtime of 19.31 s. It is about 12% faster than (21) and about 20% faster than (22). Also important is that (23) produces smaller automata, which leads to better performance when conversion is embedded within larger formulas. Consider for example COPS #741:

$$\begin{aligned} \textsf{if}(\textsf{true},\textsf{a},x)&\rightarrow \textsf{a}&\textsf{if}(\textsf{true},\textsf{g}(\textsf{a}),x)&\rightarrow \textsf{g}(\textsf{a})&\textsf{g}(\textsf{a})&\rightarrow \textsf{g}(\textsf{g}(\textsf{a}))\\ \textsf{if}(\textsf{true},\textsf{b},x)&\rightarrow \textsf{b}&\textsf{if}(\textsf{true},\textsf{g}(\textsf{b}),x)&\rightarrow \textsf{g}(\textsf{b})&\textsf{g}(\textsf{b})&\rightarrow \textsf{a} \\ \textsf{if}(\textsf{false},x,\textsf{a})&\rightarrow \textsf{a}&\textsf{if}(\textsf{false},x,\textsf{g}(\textsf{a}))&\rightarrow \textsf{g}(\textsf{a})&\textsf{f}(\textsf{a},\textsf{b})&\rightarrow \textsf{b} \\ \textsf{if}(\textsf{false},x,\textsf{b})&\rightarrow \textsf{b}&\textsf{if}(\textsf{false},x,\textsf{g}(\textsf{b}))&\rightarrow \textsf{g}(\textsf{b})&\textsf{f}(\textsf{g}(\textsf{g}(\textsf{a})),x)&\rightarrow \textsf{b} \end{aligned}$$

The $\textsf{RR}_{2}$ automata representing (21) and (22) both contain 233 states, 7927 transitions and 9 $\varepsilon$-transitions before trimming, and 132 states and 4937 transitions after. In comparison the automaton for (23) contains 152 states, 3975 transitions and 9 $\varepsilon$-transitions before, and 75 states with 2313 transitions after trimming. Overall (23) therefore has less than half the number of transitions in this example, which can have a significant effect in any later closure operations.

**Table 7:** FORT-h 2.0 run on   with differing encodings of conversion

	YES	$\varnothing$-time	NO	$\varnothing$-time	$\infty$	total time
(21)	91	0.10 s	31	0.42 s	0	22.00 s
(22)	91	0.10 s	31	0.48 s	0	24.22 s
(23)	91	0.07 s	31	0.41 s	0	19.31 s

The final experiment in this subsection involves the normal form predicate $\textsf{NF}(t)$, which is implemented in FORT-h according to the description in Sect. 5.4, instead of using the equivalent formula $\lnot \,\exists \,u\,(t \rightarrow u)$.

##### Experiment 4

Consider the formula "forall s (exists t (NF(t) & s ->* t))" for normalization and COPS #503:

$$\begin{aligned} \textsf{f}(\textsf{a},\textsf{a},\textsf{b},\textsf{b})&\rightarrow \textsf{f}(\textsf{c},\textsf{c},\textsf{c},\textsf{c})&\textsf{a}&\rightarrow \textsf{b}&\textsf{a}&\rightarrow \textsf{c}&\textsf{b}&\rightarrow \textsf{a}&\textsf{b}&\rightarrow \textsf{c} \end{aligned}$$

When using the formula $\lnot \,\exists \,u\,(t \rightarrow u)$ for NF(t), FORT-h first constructs the $\textsf{RR}_{2}$ automaton $\mathcal {A}_1$ for $t \rightarrow u$, with 4 states and 15 transitions. It then projects to construct the automaton $\mathcal {A}_2$ for $\exists \,u\,(t \rightarrow u)$ with 4 states and 13 transitions, and finally it has to determinize $\mathcal {A}_2$ and construct the complement for the negated formula $\lnot \,\exists \,u\,(t \rightarrow u)$, resulting in the automaton $\mathcal {A}_3$ with 4 states and 259 transitions before and 1 state with two 2 transitions after trimming. If instead the direct normal form predicate is used, FORT-h immediately produces the latter automaton, without having to construct the intermediate automata or having to trim. The impact on runtime can be seen in Table . It is rather small for FORT-h, but for FORTify the direct construction is about 13% faster. When looking at the sizes of the automata, the average untrimmed automaton $\mathcal {A}_3$, for our dataset of left-linear right-ground COPS problems, contains 75.8 transitions while the average automaton for the normal form predicate contains 13.3 transitions.

**Table 8:** FORT-h 2.0 (with FORTify) run on normalization with different encodings of $\textsf{NF}$

	YES	$\varnothing$-time	✔	NO	$\varnothing$-time	✔	$\infty$	total (✔) time	
"NF(t)"	41	0.02 s	41	81	0.00 s	81	0	0.85 s	(20.50 s)
	41	0.02 s	41	81	0.00 s	81	0	1.05 s	(23.71 s)

#### Properties Involving Multiple TRSs

We also ran experiments to test performance on properties involving two TRSs. As a dataset we constructed problems of all ordered pairs of COPS problems, resulting in 7503 pairs.

##### Experiment 5

The first property tested was ground-commutation ($\textsf{GCOM}$). The results, presented in Table , show that FORT-h is ahead of FORT-j here as well. It can (dis)prove more problems, timing-out on only two as compared to 49 problems. Additionally it does so in significantly less time. With FORTify we can see a large improvement over the old version. It is able to certify close to 98% of the results found by FORT-h 2.0.

**Table 9:** FORT-h (with FORTify) and FORT-j run on $\textsf{GCOM}$

	YES	$\varnothing$-time	✔	NO	$\varnothing$-time	✔	$\infty$	total (✔) time	
FORT-h 2.0	1381	0.10 s	1368	6120	0.02 s	5965	2	374.63 s	(51.5 h)
FORT-h 1.0	1381	0.16 s	878	6120	0.03 s	3666	2	517.32 s	(681.5 h)
FORT-j	1354	1.46 s	–	6100	0.94 s	–	49	10670.89 s	

In the 2019 edition of the Confluence Competition [41] three tools contested the commutation ($\textsf{COM}$) category:^8^ACP [2], CoLL [49], and FORT-j. On input problem COPS #1118 the tools gave conflicting answers.

##### Example 35

COPS #1118 is about the commutation of the TRSs COPS #669

$$\begin{aligned} \textsf{a}&\rightarrow \textsf{c}&\textsf{f}(\textsf{a})&\rightarrow \textsf{b}&\textsf{b}&\rightarrow \textsf{b}&\textsf{b}&\rightarrow \textsf{h}(\textsf{b},\textsf{h}(\textsf{c},\textsf{a})) \end{aligned}$$

and COPS #695

$$\begin{aligned} \textsf{h}(\textsf{a},\textsf{a})&\rightarrow \textsf{c}&\textsf{b}&\rightarrow \textsf{h}(\textsf{b},\textsf{a})&\textsf{b}&\rightarrow \textsf{a}&\textsf{f}(\textsf{c})&\rightarrow \textsf{c}&\textsf{c}&\rightarrow \textsf{a} \end{aligned}$$

To determine the correct answer we use FORT-h 2.0 to produce a certificate for ground-commutation by calling

> fort-h -c cert -i "GCom([0],[1])" 1118.trs YES

This produces the following certificate:

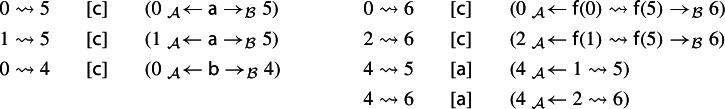

When passing this certificate to FORTify, after 0.2 s the output Certified is produced, so we can be assured that the TRSs do commute. Note that the inference steps 0 and 1 contain the optional size information. Here (size k m n) means the underlying $\textsf{RR}_n$ automaton constructed by FORT-h 2.0 contains k states, m transitions, and n $\varepsilon$-transitions.

##### Experiment 6

For the second experiment using multiple TRSs we tested FORT-h 2.0 and FORTify on conversion equivalence and normalization equivalence, once for all terms and once for only ground-terms. FORT-h 1.0 and FORT-j have not implemented the necessary signature extension results to cover these properties, and are therefore not run. The results can be seen in Table . Comparing the properties to the corresponding ground-properties, we can see that FORT-h 2.0 succeeds to find results on the same number of problems. However, six results moved from YES to NO in the case of $\mathsf {(G)CE}$ and four in the case of $\mathsf {(G)NE}$. These correspond to TRSs where the additional constants are needed to disprove the property. While the run times of FORT-h 2.0 stayed almost the same when comparing the ground and non-ground properties, we can see that FORTify does take longer to certify results on the non-ground properties. This is to be expected, since the additional constants lead to larger automata. Simply by having a larger signature, some of the atomic constructions produce more transition rules. While this is usually only a small difference it can have a significant effect when embedded within a bigger formula.

**Table 10:** FORT-h 2.0 (with FORTify) run on $\mathsf {(G)CE}$ and $\mathsf {G(NE)}$

	YES	$\varnothing$-time	✔	NO	$\varnothing$-time	✔	$\infty$	total (✔) time	
GCE	157	0.70 s	150	7162	0.94 s	6736	184	5.0 h	(125.6 h)
CE	151	0.74 s	144	7168	0.93 s	6739	184	5.0 h	(127.1 h)
GNE	181	0.02 s	181	7320	0.04 s	7308	2	448.75 s	(5.4 h)
NE	177	0.02 s	177	7324	0.04 s	7312	2	446.54 s	(5.6 h)

#### Optimizations

To show this effect, and the improvement caused by Lemma 39 consider the following example.

##### Example 36

Consider COPS #214

$$\begin{aligned} \textsf{a}&\rightarrow \textsf{b}&\textsf{a}&\rightarrow \textsf{f}(\textsf{a})&\textsf{b}&\rightarrow \textsf{f}(\textsf{f}(\textsf{b}))&\textsf{f}^{64}(\textsf{b}) \rightarrow \textsf{b} \end{aligned}$$

where $\textsf{f}^{64}$ represents 64 nested applications of $\textsf{f}$. To check $\textsf{UNC}$, FORT-h 2.0 extends the signature as needed and uses the formula for $\textsf{GUNC}$ internally represented as

$$\begin{aligned} \lnot \,\exists (\exists ((\textsf{NF}(0) \times \textsf{NF}(1)) \wedge 0 \ne 1 \wedge 0 \leftrightarrow _{}^{*}1)) \end{aligned}$$

In this case no constants have to be added, since the TRS is ground. The intermediate automaton $\mathcal {A}_1$ for the subformula $\textsf{NF}(0) \times \textsf{NF}(1)$ contains no transitions, since the TRS has no normal forms for the given signature. For the automaton $\mathcal {A}_2$ of $0 \ne 1$ we have 13 transitions and $\mathcal {A}_3$ for $0 \leftrightarrow _{}^{*}1$ has 150,569 transitions. Like we have seen in earlier experiments, the automaton for conversion is clearly the largest, and would also take the largest amount of time to construct. However, since $\mathcal {A}_1$ is empty, the intersection with $\mathcal {A}_2$ and then further with $\mathcal {A}_3$ will also be empty. And due to the lazy evaluation strategy of Haskell the third automaton will never be constructed. Therefore FORT-h 2.0 can almost instantly (0.01 s) determine that the automaton for the formula within the negation is empty, and conclude that $\textsf{UNC}$ holds. However, if we were to ignore the optimization introduced by Lemma 39 and add two constants the automaton $\mathcal {A}_1$ is no longer empty, since we added two normal forms to the domain. This changes the numbers as follows: The automaton $\mathcal {A}_1$ would contain 15 transitions and 3 states, $\mathcal {A}_2$ has 31 transitions and 3 states, and $\mathcal {A}_3$ has 150,571 transitions and 4356 states. Since none of the automata are empty we must construct the intersection $\mathcal {A}_1 \cap \mathcal {A}_2$ containing 34 transitions and 6 states. After trimming this drops to 20 transitions and 4 states. The intersection $(\mathcal {A}_1 \cap \mathcal {A}_2) \cap \mathcal {A}_3$ then results in an automaton with 132,652 transitions and 8584 states. Only after trimming we see that this automaton is empty to conclude that $\textsf{UNC}$ holds. Overall this takes FORT-h 2.0 7.15 s, which is orders of magnitude slower than with the optimization. While such large speedups are not the norm, the overall runtime on the COPS dataset for $\textsf{UNC}$ drops by about 16%, as seen in Table .

**Table 11:** FORT-h 2.0 run on $\textsf{UNC}$ with and without Lemma 39

		YES	$\varnothing$-time	NO	$\varnothing$-time	$\infty$	total time
"UNC"	(with Lemma 39)	72	0.29 s	49	0.20 s	1	90.92 s
"{+2} GUNC"	(two constants)	72	0.54 s	49	0.20 s	1	108.52 s

**Fig. 12:**
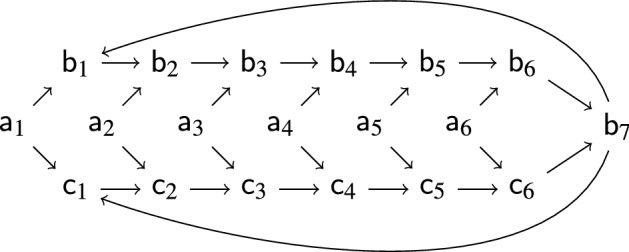
Graph presentation of COPS #116

##### Example 37

To see that the optimization of collapsing strongly connected states, introduced in Sect. 7.1, can have a significant effect consider COPS #116. It is an ARS consisting of 26 rules presented as a graph in Fig. . To check if it is consistent we can use the formula  which is internally represented as $\exists (\exists (\lnot \,(0 \leftrightarrow _{}^{*}1)))$. For this FORT-h constructs the automaton $\mathcal {A}$ for $0 \leftrightarrow _{}^{*}1$, consisting of 8 states 418 transitions and 3 $\varepsilon$-transitions. After eliminating the $\varepsilon$-transitions and trimming, we are left with 1 state and 361 transitions. The complement automaton $\mathcal {A}^c$ which represents $\lnot (0 \leftrightarrow _{}^{*}1)$ has the same size, which drops to zero after trimming, showing that the system is not consistent. Overall FORT-h takes 0.34 s.

If we however remove the optimization and do not collapse strongly connected components, we get significantly larger automata. The automaton $\mathcal {A}$ grows to 8427 states, 2827 transitions and 851,916 $\varepsilon$-transitions. At this point the procedure usually eliminates the $\varepsilon$-transitions and trims the automaton, but FORT-h does not manage to do so within the 60 s timeout. The overall improvement on testing consistency can be seen in Table .

**Table 12:** FORT-h 2.0 run on  with/out collapsing SCCs

	YES	$\varnothing$-time	NO	$\varnothing$-time	$\infty$	Total time
Collapsing SCCs	91	0.07 s	31	0.41 s	0	19.31 s
Unoptimized	91	0.14 s	28	1.21 s	3	223.82 s

#### Comparison with Other Tools

As a last experiment we compare FORT-h to a number of state of the art tools. For the properties $\textsf{GCR}$, $\textsf{NFP}$, $\textsf{UNC}$, $\textsf{UNR}$ and $\textsf{COM}$ we chose the following tools that competed in the corresponding categories in the confluence competition in 2021: ACP [2] in $\textsf{UNC}$ and $\textsf{COM}$, AGCP [1] in $\textsf{GCR}$, CSI [44] in $\textsf{NFP}$, $\textsf{UNC}$ and $\textsf{UNR}$, and CoLL [49] in $\textsf{COM}$. All these tools implement various sufficient conditions for the corresponding property and are not limited to linear variable-separated or left-linear right-ground TRSs. For the sake of comparing them to FORT-h we run them only on the left-linear right-ground TRSs in COPS, and on the pairs of these problems for $\textsf{COM}$. The results can be seen in Table .

We can see that FORT-h 2.0 significantly outperforms all the other tools on this class of systems. For all properties it can find results for more problems and can often do so with less time per problem. This difference is especially pronounced in the $\textsf{COM}$ category, where FORT-h 2.0 can (dis)prove all but three of the 7503 problems, while ACP and CoLL timeout or return Maybe on more than 2000 of these. Given this performance discrepancy it is of interest to other tools to use FORT-h 2.0 on problems of this class. Here it could be used as a black box on problems (or subproblems) as long as they are linear variable-separated TRSs, and can be expressed in the first-order theory of rewriting. An example of such a tool is CONFident [27] which uses FORT, among other tools, as part of its procedure.^9^

**Table 13:** FORT-h 2.0 compared to other tools

		YES	$\varnothing$-time	NO	$\varnothing$-time	$\infty$/MAYBE	Total time
$\textsf{GCR}$	FORT-h 2.0	37	0.06 s	84	0.04 s	1	65.82 s
	AGCP	24	0.02 s	79	0.07 s	19	276.42 s
$\textsf{NFP}$	FORT-h 2.0	55	0.02 s	67	0.01 s	0	1.76 s
	CSI	55	0.79 s	61	1.02 s	6	186.94 s
$\textsf{UNC}$	FORT-h 2.0	72	0.31 s	49	0.21 s	1	92.75 s
	ACP	70	0.08 s	47	0.86 s	5	345.91 s
	CSI	71	0.83 s	46	1.12 s	5	187.37 s
$\textsf{UNR}$	FORT-h 2.0	96	0.02 s	26	0.01 s	0	2.21 s
	CSI	86	0.81 s	26	0.76 s	10	209.12 s
$\textsf{COM}$	FORT-h 2.0	1365	0.10 s	6135	0.04 s	3	578.3 s
	CoLL	1349	0.21 s	4015	0.13 s	2139	19.5 h
	ACP	1238	0.01 s	3519	0.04 s	2746	5.0 h

Another interesting point can be seen when comparing the first line in Table 13, where 37 YES results are reported, with the fourth line in Table 5, where 38 YES results are reported. Both formulas check ground-confluence, but the built-in $\textsf{GCR}$ property is represented slightly different. Instead of the joinability predicate ($t \downarrow u$), which is constructed via operations on anchored GTTs, it uses the equivalent formula $\exists \,v\,(t \rightarrow _{}^{*} v \wedge u \rightarrow _{}^{*} v)$. In this case the explicit formula is slower on COPS #215 leading to the additional timeout, but is faster on other problems causing the total time to be similar. Like previous experiments this shows that the representation of a property can have a large and non-obvious effect on performance.

### FORT-s

In this subsection we report on the synthesis experiments that we performed. All experiments were executed with the options -j 4 and +RTS -A64M, unless stated otherwise. First we consider Fig. 6.

#### Experiment 7

The following TRSs were produced by FORT-s on the given formulas when restricting the signature (using the command-line option -S "a 0 b 0 f 2") to a binary function symbol $\textsf{f}$ and two constants $\textsf{a}$ and $\textsf{b}$:We do not know whether there exist TRSs over the restricted signature that satisfy . Human expertise was used to produce a witness over a larger signature, which was subsequently simplified using the decision mode of FORT:

$$\begin{aligned} \textsf{b}&\rightarrow \textsf{a}&\textsf{c}&\rightarrow \textsf{c}&\textsf{d}&\rightarrow \textsf{c}&\textsf{f}(x,\textsf{a})&\rightarrow \textsf{A}&\textsf{f}(x,\textsf{A})&\rightarrow \textsf{A} \\ \textsf{b}&\rightarrow \textsf{c}{} & {} {}&\textsf{d}&\rightarrow \textsf{e}&\textsf{f}(x,\textsf{e})&\rightarrow \textsf{A}&\textsf{f}(\textsf{c},x)&\rightarrow \textsf{A} \end{aligned}$$

FORT-h produces the following terms as witnesses for the fact that $\textsf{UNR}$ is not satisfied: $t = \textsf{A}$ and $u = \textsf{f}(\textsf{e},\textsf {\$})$. Indeed both $\textsf{A}$ and $\textsf{f}(\textsf{e},\textsf {\$})$ are normal forms reachable from $\textsf{f}(\textsf{d},\textsf {\$})$. Moreover, we obtain witnesses $t = \textsf{a}$ and $u = \textsf{e}$ showing that $\textsf{GUNC}$ does not hold. (The rule $\textsf{c} \rightarrow \textsf{c}$ is needed to satisfy $\textsf{GUNR}$.)

In the next experiment we use the infinity predicate to distinguish well-known subclasses of linear-variable separated TRSs.

#### Experiment 8

The formula

$$\begin{aligned}&\exists \,t~\textsf{INF}_{\xleftarrow {\varepsilon }}(t){} & {} \texttt {"exists t (INF(e<-,t))"} \end{aligned}$$

distinguishes ground TRSs from left-linear right-ground (but not ground) ones. Without any options FORT-s produces the TRS $\{\textsf{g}(x) \rightarrow \textsf{g}(\textsf{a})\}$ in a fraction of a second. The formulais true for TRSs that are not ARSs. FORT-s produces the empty TRS over the signature consisting of the constant $\textsf{a}$ and an additional constant and unary function symbol. The second constant is not necessary, but is added by the signature step. Finally, to distinguish linear variable-separated TRSs from left-linear right-ground TRSs, assuming the signature contains at least one non-constant function symbol, the formula

$$\begin{aligned}&\exists \,t~\textsf{INF}_{\xrightarrow {\varepsilon }}(t){} & {} \texttt {"exists t (INF(->e,t))"} \end{aligned}$$

can be used in connection with the -l option. This generates the TRS $\{\textsf{a} \rightarrow x\}$ over the signature consisting of the constant $\textsf{a}$ and an additional constant and unary function symbol. Without the latter, the generated linear variable-separated TRS induces only a finite rewrite relation. Adding "& CR & WN" to the last formula produces the TRS $\{\textsf{a} \rightarrow \textsf{b}, \textsf{f}(\textsf{b}) \rightarrow x\}$.

#### Experiment 9

Finding a locally confluent but not confluent TRS $\mathcal {R}$ is easy. FORT-s produces the ground TRS

$$\begin{aligned} \textsf{a}&\rightarrow \textsf{b}&\textsf{f}(\textsf{a})&\rightarrow \textsf{a}&\textsf{a}&\rightarrow \textsf{f}(\textsf{a}) \end{aligned}$$

when given the formula  is less than 1 s. The well-known abstract counterexample by Kleene
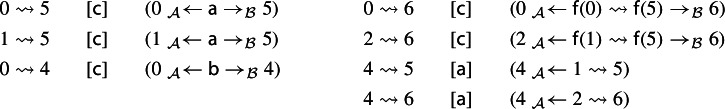
 is found by restricting the search to ARSs. The easiest way to do this is with the option -A 0, which sets the maximal arity of function symbols to 0. Moreover, the maximum number of rewrite rules has to be set to at least four (-R 4). If we impose the additional condition that $\mathcal {R}^-$ is terminating (cf. [56]), the TRS

$$\begin{aligned} \textsf{a}&\rightarrow \textsf{b}&\textsf{a}&\rightarrow \textsf{g}(\textsf{a})&\textsf{b}&\rightarrow \textsf{g}(\textsf{g}(\textsf{b})) \end{aligned}$$

is generated withwithout any additional command-line options in less than 7 s.

The next experiment shows how FORT-s can be used to complete TRSs into complete (canonical) ones.

#### Experiment 10

FORT-s produces the TRS $\{\textsf{a} \rightarrow \textsf{c}, \textsf{f}(x) \rightarrow \textsf{a}\}$ when presented the formula

$$  \begin{aligned}&\texttt {"[0](WCR  \&  SN)  \&  forall s, t ([0] s<->* t<=> [1] s <->* t)"} \end{aligned}$$

with input.trs as additional parameter. Here input.trs consists of the three rules

$$\begin{aligned} \textsf{c}&\rightarrow \textsf{a}&\textsf{f}(\textsf{b})&\rightarrow \textsf{c}&\textsf{f}(\textsf{c})&\rightarrow \textsf{a} \end{aligned}$$

The result is complete (as demanded by "[0](WCR & SN)"), but not equivalent! The reason is that "forall s, t ([0] s <->* t <=> [1] s <->* t)" ensures ground conversion equivalence, and we have seen in Sect. 6 that an extra constant is needed to reduce conversion equivalence to ground conversion equivalence. The same behaviour can also be seen for our leading example, where the same formula is used. When presented the formula

$$  \begin{aligned}&\texttt {"[0](WCR  \&  SN)  \&  CE([0],[1])"} \end{aligned}$$

the equivalent complete TRS consisting of the rules

$$\begin{aligned} \textsf{a}&\rightarrow \textsf{c}&\textsf{f}(\textsf{b})&\rightarrow \textsf{f}(\textsf{a})&\textsf{f}(\textsf{c})&\rightarrow \textsf{a} \end{aligned}$$

is synthesized. Note that the latter TRS is not canonical since not all right-hand sides are in normal form. It is well-known that every system of ground equations admits a presentation as canonical TRS. Snyder [50] proved that a ground TRS is canonical if only if it is *reduced*. The latter property is easily expressible:Together with "CE([0],[1])", any ground TRS is transformed into an equivalent canonical one, without explicitly requiring confluence and termination. For our example TRS, we obtain

$$\begin{aligned} \textsf{a}&\rightarrow \textsf{c}&\textsf{f}(\textsf{b})&\rightarrow \textsf{c}&\textsf{f}(\textsf{c})&\rightarrow \textsf{c} \end{aligned}$$

The final experiment is based on [57, Example 5.1] and shows how FORT-s can be used to synthesize multiple TRSs.

#### Experiment 11

If we want to generate two terminating ARSs such that their union is non-terminating, the formula  can be used in connection with the options -A 0 and -n 2. The latter tells FORT-s to synthesize two TRSs. The additional requirement that the composition of both relations is a subset of the transitive closure of one of them is expressed as

$$ \begin{aligned}&\texttt {"forall s, t, u ([0] s -> t  \&  [1] t -> u => [0]} \\&\quad \texttt { s ->+ u | [1] s ->+ u)"} \end{aligned}$$

In a fraction of a second FORT-s synthesizes the following two ARSs satisfying the conjunction of these requirements:

$$\begin{aligned} \mathcal {A}_0: \quad \textsf{a}&\rightarrow \textsf{b} \qquad \textsf{b} \rightarrow \textsf{c}&\mathcal {A}_1: \quad \textsf{b}&\rightarrow \textsf{c} \qquad \textsf{c} \rightarrow \textsf{a} \end{aligned}$$

Using completely different techniques, similar ARSs are generated by Carpa, the tool described in Zantema [57].

## Conclusion

In this paper we presented a formalized decision procedure of the first-order theory of rewriting for the class of linear variable-separated TRSs. The decision procedure ultimately goes back to Dauchet and Tison [10] and is the basis of the tool FORT-h. Different from [8, 10], we extensively use *anchored* GTT relations. These have better closure properties than GTT relations and allow to efficiently express numerous binary relations on ground terms, easing formalization efforts. We presented signature extension results that allow us to reduce certain properties on arbitrary terms to the corresponding properties on ground terms. These allow FORT-h to participate in categories other than $\textsf{GCR}$ in the Confluence Competition. We presented a certificate language in which certificates for the yes/no output of the decision procedure can be expressed. These certificates are validated by FORTify, the verified Haskell program obtained from the executable Isabelle formalization. FORT-h supports properties like commutation that involve multiple TRSs. Witness generation is useful to gain insight in why a particular property holds. The synthesis mode is used to find small TRSs that satisfy a given property. FORT-s supports several options to control the (infinite) search space. We showed that the synthesis problem is undecidable, already for ARSs, by a reduction from PCP.

Comprehensive experimental results were presented, including a comparison with the tools ACP [2], AGCP [1], CoLL [49], CSI [44] that compete with FORT-h in CoCo. Full details are available from the web site https://fortissimo.uibk.ac.at/ which additionally provides a convenient interface to FORT-h, FORT-s and FORTify, as well as precompiled binaries for the three tools.

Linear variable-separated TRSs are a proper extension of left-linear right-ground TRSs. Dropping either restriction, one quickly faces an undecidable first-order theory, even when one-step rewriting ($\rightarrow ^{})$ is the only predicate. This was first shown by Treinen [54]. Related undecidability results are presented in [39, 55]. In particular, Marcinkowski [39] showed that the first-order theory of one-step rewriting is undecidable for right-ground TRSs.

Many concrete properties expressible in the first-order theory of rewriting are known to be decidable for much larger classes of rewrite systems. For instance, termination is known to be decidable for right-linear right-shallow TRSs, a result by Godoy et al. [25], extending the earlier decision result for right-ground systems of Dershowitz [14]. Termination is also decidable for almost-orthogonal growing TRSs [43]. Confluence is decidable for right-linear shallow TRSs [24] and for right-ground TRSs [30].

For ground TRSs, which are in the scope of FORT-h, termination is known to be decidable in polynomial time [45]. The same holds for confluence [7]. Felgenhauer [19] showed that confluence can be decided in cubic time. Similar complexity results for the related properties $\textsf{NFP}$, $\textsf{UNC}$ and $\textsf{UNR}$ are given in [20]. The worst-case complexity of the formalized decision procedure implemented in FORT-h is at least double exponential (cf. [26]).

Concerning synthesis, we are not aware of any other tree-automata based tool for synthesizing TRSs nor of any tool that allows properties to be specified by an arbitrary first-order formula in the theory of rewriting. Jiresch [29] developed a synthesis tool to attack the well-known open problems [15, 16] concerning the sufficiency of certain restricted joinability conditions on critical pairs of left-linear TRSs. Zantema [56] developed the tool Carpa+ for synthesizing TRSs that satisfy properties which can be encoded as SMT problems. The TRSs that can be synthesized form a small extension of the class of ARSs: A single unary function symbol *f* is permitted and rules must have the shape $a \rightarrow b$, $a \rightarrow f(b)$, or $f(a) \rightarrow b$, where *a* and *b* are constants. The properties are restricted to those that can be encoded into the conjunctive fragment of SMT-LRA (linear real arithmetic). The predecessor tool Carpa [57] synthesized combinations of ARSs with help of a SAT solver. It was used to show the necessity of certain conditions in abstract confluence results [52, Sect. 5] and inspired us to support multiple TRSs in FORT.

Concerning future work, improving the efficiency of FORT-h by supporting parallelism might result in a speed-up, especially for larger formulas. The minimization of tree automata (also non-deterministic ones) is an obvious target for further investigation. Preprocessing techniques that go beyond the mere transformation to negation normal form will be helpful to obtain equivalent formulas that reduce the size of the ensuing tree automata in the decision procedure. In [28] similar ideas are applied to WS*k*S, in connection with MONA [31]. An interesting question is whether FORT-h can be extended to deal with properties involving innermost and other restrictions of rewriting. Formalization efforts that aim to transfer code in module A to the verified code in module B in Fig. 7, are also of interest. The conversion of FORT syntax to de Bruijn notation is a natural candidate here.

## Data Availability

The experiments summarized in the manuscript are available from https://fortissimo.uibk.ac.at/jar. The same holds for binaries and sources of the artifacts.

## Appendix A: Input Format

The input format of FORT-h can be roughly split into two parts: The logical structure of the property and the involved atomic predicates and relations. The logical structure is defined by the following grammar, where angle brackets $\langle \textit{~}\rangle$ are used for non-terminal symbols:

Here $\langle \textit{nat}\rangle$ is a natural number, $\langle \textit{var}\rangle$ is an alphanumerical string representing a variable name and $\langle \textit{trss}\rangle$ is a comma separated list of indices referencing TRSs. The logical operators are all right-associative. Regarding precedence the unary operations bind strongest with the binary operators respecting the order $\texttt { \&  } \,>\, \texttt {| } \,>\, \texttt {=> } \,>\, \texttt {<=> }$. Most represented operations have the meaning expected from a first-order formula, the exception being the operations $\texttt {\{+ } \langle \textit{nat}\rangle \texttt {\} }\, \langle \textit{formula}\rangle$, which allows the user to specify the number of constants to be added to the signature when evaluating the subformula, and $\texttt {[ } \langle \textit{trss}\rangle \texttt {] }\, \langle \textit{formula}\rangle$, which restricts and permutes the indices of TRSs for the underlying subformula.

The atomic binary relations supported by FORT-h are defined as:

$$\begin{aligned} \langle \textit{relation}\rangle ~::=~&\texttt {->e } ~|~ \texttt {->e* } ~|~ \texttt {->e= }~|~ \texttt {->e+ } ~|~ \texttt {e<- } ~|~ \texttt {*e<- } ~|~ \texttt {=e<- } ~|~ \texttt {+e<- } \\ ~|~&\texttt {->be } ~|~ \texttt {->be* } ~|~ \texttt {->be= } ~|~ \texttt {->be+ } ~|~ \texttt {be<- } ~|~ \texttt {*be<- } ~|~ \texttt {=be<- } ~|~ \texttt {+be<- } \\ ~|~&\texttt {-> } ~|~ \texttt {->* } ~|~ \texttt {->= } ~|~ \texttt {->+ } ~|~ \texttt {<- } ~|~ \texttt {*<- } ~|~ \texttt {=<- } ~|~ \texttt {+<- } \\ ~|~&\texttt {->! } ~|~ \texttt {-||-> } ~|~ \texttt {!<- } ~|~ \texttt {<-||- } ~|~ \texttt {<-> } ~|~ \texttt {<->* } \\ ~|~&\texttt {= } ~|~ \texttt {join } ~|~ \texttt {meet } \end{aligned}$$

Here the $\texttt {->e }$ stands for a root step, $\texttt {->be }$ for a step below the root, $\texttt {-> }$ a normal rewrite step, $\texttt {->! }$ is a reduction to normal form, $\texttt {-||-> }$ is a parallel step, $\texttt {join }$ stands for joinability $\downarrow$ and $\texttt {meet }$ for meetability $\uparrow$. The suffix $\texttt {* }$ stands for the transitive-reflexive, $\texttt {+ }$ for the transitive, and $\texttt {= }$ for the reflexive closures.

### Example 38

Consider calling FORT-h with three input TRSs on the formula: "{+2} forall s, t ([2,0] ([0] s ->! t <=> [1] s ->! t))" The {+2} instructs FORT-h to add two constants to the signature when constructing the automata. Normally "[0] s ->! t" means that term *s* normalizes to term *t* in the first input TRS (the one with index 0), however here the context has changed due to the restrict modifier [2,0], which permutes and restricts the three TRSs in the subformula ([0] s ->! t <=> [1] s ->! t) such that [0] refers to the TRS with index 2 and [1] refers to the TRS with index 0. So FORT-h checks normalization equivalence of the third and first input TRS, while ignoring the second one. The two constants are added according to Table 3, since one of the involved TRSs may be linear variable-separated.

It is also possible to use some predefined properties by name. Here we differentiate between properties of terms and properties of whole TRSs.

$$\begin{aligned} \langle \textit{property}\rangle ~::=~&\langle \textit{prop}\_\textit{of}\_\textit{term}\rangle ~|~ \langle \textit{prop}\_\textit{of}\_\textit{system}\rangle \end{aligned}$$

The properties on whole TRSs have the same names as defined in Sect. 6.

$$\begin{aligned} \langle \textit{prop}\_\textit{of}\_\textit{system}\rangle ~:=~&\texttt {CR } ~|~ \texttt {WCR } ~|~ \texttt {SCR } ~|~ \texttt {NFP } ~|~ \texttt {UNC } ~|~ \texttt {UNR } ~|~ \texttt {WN } ~|~ \texttt {SN } \\ ~|~&\texttt {GCR } ~|~ \texttt {GWCR } ~|~ \texttt {GSCR } ~|~ \texttt {GNFP } ~|~ \texttt {GUNC } ~|~ \texttt {GUNR } \\ ~|~&\langle \textit{binary}\_\textit{prop}\rangle \, \texttt {( } \texttt {[ } \langle \textit{trss}\rangle \texttt {] } \texttt {, } \texttt {[ } \langle \textit{trss}\rangle \texttt {] } \texttt {) } \\ \langle \textit{binary}\_\textit{prop}\rangle ~::=~&\texttt {COM } ~|~ \texttt {GCOM } ~|~ \texttt {CE } ~|~ \texttt {GCE } ~|~ \texttt {NE } ~|~ \texttt {GNE } \end{aligned}$$

The term properties take a variable as an additional argument.

Note that the $\texttt {INF }$ and $\texttt {FIN }$ properties also take a binary relation as an argument. This is usually one of the predefined rewrite relations, but may also be a more complex relation constructed by combining the rewrite relations using logical operators.

The property names (with exception of $\texttt {NF }$ and $\texttt {INF }$) are all just a shorthand for larger formulas. In general these correspond to the definitions of the property in Sect. 6. However there are some exceptions. Take for example ground-confluence ($\texttt {GCR }$). This unfolds to the formula
The s -> u on the left of the implication differs from the original definition of $\texttt {GCR }$. However this property (known as semi-confluence [3]) can be shown to be equivalent to $\texttt {GCR }$ by a simple induction proof, and generally leads to smaller automata in the decision procedure. The runtime comparison between different representations of ground-confluence and other properties is shown in Sect. 8.

## Appendix B: User Interface of FORT-h

The command-line interface of FORT-h is fort-h [OPTIONS] FORMULA TRS.trs .. where TRS.trs .. is one ore more files containing TRSs in the COPS format used in CoCo. It also supports *many-sorted* TRSs in the MSTRS format in the $\textsf{GCR}$ category. The additional options are

Witness generation enables the tool to produce witnesses/counterexamples and will be described in detail later in this section. For now, consider Example 28 and the call
So in addition to the answer NO, it also outputs a counterexample for the given formula consisting of the two terms g(_00()) and g(_01()). Here _00 and _01 are additional constants required to reduce confluence to ground-confluence, and represent variables. The terms should therefore be read as $\textsf{g}(x)$ and $\textsf{g}(y)$.

## Appendix C: User Interface of FORT-s

The command-line interface of FORT-s is given below: fort-s [OPTIONS] FORMULA [TRS.trs ..] where [TRS.trs..] are zero or more files containing TRSs, and the options are

The signature used during synthesis can be specified in multiple ways, the two simplest being with the command line flags -S and -a. With the option -S the signature is specified by a string listing the symbols in $\mathcal {F}$ together with their arities, like in the call Since we often do not care about the presentation of function symbols it is also permitted to just list arities with the option -a: FORT-s then generates unique symbol names for the user. If no signature is given, FORT-s generates successive signatures in a systematic manner with the help of a *signature step* and a bound on the maximal arity. If the signature step number is set to 1 and the arity is bounded by 3, signatures with the following arities are created:

$$\begin{aligned}&\{0\}, \{0,1\}, \{0,1,2\}, \{0,1,2,3\}, \{0,0,1,2,3\}, \{0,0,1,1,2,3\}, \dots \end{aligned}$$

If the signature step is set to 2 (its default value), we obtain

$$\begin{aligned}&\{0\}, \{0,0\}, \{0,0,1\}, \{0,0,1,1\}, \{0,0,1,1,2\}, \dots , \\&\{0,0,1,1,2,2,3,3\}, \{0,0,0,1,1,2,2,3,3\}, \dots \end{aligned}$$

The signature step is passed to FORT-s with the option -s and the bound on the arities by -A. Note that when additional systems are passed to FORT-s, it will use the union of the signatures of those systems.

When synthesizing *n* TRSs, in the given formula the indices 0 through $n-1$ refer to the systems to be generated, and the indices greater than $n-1$ refer to systems passed as additional inputs to FORT-s.

## Footnotes

1 http://cl-informatik.uibk.ac.at/isafor/

2 https://www.isa-afp.org

3 Since states are terms from $T_{\uparrow } \subseteq \mathcal {T}(\mathcal {F}_\bot )$ here, Definition 14 applies.

4 http://project-coco.uibk.ac.at/

5 https://fortissimo.uibk.ac.at/tacas2021/

6 https://fortissimo.uibk.ac.at/jar

7 https://cops.uibk.ac.at/

8 https://cops.uibk.ac.at/results/?y=2019 &c=COM

9 http://zenon.dsic.upv.es/confident
